# Identification of nuclear-enriched miRNAs during mouse granulopoiesis

**DOI:** 10.1186/1756-8722-7-42

**Published:** 2014-05-15

**Authors:** Justin JL Wong, William Ritchie, Dadi Gao, Katherine A Lau, Maria Gonzalez, Anupma Choudhary, Ryan J Taft, John EJ Rasko, Jeff Holst

**Affiliations:** 1 Gene & Stem Cell Therapy Program, Centenary Institute, Camperdown, Australia; 2 Sydney Medical School, University of Sydney, Sydney, Australia; 3 Bioinformatics Laboratory, Centenary Institute, Camperdown, Australia; 4 Institute for Molecular Bioscience, University of Queensland, St. Lucia, Queensland, Australia; 5 Cell and Molecular Therapies, Royal Prince Alfred Hospital, Camperdown, Australia; 6 Origins of Cancer Laboratory, Centenary Institute, Camperdown, Australia

**Keywords:** miRNAs, mRNA targets, Nuclear, Granulopoiesis, Gene expression, Stem cell

## Abstract

**Background:**

MicroRNAs (miRNAs) are coordinators of cellular differentiation, including granulopoiesis. Although differential expression of many miRNAs is associated with the maturation of granulocytes, analysis of differentially expressed miRNAs and their cellular localization across all stages of granulopoiesis, starting from hemopoietic stems cells, is not well characterized.

**Methods:**

We analyzed whole cell miRNA and mRNA expression during granulopoiesis using Taqman low-density and Affymetrix arrays respectively. We also performed nuclear and cytoplasmic fractionation followed by Taqman low-density array and/or quantitative PCR to identify nuclear-enriched miRNAs in hemopoietic stem/progenitor cells, promyelocytes, myelocytes, granulocytes and several hemopoietic cell lines. Anti-correlation between the expression of miRNA and target pairs was used to determine putative miRNA targets.

**Results:**

Analyses of our array data revealed distinct clusters of differentially expressed miRNAs that are specific to promyelocytes and granulocytes. While the roles of many of these miRNAs in granulopoiesis are not currently known, anti-correlation of the expression of miRNA/mRNA target pairs identified a suite of novel target genes. Clusters of miRNAs (including members of the let-7 and miR-17-92 families) are downregulated in hemopoietic stem/progenitor cells, potentially allowing the expression of target genes known to facilitate stem cell proliferation and homeostasis. Additionally, four miRNAs (miR-709, miR-706, miR-690 and miR-467a*) were found to be enriched in the nucleus of myeloid cells and multiple hemopoietic cell lines compared to other miRNAs, which are predominantly cytoplasmic-enriched. Both miR-709 and miR-706 are nuclear-enriched throughout granulopoiesis and have putative binding sites of extensive complementarity downstream of pri-miRNAs. Nuclear enrichment of miR-467a* is specific to hemopoietic stem/progenitors and promyelocytes. These miRNAs are also nuclear-enriched in other hemopoietic cell lines, where nuclear sequestering may fine-tune the expression of cytoplasmic mRNA targets.

**Conclusions:**

Overall, we have demonstrated differentially expressed miRNAs that have not previously been associated with hemopoietic differentiation and provided further evidence of regulated nuclear-enrichment of miRNAs. Further studies into miRNA function in granulocyte development may shed light on fundamental aspects of regulatory RNA biology and the role of nuclear miRNAs.

## Introduction

MicroRNAs (miRNAs) are 22-24 nucleotide non-coding RNAs that participate in the regulation of mRNA expression in eukaryotes [1-3], and play critical roles in a wide range of biological processes including cell-cycle control [4,5], immune response [6-8], and differentiation [9-11]. One of the best-characterized differentiation processes is granulopoiesis, in which hemopoietic myeloid progenitor cells develop sequentially from myeloblasts into morphologically distinct promyelocytes, myelocytes and mature granulocytes. This process is tightly controlled by changes in the expression of hundreds of transcription factors [12,13], which are in turn regulated by a few highly expressed miRNAs, including miR-223 and miR-146a, both of which have been shown to promote granulopoiesis [14-16]. Total loss of miR-223 does not completely block granulopoiesis [14], suggesting that other miRNAs may also act in concert to maintain this process. Few studies, however, have sought to completely profile differentially expressed miRNAs during granulopoiesis in primary cells. Prior work has either been restricted to differentiated cell lines [17,18], in which accurate modelling of the specific stages of granulopoiesis is not entirely possible, or human neutrophil maturation [19,20].

In the most well-accepted models, miRNAs bind loosely to complementary sequences in the 3′UTR of their target mRNAs in the cytoplasm, and function by inhibiting translation, inducing mRNA cleavage or mRNA degradation following decapping and deadenylation [21]. It is important to correlate the expression of miRNAs and their target mRNAs during granulopoiesis in order to obtain insights into the role of these molecules in the development of granulocytes. While previous studies have investigated this relationship in human cells [22], the miRNA-mRNA interaction network in mouse granulopoiesis has been largely uncharacterized.

Several studies have reported localization of miRNAs in the nucleus [23-31], suggesting these molecules may have other biological functions or mechanisms of action apart from their canonical role. For example, miRNAs have been shown to target gene promoters, potentially inducing overexpression (miR-373) or downregulation (miR-320) of target genes [32,33]. More recently, mouse-specific miR-709 was found to be enriched in the nucleus to target pri-miR-15a and pri-miR-16, thus regulating the expression of mature miR-15a and miR-16 [31]. With one exception [31], studies on nuclear-enriched miRNAs have been performed in cell lines. It remains unclear whether nuclear enrichment of some miRNAs is a feature of transformed cells, and whether differential expression of nuclear miRNAs is important in regulating gene expression during cellular differentiation or transformation.

In this study, we analyzed miRNA expression in primary murine myeloid cells at four successive stages of hemopoietic differentiation; Lin^-^ Sca1^+^ cKit^+^ stem/progenitor cells (LSK), promyelocytes, myelocytes and granulocytes. We not only performed analyses of miRNA expression levels in whole cells, allowing direct interrogation of miRNA-mRNA expression relationships, but also analyzed purified nuclear and cytoplasmic cell fractions to profile miRNA subcellular localization. We found four nuclear-enriched miRNAs in primary cells and further assessed their subcellular distribution in a range of mouse hemopoietic cell lines.

## Results

### Differential expression of whole cell miRNAs during primary murine granulopoiesis

**Figure 1:**
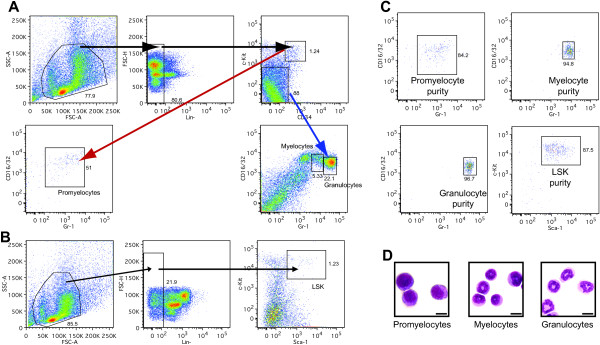
**Purification of LSK cells, promyelocytes, myelocytes and granulocytes from mouse bone marrow.** Gating strategy for fluorescence activated cell sorting (FACS) of promyelocytes (red arrow), myelocytes (left box, blue arrow) and granulocytes (right box, blue arrow) **(A)**, LSK **(B)**, and the total purity of each cell population based on re-analysis following FACS **(C)** are shown. Promyelocytes, myelocytes and granulocytes were deposited onto poly-L-lysine slides, stained using May-Grünwald Giemsa, and morphology examined using a light microscope at 100× magnification **(D)**. Scale bars indicate 10 μm.

**Figure 2:**
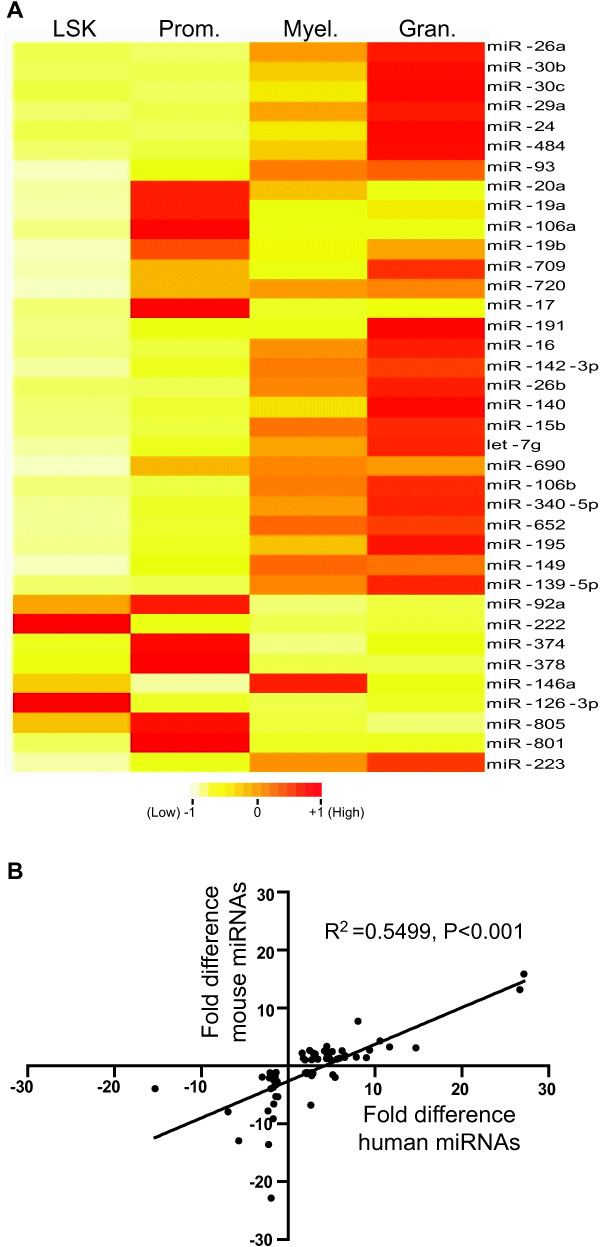
**miRNA expression during mouse and human granulopoiesis. (A)** Differentially expressed miRNAs between two or more stages of granulopoiesis. Heatmap shows highly expressed miRNAs (CT < 25 in at least one cell type) that displayed differential expression between two or more cell types. The level of miRNA expression is represented by a color scale where yellow indicates lower-level expression, orange indicates medium expression and red indicates higher expression. **(B)** Correlation between the expression levels of differentially-expressed miRNAs during mouse and human granulopoiesis. A significant correlation was found between the fold-differential expression of 64 miRNAs common to both mouse and human datasets (P < 0.001, Spearman’s correlation). Prom; promyelocytes. Myel; myelocytes, Gran; granulocytes.

We first used Taqman Low Density Quantitative Reverse Transcription PCR array (TLDA RT-qPCR) to determine the expression of 585 mature mouse and rat miRNAs in whole cell RNA from four primary murine hemopoietic populations: LSK, promyelocytes, myelocytes and granulocytes (Figure 1). A previous report showed that RT-qPCR results are only reliable when the cycle threshold (CT) is less than 30 [34], and therefore we only considered the differential expression of miRNAs when the CT value was <30 in at least one cell type. 129 mouse miRNAs showed differential expression by 2-fold or more between two or more stages of murine granulocytic differentiation (Additional file 1). An unsupervized hierarchical clustering analysis revealed 37 highly expressed miRNAs (CT <25 in at least one cell type) that were differentially regulated between two or more differentiation stages (Figure 2A). Of these, we observed that several had been previously identified as key modulators of granulopoiesis in human and mouse, including miR-223, miR-16, and miR-29a [14,35,36].

Comparison of our data with a recently reported miRNA expression profile of human granulopoiesis [19], restricted to the 64 differentially expressed miRNAs interrogated by both human and mouse TLDA experiments, indicated a significant correlation between the expression of miRNAs during mouse and human granulopoiesis (Figure 2B; R^2^ = 0.5499, P < 0.001). Of 78 miRNAs that were upregulated in mouse granulocytes compared to promyelocytes (fold change >2), 41 were interrogated in the human TLDA assay, and 30 (73%) were upregulated (Additional file 2). Similarly, 36 miRNAs were downregulated in mouse granulocytes (fold change < -2) and 23 of these (64%) were common to both datasets and also downregulated in human samples (Additional file 2).

### Identification of miRNA targets in primary murine myeloid cells

**Figure 3:**
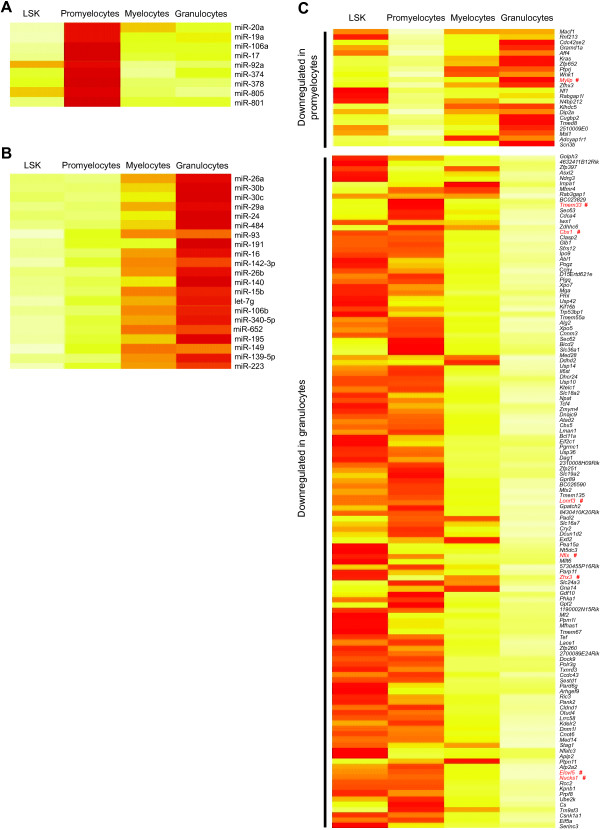
**Stage specific changes in miRNA expression throughout granulopoiesis and their putative targets in promyelocytes and granulocytes.** miRNAs that were expressed highest in promyelocytes **(A)** or granulocytes **(B)** are shown together with their predicted targets according to TargetScan **(C)**. Targets were only displayed if they were expressed lowest in the same tissue where miRNA expression was the highest. This facilitates the visualization of putative miRNA-mRNA pairs that were specific to promyelocytes or granulocytes. #, Target mRNAs that are known validated targets (Tarbase) of the stage specific miRNAs.

We next examined mRNA expression using Affymetrix microarray analysis for each of the four murine hemopoietic populations (LSK, promyelocytes, myelocytes and granulocytes). We then used these data to determine the possible miRNA targets that were differentially regulated during granulopoiesis (promyelocytes vs granulocytes) using our method based on conserved anti-correlation [37]. There were 67 predicted mRNA targets in our microarray dataset that demonstrated an inverse correlation with the expression of an individual miRNA (Additional file 3). mRNA targets that showed inversely correlated expression with miRNAs (Additional file 3) include previously validated miRNA/target pairs such as *Mef2c* with miR-223 [14], *Bcl2* with miR-15 or miR-16 [38], *Mybl2* with miR-29 or miR-30 family members [39], and *Ezh2* with miR-26a [40]. In order to identify miRNA-target signatures that may distinguish stem and committed myeloid progenitor cells (LSK vs granulocytes), we again searched for differentially expressed miRNAs and their targets that demonstrated inverse correlation in expression levels. A subset of miRNAs were downregulated in LSKs compared to promyelocytes including members of the let-7 family and the polycistronic mir-17-92 cluster (Additional file 4). These miRNAs also shared common targets including *Hlf*, *Mycn* and *Klf12* (Additional file 4).We then further refined our analysis to concentrate on miRNA/target pairs that displayed expression patterns specific to one stage of granulopoiesis (Figure 3). Expression of a group of 9 miRNAs, which showed the highest level of expression in promyelocytes (Figure 3A), was inversely correlated with a total of 22 predicted or previously confirmed mRNA targets (Figure 3C). Expression of 21 granulocyte-enriched miRNAs (Figure 3B) was inversely correlated with the downregulation of 125 putative or confirmed mRNA targets (Figure 3C).

### Nuclear and cytoplasmic localization of miRNAs in murine myeloid cells

**Figure 4:**
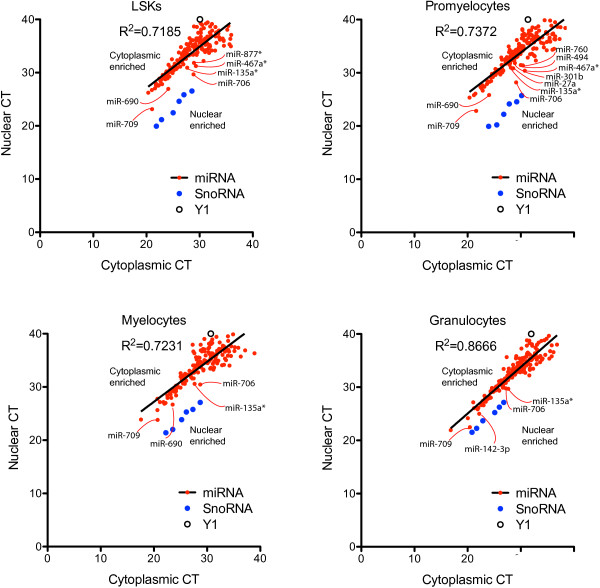
**Cytoplasmic:nuclear expression of miRNAs in primary mouse myeloid cells.** CT data from cytoplasmic and nuclear TLDA miRNA analysis from LSK cells, promyelocytes, myelocytes and granulocytes are shown based on cell equivalent volumes to detect nuclear-enriched miRNAs. Solid line indicates linear regression analysis of miRNA expression with goodness of fit (R^2^) values shown. miRNAs showing decreased nuclear CT (increased nuclear expression) are labelled. Y1 RNA CT (cytoplasmic control) and SnoRNA CT (nuclear control) are also shown.

**Figure 5:**
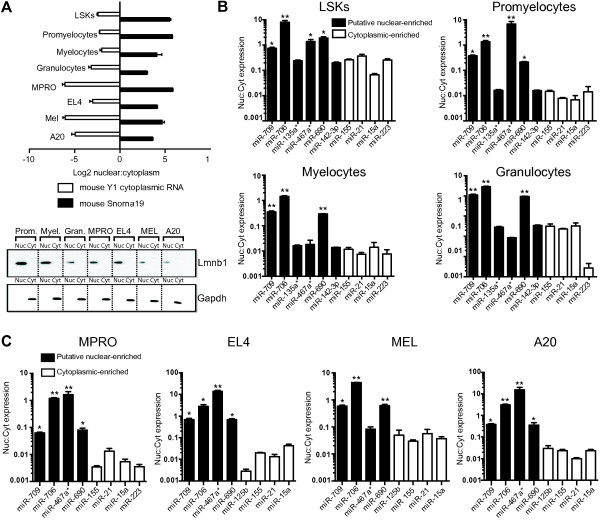
**Nuclear enrichment of miRNAs in primary mouse myeloid cells and hemopoietic cell lines. (A)** RT-qPCR of known nuclear- and cytoplasmic-specific RNA in nuclear and cytoplasmic RNA fractions in mouse primary cells and cell lines. Lower panels show representative western blots for nuclear-specific Lmnb1 and cytoplasmic-specific Gapdh protein in the nuclear and cytoplasmic fractions. Both RT-qPCR and western blotting confirm the range of detection and enrichment of nuclear and cytoplasmic fractions **(B)** Nuclear-enriched miR-709, miR-706, miR-467a* and miR-690 in primary mouse LSK cells, promyelocytes, myelocytes and granulocytes. **(C)** Nuclear-enriched miRNAs in mouse hemopoietic cell lines, MPRO, EL4, MEL and A20. (* P < 0.05; ** P < 0.01, t-test). Prom; promyelocytes. Myel; myelocytes, Gran; granulocytes.

In order to determine the sub-cellular localization of miRNAs, we performed nuclear and cytoplasmic fractionation on LSK, promyelocytes, myelocytes and granulocytes, extracted the RNA, and analyzed miRNA expression by TLDA RT-qPCR (Figure 4). Purity of nuclear and cytoplasmic fractions was determined using RT-qPCR to assess the expression of the nuclear specific *SnoRNA19*, which was enriched in the nuclear RNA samples by 8- to 56-fold (Figures 4 and 5A), and *Y1* cytoplasmic RNA, which was enriched in the cytoplasmic RNA pools by 4- to 9-fold (Figure 5A). Western blot was also performed to confirm the purity of nuclear and cytoplasmic fractions (Figure 5A). The nuclear lamina protein, Lmnb1 and the cytoplasmic protein, Gapdh were enriched in the nuclear and cytoplasmic fractions respectively, indicating the purity of these fractions. Almost all miRNAs were distributed towards the upper left quadrant, confirming that the vast majority of miRNAs are enriched in the cytoplasm (Figure 4). Linear regression analysis of miRNAs showed correlation of the miRNA cytoplasmic and nuclear expression levels (R^2^ = 0.7185-0.8666) suggesting that the low level of nuclear expression (relative to cytoplasmic expression) is predominantly due to low level contamination of the nuclear fraction with cytoplasmic miRNAs.

In order to determine whether some miRNAs were bona fide nuclear expressed, we focused on the ~60 mature miRNAs that were expressed in the nucleus of at least one cell type with a CT < 30 (Additional file 5). The majority of highly-expressed miRNAs (CT < 30) displayed nuclear: cytoplasmic ratio of <0.1, indicating there was 10-fold more miRNA expression in the cytoplasm compared to the nucleus. We performed additional regression analysis of the ratio of nuclear to cytoplasmic expression of miRNAs and found six highly-expressed miRNAs (CT < 30) that trended towards being nuclear-enriched (ratio nuclear: cytoplasmic expression > 0.1) in one or more cell types. Amongst these 6 miRNAs, miR-706 and miR-467a* (now renamed miR-467a-3p) had nuclear:cytoplasmic ratios > 1 in promyelocytes (Additional file 5).We next performed individual Taqman miRNA RT-qPCR assays to validate the nuclear enrichment of these six miRNAs (miR-706, miR-467a*, miR-709, miR-690, miR-135a* (now renamed miR-135a-1-3p) and miR-142-3p) compared to that of highly cytoplasmic-enriched control miRNAs identified in the TLDA assays in (Figures 4 and 5B). Three of these (miR-706, miR-709 and miR-690) were found to be enriched in the nucleus of all four cell types studied (ratio nuclear:cytoplasmic expression was significantly greater that of the mean nuclear-cytoplasm of controls, p < 0.05). Expression of miR-467a*, interestingly, was enriched only in the nucleus of LSK and promyelocytes, while expression of miR-135* and miR-142-3p did not appear to be nuclear-enriched in any myeloid population (Figure 5B).

### Nuclear expression of miR-709, miR-706, miR-690 and miR-467a* in hemopoietic cell lines

In order to characterize the extent of the nuclear expression of these miRNAs, we analyzed the expression of miR-709, miR-706, miR-690 and miR-467a* in four mouse hemopoietic cell lines: MPRO, EL4, MEL and A20. The purity of the subcellular fractions was confirmed using RT-qPCR (Figure 5A), as described above. The nuclear:cytoplasmic expression of miR-709, miR-706 and miR-690 was significantly greater in the nucleus of all four cell lines compared to cytoplasmic-enriched control miRNAs (p < 0.05) (Figure 5C). miR-467a* was significantly enriched in the nucleus of MPRO, EL4 and A20 (p < 0.05) (Figure 5C).

### Predicted pri-miRNA targets of nuclear-enriched miR-709, miR-706, miR-467a* and miR-690

**Figure 6:**
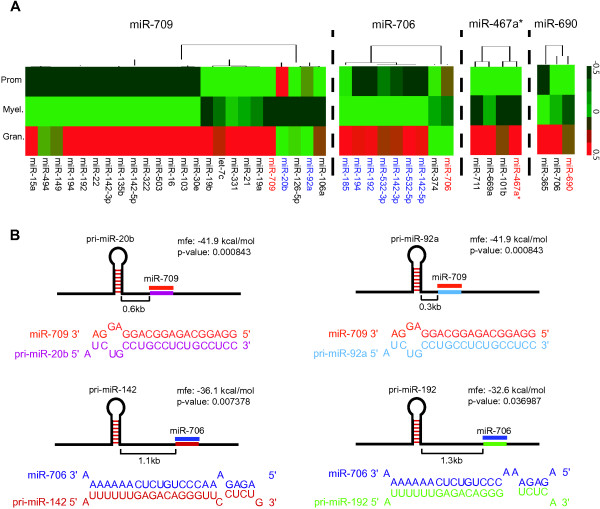
Predicted pri-miRNA targets of nuclear-enriched miR-709, miR-706, miR-467a* and miR-690 during mouse granulopoiesis. (A) Heatmaps showing differential expression of nuclear miRNAs (name in red) in promyelocytes, myelocytes and granulocytes together with the expression of mature miRNAs (name in black; inverse correlated in blue), processed from predicted pri-miRNAs targets of respective nuclear-enriched miRNAs. **(B)** Putative binding site for miR-709 on pri-miR-20b and pri-miR-92a, and miR-706 on pri-miR-142 and pri-miR-192 as predicted by RNAhybrid. Near perfect complementarity between nuclear-enriched miRNA and putative pri-miRNA target was found in each case.

A previous study has shown that miR-709 is nuclear-enriched and targets other pri-miRNAs in the nucleus, thereby downregulating the expression of their mature forms [31]. Therefore, these nuclear-enriched miRNAs detected in hemopoietic cells may act as negative regulators of other miRNAs. We predicted pri-miRNA targets of the four nuclear-enriched miRNAs: miR-709, miR-706, miR-467a* and miR-690 during mouse granulopoiesis using RNAhybrid [41]. We correlated the expression of nuclear-enriched miRNAs with the expression of mature miRNAs (in the cytoplasm), in cases where their primary transcripts were predicted targets of each of the four nuclear-enriched miRNAs. We only considered candidates that were predicted to hybridize with respective nuclear-enriched miRNAs with a minimum free energy (MFE) of < -30 kcal/mol, and a high probability of binding as determined using RNAcalibrate (p < 0.05) [41]. Upregulation of miR-709 from promyelocytes to granulocytes correlated with the downregulation of mature miR-20b and miR-92a (Figure 6A); putative binding sites of both pri-miRNA-20b and 92a demonstrated near perfect complementarity to mature miR-709 (Figure 6B). Downregulation of nuclear-enriched miR-706 from promyelocytes to granulocytes occurred in conjunction with the upregulation of 7 mature miRNAs (Figure 6A). Of these 7 miRNAs, miR-142 and miR-192 possessed putative binding sites in their primary transcripts that demonstrated near perfect binding to mature miR-706 (Figure 6B). The expression of nuclear-enriched miR-690 and miR-467a* did not show anti-correlation with that of mature miRNAs processed from their putative pri-miRNA targets (Figure 6A).

### Validation of miRNA function in the nucleus

**Figure 7:**
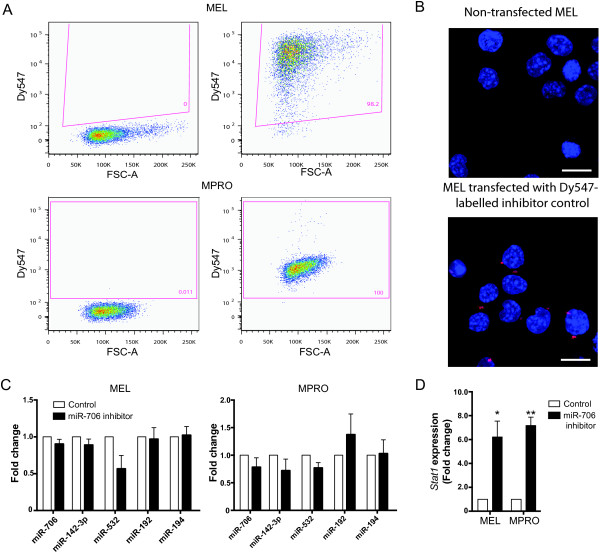
**The effect of miR-706 inhibition on the expression of putative target miRNAs and mRNAs in MEL and MPRO cells. (A)** Representative plots showing high efficiencies of transfection of Dy547-labelled inhibitor control in MEL and MPRO cells (right) compared to non-transfected controls (left), indicating that miR-706 hairpin is likely to be transfected at similar high efficiencies. **(B)** Immunofluorescent microscopy of MEL cells showing cytoplasmic localization of transfected inhibitor control (red), with nuclei counterstained by Hoechst 33342 (blue). **(C)** Expression of miR-706, miR-142-3p, miR-532, miR-192 and miR-194 in cells transfected with miR-706 hairpin inhibitor compared to control. **(D)** Expression of *Stat1* in MEL and MPRO cells transfected with miR-706 inhibitor and control. (*P < 0.05; **P < 0.01, t-test).

In order to study whether miR-706 can target pri-miRNAs (like miR-709) [31], we transfected cell lines with a miRNA inhibitor and examined mature target miRNA levels. Using a labelled miRNA, we achieved >95% transfection efficiency (Figure 7A), although expression was limited to the cytoplasm (Figure 7B). miRNA inhibitors can either bind to and degrade the mature miRNA, resulting in lower miRNA levels by RT-qPCR, or form a stable heteroduplex [42], which can inhibit function while still allowing detection by RT-qPCR. Analysis of miR-706 levels showed no significant decrease in expression, suggesting that this inhibitor may form a heteroduplex (Figure 7C). However, inhibition of miR-706 function did not lead to a significant increase in the expression of predicted miRNA targets in MEL cells (Figure 7C). Analysis of miR-706 knockdown in MPRO cells showed a ~1.5-fold increase in miR-192 levels, however this increase was not statistically significant (Figure 7C).

We also considered that miRNAs may be retained in the nucleus to prevent them from targeting mRNAs in the cytoplasm. Relevant to our study, miR-709 has previously been shown to target Myc, which is downregulated during myeloid cell differentiation [43]. We observed higher expression of cytoplasmic miR-709 in granulocytes (CT: 20.432) compared to promyelocytes (CT: 21.599) (Additional file 5) suggesting that a greater amount of miR-709 may be present in mature granulocytes to sequester Myc expression. miR-706 has previously been shown to regulate the expression of myeloid transcription factor *Stat1*[44]. We therefore determined its expression in myeloid cell lines MEL and MPRO following inhibition of miR-706. Knockdown of miR-706 resulted in a significant upregulation of *Stat1* by 6 to 8-fold in both cell lines, indicating its role in the regulation of this transcription factor (P < 0.05) (Figure 7D). It is therefore possible that retention of miR-706 in the nucleus, which would result in decreased cytoplasmic miR-706 expression, may act to fine-tune expression of target genes such as *Stat1*.

## Discussion

Previous studies have shown the involvement of several miRNAs in granulopoiesis including miR-223 [14], miR-34a [45], and miR-146a [16]. However, a systematic expression profiling of miRNAs and their mRNA targets across successive populations of myeloid cells at defined stages of granulopoiesis is lacking. Comprehensive miRNA expression profiling of human granulocytic differentiation was recently published, and showed that the potential roles of many miRNAs in granulopoiesis, either individually or as a group, are currently ignored [19,20].

Our study sought to provide the first comprehensive characterization of miRNA expression across carefully purified cells at progressive stages of murine granulopoiesis. In addition, we correlated miRNA expression with their predicted and confirmed mRNA targets to determine the possible role of differentially expressed miRNAs during granulopoiesis.

Amongst miRNAs that showed differential expression between mouse promyelocytes and granulocytes, we noted many that have been shown to be associated with hematological malignancies (Figure 2A). Several miRNAs that were upregulated during granulopoiesis (miR-15a, miR-16 and miR-29) have previously been shown to be downregulated in acute myeloid leukemia [46,47]. miRNAs that were downregulated during granulopoiesis, including miR-17, miR-19a and miR-20a, were amongst those previously shown to be upregulated in leukemia [48]. These data suggest the control of miRNA expression is crucial in ensuring proper cell differentiation, and failure of this control may lead to, or contribute to, cancer.

Many miRNAs that we identified as being differentially expressed in granulopoiesis were not previously implicated in this process including miR-19a, miR-19b miR-24, miR-26a, miR-26b, miR-93, miR-106b, miR-191, miR-139-5p, miR-140 and miR-195 (Figures 2A and 3, Additional file 1). Notably, several miRNAs shared common predicted targets with miRNAs that are known to be important for granulopoiesis (Additional file 3). For example, miR-106b and miR-194 were predicted to target the transcription factor gene, *Mef2c,* which is a confirmed target of miR-223 [14,49]. Repression of *Mef2c* is important for early granulopoiesis and may also be involved in the regulation of granulocyte activation [14]. Expression of miR-106b and miR-194 may act in concert with that of miR-223, which may explain a previous observation whereby granulopoiesis was not completely impaired in miR-223 null mice [14].

A recent study reported a plethora of differentially expressed miRNAs during human granulopoiesis, many of which were predicted *in silico* to target key transcription factors such as *Runx1* and *Pu.1*[20]. Our stringent analysis that considered anti-correlation between miRNA and mRNA target expression did not reveal any miRNAs predicted to regulate key transcription factors such as *Cebpα*, *Pu.*1 or *Runx1* during granulopoiesis, suggesting that essential transcription factors are not likely to be directly regulated by miRNAs during this process (data not shown). However, the transcription factor *Myb* was downregulated with concurrent overexpression of miR-15b, miR-16, miR-150 and miR-195 (Figure 3 and Additional file 3). *Myb* is involved in hemopoietic progenitor proliferation [50], and may need to be downregulated to allow terminal differentiation of granulocytes. We also observed downregulation of transcription factors such as *Mga* and *Tcf4*, and myeloid leukemia related genes including *Pml*, *Abl1*, and *Bcl11a* in terminally differentiated granulocytes in conjunction with the expression of a group of granulocyte-enriched miRNAs (Figure 3). Future studies are required to determine whether downregulation of these genes is important for normal granulopoiesis and whether they are regulated by miRNAs.

Several members of the polycistronic miR-17-92 cluster and the homologous miR-106a-92 cluster (miR-17, miR-19a, miR-20a, miR-92a and miR-106a) were expressed at the highest levels in promyelocytes (Figure 3B). These miRNAs are known to have oncogenic potential and are overexpressed in hematological malignancies [48,51,52]. Overexpression of these miRNAs is also known to confer a stem cell-like phenotype [51]. While our data was consistent with others in that these miRNAs were highly expressed in early developmental lineages [51], it was surprising that their expression levels were lower in LSK compared to promyelocytes. These data are the first to indicate that the miR-17-92 polycistron is predominantly over-expressed in promyelocytes during granulopoiesis. In relation to leukaemia, overexpression of miR-17-92 may reflect accumulation of blast cells and promyelocytes in myeloid malignancies such as acute and chronic myeloid leukemia [53]. Furthermore, we and others have previously shown that miR-17-92 expression is downregulated following imatinib treatment in chronic myeloid leukaemia patients [54,55]; this observation may reflect the normalization of blood cell proportions following hematological response after treatment [56]. In line with previous reports [57,58], we detected downregulation of confirmed miR-17-92 targets including *Pten* and *E2f2* in promyelocytes compared to granulocytes (Additional file 3), although the significance of this observation in the context of granulopoiesis remains unclear.

Consistent with others [59-61], we found the highest expression of miR-125a, mir-125b, let7d and let7e in LSK and promyelocytes compared to differentiated cells (Additional file 1). In addition, our present data indicate the downregulation of miR-17-92 and let-7 families in LSK compared to promyelocytes, in conjunction with the overexpression of their reciprocal targets including hepatic leukemia factor (*Hlf*), V-myc myelocytomatosis viral related oncogene (*Mycn*) and krüppel-like factor 12 (*Klf12*) (Additional file 4). *Hlf* is a transcription factor that can confer anti-apoptotic effects and prevent premature death of hemopoietic stem cells [62]. *Mycn* is an oncogene which is important for the proliferation and homeostasis of stem cells [63]. The role of the transcription factor, *Klf12* in stem cell development is as yet unknown. However, other members of krüppel-like factor family such as *Klf*4 and *Klf*5 are key players in embryonic stem cell renewal and somatic cell reprogramming [64,65]. Whether *Klf12* has a role similar to *Klf*4 and *Klf*5 remains to be elucidated.

We have also carefully purified the nuclear and cytoplasmic RNA from four populations of murine primary hemopoietic cells to study the localization of miRNAs in these cells. This step is important given the recent reports of nuclear-enriched miRNAs that could possibly be involved in non-canonical functions [32,33]. These miRNAs are unlikely to regulate gene expression by targeting the 3′ UTR of target mRNAs but may target the promoter of genes [32,33], or other pri-miRNAs [31]. Alternatively, these miRNAs may be retained in the nucleus to prevent them from downregulating a potential target mRNA in the cytoplasm [23].

It is important to consider the fact that perfect nuclear/cytoplasmic fractionation is technically difficult and thus rigid analysis is required to obtain meaningful data [30]. We only considered the highly expressed miRNAs in each sample (expression was detected below 30 cycles of RT-qPCR amplification in at least one cell type) to minimize possible false positive results. Furthermore, by using cell equivalent volumes, and comparing to the mean expression of cytoplasmic enriched miRNAs, we further reduced false positive results. Our discovery of four nuclear-enriched miRNAs is similar to a recent study in primary mouse liver, where three nuclear-enriched miRNAs were found [31]. These data suggest that the majority of miRNAs are acting in a canonical manner by targeting the 3′UTR of granulopoiesis-regulating genes. It is also noteworthy that the majority (73%) of the differentially expressed miRNAs are conserved between mouse and human (Figure 2B and Additional file 2), and this could mean they are likely to have common canonical mRNA targets [66].

All four nuclear-enriched miRNAs, miR-709, miR-706, miR-690 and miR-467a* in hemopoietic cells are mouse specific miRNAs. It is interesting to note the enrichment of miR-706 and miR-467a* in the nucleus of primary mouse hemopoietic cells and cell lines because these miRNAs have not been reported as being nuclear-enriched in other cell types [23-31]. Notably, the primary transcript of miR-142-3p, a miRNA recently found to be important in promoting granulopoieisis, was amongst the putative targets of miR-706 (Figure 6B). In contrast to the previous finding in mouse liver, we did not observe an anti-correlation between the expression of nuclear miR-709 and cytoplasmic miR-15a/16 in myeloid cells. We discovered two other predicted pri-miRNA targets of miR-709 based on our *in silico* analysis using RNAhybrid and anti-correlation of miRNA expression. This result suggests that interaction between this nuclear-enriched miRNA and its pri-miRNA targets may be cell type specific. Inhibition of miR-706 in MEL and MPRO cells, however, did not result in significant accumulation of predicted miRNA targets, indicating either these miRNAs are not the actual targets or nuclear miR-706 may not function by targeting pri-miRNA transcripts. Alternatively, since the miRNA inhibitor was expressed in the cytoplasm, it is possible that there was little to no reduction in nuclear miR-706 expression levels in these cells. Whether miR-706 and other nuclear-enriched miRNAs target pri-miRNAs during hemopoiesis remains to be determined.

miRNAs may also be retained in the nucleus to fine-tune the expression of their mRNA target(s). Apart from the validated role of miR-709 in regulating the expression of *Myc*, knockdown of miR-690 has recently been shown to increase the expression of a key myeloid transcription factor *Cebpα*[67]. Consistent with others [44], we have shown that miR-706 regulates the expression of *Stat1*; a transcription factor involved in myeloid differentiation. The retention of miR-709, miR-690 and miR-706 in the nucleus may be important to control the expression of transcription factors or other proteins during granulopoiesis.

## Conclusions

Overall, we have provided a comprehensive characterization of miRNAs and mRNA target expression during mouse granulopoiesis, as well as examining the global nuclear and cytoplasmic abundance of miRNAs during granulopoiesis. Furthermore, we have examined the nuclear and cytoplasmic expression of miRNAs in mouse primary myeloid cells and hemopoietic cell lines. We showed that miRNAs are predominantly expressed in the cytoplasm of primary myeloid cells from mouse and thus, they are likely to induce canonical post-transcriptional gene silencing by targeting 3′UTRs. There were four nuclear-enriched miRNAs that are ubiquitously present in mouse hemopoietic cells, including two miRNAs, miR-467a* and miR-706 that have never been reported to be nuclear-enriched. These miRNAs may be constrained to the nucleus to fine-tune the expression of their mRNA targets. We have also provided a comprehensive profiling of miRNAs and their targets in the course of murine granulopoiesis, which highlights clusters of miRNAs that may be regulating this process. Conditional knockout of Dicer-1 in myeloid progenitors has recently been described [68]. Dicer-1 depletion leads to neutrophil dysplasia with marked loss of expression of a large number of miRNAs. Amongst these are those differentially regulated in our study including miR-16, miR-19a, miR-26a, miR-26b, miR-139, miR-195 and miR-223. The roles of many of these miRNAs and their targets in granulopoiesis are currently unknown and are therefore interesting candidates for future studies.

## Materials and Methods

### Primary cells and cell lines

Bone marrow was harvested from the femur, tibia and spine of C57BL/6 mice as previously described [69-71]. Primary mouse hemopoietic stem/progenitor cells (Lin^-^Sca1^+^cKit^+^; LSK), promyelocytes, myelocytes and granulocytes were isolated using established fluorescence–activated cell sorting (FACS) protocols [71-73]. For the isolation of LSK cells, bone marrow cells were incubated with lineage specific antibodies (B220, Ter119, CD3, Gr-1, CD11b; BioLegend), conjugated to biotin, together with anti–Sca1–fluorescein isothiocyanate (FITC; Biolegend) and anti–cKit–phycoerythrin (PE; Becton Dickinson). Antibodies used for the isolation of promyelocytes, myelocytes and granulocytes were lineage specific antibodies (B220, CD19, CD3, Sca1; BioLegend), conjugated to biotin, together with anti–Gr–1–FITC (Biolegend), anti–cKit–PE (Becton Dickinson), anti–CD34–Alexafluor 647 (eBioscience) and anti–CD16/32–PerCP/Cy5.5 (Becton Dickinson) antibodies. Cells were washed twice in PBS with 2% (v/v) FCS and incubated with streptavidin–APC–Cy7 (Biolegend). Control stains for FITC, PE, PerCP/Cy5.5, Alexafluor 647 and APC–Cy7 were used to determine compensation settings and gating for each population. The FACS gating strategies for the four cell types are shown in Figure 1A and B.Purity of over 84% was achieved for all cell types (Figure 1C). Morphological confirmation of promyelocytes, myelocytes and granulocytes was performed following May-Grünwald Giemsa staining of cells spun onto poly-L-lysine slides (Figure 1D).

MEL and EL4 cells were grown in Dulbecco’s Modified Eagle's Medium (DMEM) supplemented with 10% fetal calf serum (FCS). A20 cells were maintained in RPMI 1640 media containing 10% FCS. MPRO cells were maintained in DMEM media supplemented with 5% FCS plus 10% BHK-HM5 conditioned medium. All growth media was supplemented with 5 U/mL penicillin, 5 μg/ml streptomycin sulfate and 2 mM L-glutamine.

### Preparation of nuclear, cytoplasmic and total RNA

Nuclear and cytoplasmic fractions were separated using the PARIS kit (Ambion) with RNAse inhibitors to minimize RNA degradation. RNA was extracted from either the nucleus, cytoplasm or whole cells using Trizol (Invitrogen) according to the manufacturer’s instructions. Purity of the nuclear and cytoplasmic RNA was confirmed by comparing the abundance of snoRNA or Y1 cytoplasmic RNA using quantitative PCR (qPCR) as previously described [26,29].

### Western blot to assess the purity of the nuclear and cytoplasmic fractions

Nuclear and cytoplasmic cell lysates were loaded onto precast SDS-PAGE gels (Invitrogen) and transferred onto PVDF membranes. Membranes were blocked with 5% skim milk and incubated overnight at 4°C with a polyclonal rabbit anti-Lmnb1 antibody (1:5,000; Abcam) or monoclonal mouse anti-Gapdh antibody (1:1,000; Abcam), followed by a HRP-conjugated secondary anti-rabbit or anti-mouse antibody (1:5,000; Chemicon). HRP-conjugated antibodies were detected using enhanced chemiluminescence reagents (Pierce) on a Kodak Imager (Kodak).

### Taqman low-density array

Taqman low-density array (TLDA) was performed using TaqMan® Array Rodent MicroRNA A + B Cards Set v2.0 (Applied Biosystems) as previously described [55]. Samples used were whole cell, cytoplasmic and nuclear RNA from LSK, promyelocytes, myelocytes and granulocytes. Briefly, reverse transcription reaction was performed using rodent Megaplex™ RT primers (Applied Biosystems), which contain a pool of 750 individual miRNA-specific primers, according to the manufacturer’s instructions. Real-time quantitative PCR (RT-qPCR) was then carried out on an ABI 7900HT real-time PCR machine with the LDA thermal cycler block, using pre-defined TLDA thermal cycling conditions. RT-qPCR data were analyzed using SDS 2.3 and RQ Manager Software (Applied Biosystems). The whole cell and cytoplasmic miRNA CT values correlated with R^2^ values of 0.9126 (LSK), 0.9112 (promyelocyte), 0.9244 (myelocyte) and 0.9406 (granulocyte). Raw data were deposited in the Gene Expression Omnibus database (accession number GSE57624).

### Microarray and data analysis

Differential expression of mRNAs during differentiation of hemopoietic stem/progenitor cells into granulocytes was determined using Affymetrix GeneChip Gene 1.0 ST mouse arrays according to the manufacturers’ instructions. Briefly, 300 ng of total RNA from 3 biological replicates of LSK cells, promyelocytes, myelocytes or granulocytes was used for the synthesis of double-stranded cDNA using random hexamers tagged with a T7 promoter sequence. Amplification of the double-stranded cDNA was then performed using T7 RNA polymerase to produce cRNA. cRNA was subjected to a first-strand cDNA synthesis with incorporation of dUTP residues to produce single-stranded DNA. Fragmentation of single-stranded DNA was subsequently performed using a mixture of uracil DNA glycosylase and apurinic/apyrimidinic endonuclease 1, which can recognize unnatural dUTP residues and induce breakage to the DNA. Single-stranded DNA was assessed using RNA 6000 Nano Chip on an Agilent 2100 Bioanalyzer (Agilent Technologies), and successfully fragmented DNA was labelled with terminal deoxynucleotidyl transferase with a DNA labelling reagent (Affymetrix). Samples were injected into Affymetrix array chips and hybridized at 45°C and 60 rpm for 17 hours in a hybridization oven (Affymetrix). Arrays were stained and washed in the Affymetrix GeneChip Fluidic Station 450 and scanned using Affymetrix GeneChip Scanner 3000 7G.

The arrays were normalized using the justRMA suite of algorithms from Bioconductor (http://www.bioconductor.org/). Genes with a minimal fold change in expression of 2 and a moderated P-Value of 0.05 using empirical Bayes shrinkage were considered significantly differentially expressed as previously reported [74]. Raw data were deposited in the Gene Expression Omnibus database (accession number GSE57624).

### Bioinformatics analyses

mRNA and miRNA expression across samples was compared and negative correlations were determined as previously published [37]. All mRNA/miRNA pairs with significant changes in expression between promyelocytes and granulocytes in opposite directions were considered as potential functional interactors. These pairs were filtered further by requiring a perfect match between the seed region of each miRNA and the 3′UTR of the paired mRNA. These seed matches were either an 8mer match, a 7mer-m8 site or a 7mer-A1 site.

### Taqman PCR assay for miRNA

Quantitative TaqMan*®* MicroRNA Assay (Applied Biosystems) was performed according to the manufacturer’s instructions using primer and probes that specifically detect individual miRNAs of interest. For the measurement of miRNA expression in the nuclear and cytoplasmic fractions, a total of 6 μl was used for each cell-equivalent fraction of nuclear and cytoplasmic RNA. Fold expression of miRNA in the nucleus was calculated as a ratio over its expression in the cytoplasm.

### Prediction of pri-miRNA targets of nuclear-enriched miRNAs

Putative pri-miRNA targets of nuclear-enriched miRNAs were predicted using RNAhybrid (http://bibiserv.techfak.uni-bielefeld.de/rnahybrid) [41]. Primary transcripts of all miRNAs annotated in miRBase were considered in this analysis.

### Inhibition of miR-706 function

MEL and MPRO cells were transfected with the 2′-O-methylated miRIDIAN mmu-miR-706 hairpin inhibitor (Thermo Scientific) or the miRIDIAN miRNA hairpin inhibitor transfection control with Dy547. The optimal concentration of inhibitor for each cell type was determined based on the percentage of Dy547-positive cells measured using a BD Biosciences LSR Fortessa Analyzer following transfection with different concentrations of dye-labelled control. The optimal concentration of inhibitor for MEL and MPRO were 80 and 300 nM respectively. For each transfection reaction, 1x10E5 cells were plated per well in a six-well plate containing 800 μl of antibiotic-free DMEM with 10% FCS. Inhibitor was diluted to a final volume of 185 μl with DMEM, while 4 μl Oligofectamine (Invitrogen) was diluted to 15 μl of DMEM at room temperature for 5 mins. The two mixtures were then combined and incubated for another 20 mins at room temperature before being added to the cells. Transfection mixtures were replaced with fresh DMEM containing 10% FCS after four hours, and incubated for 48 hours prior to harvest. Immunofluorescent microscopy was performed using a Leica SP5 Confocal microscope to determine the localization of Dy547-labelled inhibitor control in transfected cells. Total RNA was extracted and miRNA expression was measured using individual TaqMan® MicroRNA Assays (Applied Biosystems). *Stat1* expression was measured using standard RT-qPCR.

### Statistical analyses

The significance of fold change in miRNA expression was analyzed using the Wilcoxon signed rank test applied to the ΔCT values. Correlation analyses were computed using the Spearman rank correlation test. Student’s t-tests were used to compare the expression ratio of nuclear-enriched miRNAs with cytoplasmic enriched controls. T-tests were also used to compare the expression of *Stat1* in cell lines transfected with miR-706 inhibitors and control. Analyses were performed using GraphPad Prism v.5 (La Jolla, CA, USA). Results were considered significant when *P* value < 0.05.

## Competing interests

The authors declared no competing interests.

## Authors' contributions

JJ-LW, KAL, MG, AC, and JH performed experiments. WR and DG performed bioinformatic analyses. JJ-LW and JH designed the study with input from JEJR and RJT. JJ-LW, RJT, JEJR and JH wrote or edited the manuscript. All authors read and approved the final manuscript.

## Supplementary Material

Additional file 1 Differential miRNA expression between cell types. Click here for file

Additional file 2 Differentially expressed miRNAs during mouse and human granulopoiesis. Click here for file

Additional file 3 Differentially expressed miRNAs in granulocytes compared to promyelocytes that have predicted mRNA targets showing inversely correlated expression. Click here for file

Additional file 4 Differentially expressed miRNAs in LSK compared to promyelocytes that have predicted targets showing inversely correlated expression. Click here for file

Additional file 5 Nuclear to cytoplasmic ratio of mouse miRNAs during granulopoiesis. Click here for file
