# Supplementary material for: Identification of nuclear-enriched miRNAs during mouse granulopoiesis
Source: J Hematol Oncol. 2014 May 15;7:42. doi: 10.1186/1756-8722-7-42 (PMC4046156; doi:10.1186/1756-8722-7-42)
Supplement: Additional file 1 — Differential miRNA expression between cell types. [file 1756-8722-7-42-S1.pdf]

Additional file1- Differential miRNA expression between cell types

	Log 2 fold change					
mirName	Promyelocyte/LSK	Myelocyte/Promyelocyte	Granulocyte/Myelocyte	Myelocyte/LSK	Granulocyte/ Promyelocyte	Granulocyte/LSK
mmu-let-7a	2.064918689	-0.007060282	-2.102186035	2.057858407	-2.109246317	-0.044327628
mmu-let-7c	0.898177232	-0.915836484	-1.066222192	-0.017659252	-1.982058677	-1.083881444
mmu-let-7d	1.903540823	-0.699241404	-1.354274845	1.204299419	-2.053516249	-0.149975426
mmu-let-7e	0.010769921	-0.100057696	-0.695258242	-0.089287775	-0.795315938	-0.784546017
mmu-let-7g	1.447272259	1.273534627	0.231467829	2.720806886	1.505002456	2.952274715
mmu-let-7i	2.49503173	-0.508801846	-1.006389297	1.986229884	-1.515191143	0.979840587
mmu-miR-101a	0.958522981	-1.639985467	-0.821762078	-0.681462486	-2.461747545	-1.503224564
mmu-miR-101b	0.964164368	-2.062234756	-0.933393165	-1.098070388	-2.995627921	-2.031463553
mmu-miR-103	0.424129751	2.589858321	0.458149026	3.013988072	3.048007347	3.472137099
mmu-miR-106a	1.61087249	-1.204060088	-0.790990875	0.406812402	-1.995050962	-0.384178473
mmu-miR-106b	0.149753189	2.059055065	-0.177960125	2.208808254	1.88109494	2.030848129
mmu-miR-106b*	6.554266291	3.09536081	-1.280423957	9.649627101	1.814936853	8.369203144
mmu-miR-10b	-1.731222085	3.729589499	1.825758059	1.998367414	5.555347558	3.824125473
mmu-miR-125a-5p	-10.7347052	-1.048836463	1.453768402	-11.78354167	0.40493194	-10.32977326
mmu-miR-125b-5p	-3.915293711	-0.272889287	-2.403230973	-4.188182998	-2.676120261	-6.591413971
mmu-miR-126-3p	-5.196572423	-0.816684829	0.266854713	-6.013257253	-0.549830117	-5.74640254
mmu-miR-126-5p	-4.754834706	-0.932247793	-1.396841676	-5.687082498	-2.329089469	-7.083924174
mmu-miR-130a	-1.791567833	-3.045887553	-1.911062673	-4.837455386	-4.956950226	-6.748518059
mmu-miR-130b	2.036711753	-1.199273456	-0.463106609	0.837438296	-1.662380065	0.374331687
mmu-miR-130b*	0.011966579	-1.185255464	0.025642669	-1.173288885	-1.159612794	-1.147646215
mmu-miR-135a	12.28386434	8.348859959	1.087591084	20.63272429	9.436451043	21.72031538
mmu-miR-135a*	0.584652862	0.60631237	0.188046242	1.190965231	0.794358612	1.379011473
mmu-miR-135b	2.442549733	6.161369311	2.69196743	8.603919044	8.853336741	11.29588647
mmu-miR-139-3p	-0.4239588	14.92688846	3.315426198	14.50292966	18.24231466	17.81835586
mmu-miR-139-5p	1.198709317	8.358946076	1.555313373	9.557655393	9.914259449	11.11296877
mmu-miR-140	0.753039724	1.804850736	1.538902064	2.55789046	3.343752801	4.096792524
mmu-miR-142-3p	3.574417159	3.174340232	0.048721072	6.748757391	3.223061303	6.797478462
mmu-miR-142-5p	1.240250442	3.671808017	-0.621920208	4.912058459	3.04988781	4.290138251
mmu-miR-146a	-3.900591913	5.025911911	-3.324999462	1.125319998	1.70091245	-2.199679464
mmu-miR-148a	2.905485391	1.135918968	0.049404876	4.041404359	1.185323844	4.090809235
mmu-miR-149	6.472209749	2.546607685	-0.830822488	9.018817434	1.715785198	8.187994947
mmu-miR-150	-6.658888382	5.458589219	-1.175289013	-1.200299163	4.283300206	-2.375588176
mmu-miR-155	-2.635040704	-2.602782226	-2.83112165	-5.237822931	-5.433903876	-8.068944581
mmu-miR-15a	0.059832895	4.528956979	0.221894566	4.588789874	4.750851544	4.81068444

mmu-miR-15a*	1.506079448	1.368754406	0.748765946	2.874833853	2.117520351	3.623599799
mmu-miR-15b	0.824155394	3.784806714	0.171805885	4.608962107	3.956612598	4.780767992
mmu-miR-15b*	4.013932512	1.305502488	0.312840566	5.319435	1.618343054	5.632275566
mmu-miR-16	0.717823791	3.416065128	0.552514049	4.133888919	3.968579178	4.686402969
mmu-miR-16*	2.271940506	1.315759556	1.023997264	3.587700062	2.339756819	4.611697325
mmu-miR-17	1.818578112	-1.629557448	-0.266854713	0.189020663	-1.896412161	-0.077834049
mmu-miR-181a	0.938179797	-0.402641194	-1.376327541	0.535538602	-1.778968735	-0.840788938
mmu-miR-185	3.637498126	3.029373674	-1.323845544	6.6668718	1.70552813	5.343026256
mmu-miR-186	-1.399747845	0.990097655	0.80552172	-0.409650191	1.795619375	0.39587153
mmu-miR-18a	5.015706129	-1.320135905	-1.042972839	3.695570224	-2.363108743	2.652597385
mmu-miR-191	0.507041049	-0.113733786	0.619355941	0.393307263	0.505622155	1.012663204
mmu-miR-192	2.350065172	2.960480369	0.05453341	5.310545541	3.015013779	5.365078951
mmu-miR-194	-0.0377802	4.453738482	1.508301812	4.415958282	5.962040294	5.924260095
mmu-miR-195	2.256725856	2.179233709	1.531893068	4.435959565	3.711126777	5.967852633
mmu-miR-196b	-6.382802309	-3.361634289	-4.038720426	-9.744436597	-7.400354714	-13.78315702
mmu-miR-19a	2.919674334	-1.451084469	-0.458490928	1.468589865	-1.909575398	1.010098937
mmu-miR-19b	1.958928989	-0.716165565	-0.371134901	1.242763423	-1.087300467	0.871628522
mmu-miR-200c	5.313673947	4.08397119	1.067589801	9.397645137	5.151560991	10.46523494
mmu-miR-203	-4.128127866	-1.789225803	-0.165822595	-5.917353669	-1.955048398	-6.083176264
mmu-miR-20a	3.367566293	-1.127815884	-1.611727245	2.239750409	-2.73954313	0.628023163
mmu-miR-20a*	0.644485757	-2.417813105	-0.682095005	-1.773327348	-3.09990811	-2.455422353
mmu-miR-20b	0.285830288	-2.278316983	-0.390281428	-1.992486695	-2.668598411	-2.382768123
mmu-miR-21	1.52608073	1.028219756	-1.66848302	2.554300486	-0.640263264	0.885817466
mmu-miR-22	2.916426263	5.789550605	1.639421328	8.705976868	7.428971933	10.3453982
mmu-miR-221	2.758809322	-1.425612751	-0.10752826	1.333196571	-1.533141011	1.225668311
mmu-miR-222	-3.144304118	-1.100634655	-0.409086052	-4.244938773	-1.509720707	-4.654024825
mmu-miR-223	7.267303451	3.695228322	0.778340491	10.96253177	4.473568813	11.74087226
mmu-miR-24	-0.645853366	1.849810883	1.011004978	1.203957517	2.860815861	2.214962495
mmu-miR-24-2*	-0.7419279	3.194512465	1.589845501	2.452584564	4.784357966	4.042430065
mmu-miR-25	2.41023997	1.28772357	-0.278308438	3.69796354	1.009415132	3.419655102
mmu-miR-26a	-0.876808341	3.333495733	0.506357244	2.456687391	3.839852977	2.963044636
mmu-miR-26b	-0.17112208	4.239194815	0.612688847	4.068072735	4.851883662	4.680761582
mmu-miR-26b*	-0.095732632	3.780874838	1.09921576	3.685142205	4.880090598	4.784357966
mmu-miR-27a	2.932837571	2.466773508	-0.111460136	5.39961108	2.355313372	5.288150943
mmu-miR-27a*	2.152274716	-0.123648952	0.670128426	2.028625764	0.546479475	2.69875419
mmu-miR-27b	5.283757499	-0.51102421	-1.359061477	4.772733289	-1.870085687	3.413671812
mmu-miR-28	-1.864222063	3.165621724	2.227322262	1.301399661	5.392943985	3.528721922

mmu-miR-296-5p	-2.14526572	6.697813954	-1.355984356	4.552548235	5.341829598	3.196563878
mmu-miR-297a*	3.513045704	-2.168224456	4.054960783	1.344821248	1.886736327	5.399782031
mmu-miR-29a	-0.091971707	1.49816441	0.062226211	1.406192703	1.560390621	1.468418914
mmu-miR-29c	-1.305895675	2.428651406	0.942282624	1.122755731	3.37093403	2.065038355
mmu-miR-301a	1.950552383	2.670205352	0.020685087	4.620757735	2.690890438	4.641442822
mmu-miR-301b	3.19302519	2.232570461	-0.240357288	5.425595651	1.992213174	5.185238364
mmu-miR-30a	-0.770476739	1.934773594	-0.012308481	1.164296855	1.922465113	1.151988374
mmu-miR-30a*	-1.733444449	-0.605731136	1.511207982	-2.339175585	0.905476846	-0.827967604
mmu-miR-30b	-0.194713336	2.427283797	1.179904693	2.232570461	3.607188491	3.412475154
mmu-miR-30c	-0.928435582	2.034609054	1.172724746	1.106173471	3.207333799	2.278898217
mmu-miR-30d	-0.128555249	1.304818683	0.607560313	1.176263434	1.912378996	1.783823747
mmu-miR-30e	-0.716456182	1.209940807	0.594055174	0.493484624	1.80399598	1.087539798
mmu-miR-30e*	0.292326431	1.471325083	0.466696583	1.763651514	1.938021666	2.230348096
mmu-miR-31	-6.769664714	5.044032731	-9.496677126	-1.725631983	-4.452644395	-11.22230911
mmu-miR-320	-0.692523024	-2.355928796	-0.209586084	-3.04845182	-2.56551488	-3.258037904
mmu-miR-322	-0.749278799	3.696083078	-0.087356027	2.946804279	3.608727051	2.859448252
mmu-miR-322*	-3.788277022	4.524512249	1.69412569	0.736235228	6.218637939	2.430360918
mmu-miR-328	-0.495416372	0.925478128	1.219736306	0.430061756	2.145214434	1.649798062
mmu-miR-33*	-17.04040855	15.81754384	1.53514114	-1.222864712	17.35268498	0.312276428
mmu-miR-331-3p	-2.689745066	0.784272495	-0.064106673	-1.905472571	0.720165822	-1.969579244
mmu-miR-340-3p	0.744321216	3.07108575	1.346240142	3.815406966	4.417325892	5.161647108
mmu-miR-340-5p	2.508365918	3.111943069	0.924332755	5.620308987	4.036275825	6.544641742
mmu-miR-342-3p	-1.929183492	3.355548428	-2.157574201	1.426364936	1.197974228	-0.731209265
mmu-miR-350	-1.770540844	4.786751282	1.579417482	3.016210437	6.366168764	4.595627919
mmu-miR-365	0.729106566	4.905391365	2.568027862	5.634497931	7.473419227	8.202525793
mmu-miR-374	0.56926726	-1.443391669	-0.05299485	-0.874124409	-1.496386519	-0.927119259
mmu-miR-378	1.32316174	-2.578507166	-0.796632262	-1.255345427	-3.375139428	-2.051977688
mmu-miR-425	0.392332841	1.48602688	0.20138043	1.878359722	1.68740731	2.079740152
mmu-miR-425*	0.011966579	-0.963018996	-0.598328952	-0.951052417	-1.561347948	-1.549381369
mmu-miR-466d-3p	-21.71421242	0.019949997	23.93999612	-21.69426243	23.95994612	2.245733698
mmu-miR-467a	2.907707755	-2.033344015	5.091437479	0.87436374	3.058093464	5.965801219
mmu-miR-467a*	3.342094575	0.411428082	1.31119516	3.753522657	1.722623243	5.064717818
mmu-miR-467b*	2.99164476	0.402880526	1.907814601	3.394525286	2.310695127	5.302339887
mmu-miR-467c	2.650426306	-5.477496414	8.582772389	-2.827070108	3.105275975	5.755702282
mmu-miR-484	0.228903562	1.97802423	1.013911147	2.206927792	2.991935377	3.220838939
mmu-miR-494	8.536615584	-4.089373245	-5.523943836	4.447242339	-9.613317081	-1.076701497
mmu-miR-503	-1.136312155	5.179596976	-0.830480585	4.043284821	4.349116391	3.212804236

mmu-miR-503*	-3.487403034	2.517545993	0.865012713	-0.969857041	3.382558707	-0.104844327
mmu-miR-532-3p	-2.801376153	3.377088271	-0.386349552	0.575712118	2.990738719	0.189362566
mmu-miR-532-5p	-1.680620551	3.230754104	0.268222322	1.550133554	3.498976426	1.818355875
mmu-miR-574-3p	1.108959975	1.548424042	1.810201506	2.657384017	3.358625549	4.467585523
mmu-miR-652	1.969186057	3.586845306	-0.251127209	5.556031363	3.335718097	5.304904154
mmu-miR-674*	-2.323225845	1.084975531	-0.283778874	-1.238250314	0.801196657	-1.522029188
mmu-miR-690	2.948906978	0.356723721	-0.871850759	3.305630699	-0.515127037	2.43377994
mmu-miR-699	-1.217172039	-4.559830753	3.607068825	-5.777002792	-0.952761928	-2.169933967
mmu-miR-706	3.383122846	0.187482103	-1.967647496	3.570604949	-1.780165393	1.602957453
mmu-miR-709	1.345385386	-0.457003654	0.275231318	0.888381733	-0.181772336	1.163613051
mmu-miR-720	2.709575397	0.17038699	-0.606876508	2.879962387	-0.436489518	2.273085879
mmu-miR-744	0.922965146	2.42318097	0.625168279	3.346146116	3.048349249	3.971314396
mmu-miR-744*	9.54420154	5.449357858	2.008675767	14.9935594	7.458033625	17.00223517
mmu-miR-7a*	0.567557749	-0.371528089	0.584652862	0.19602966	0.213124773	0.780682521
mmu-miR-801	12.40592344	-9.471256693	-0.328226168	2.934666749	-9.799482861	2.606440581
mmu-miR-805	2.574524005	-7.021527012	-2.579652539	-4.447003008	-9.601179551	-7.026655546
mmu-miR-872*	-0.034190226	-1.887864604	-1.82746757	-1.92205483	-3.715332175	-3.749522401
mmu-miR-877*	1.058187489	-0.137325042	-1.424022906	0.920862447	-1.561347948	-0.503160458
mmu-miR-92a	0.050259632	-1.523738699	-0.496271128	-1.473479067	-2.020009827	-1.969750195
mmu-miR-93	3.53373079	1.418159282	-0.438318695	4.951890072	0.979840587	4.513571377
mmu-miR-93*	0.135051392	0.859320041	-0.17266064	0.994371433	0.6866594	0.821710792
