# Supplementary material for: Identification of nuclear-enriched miRNAs during mouse granulopoiesis
Source: J Hematol Oncol. 2014 May 15;7:42. doi: 10.1186/1756-8722-7-42 (PMC4046156; doi:10.1186/1756-8722-7-42)
Supplement: Additional file 2 — Differentially expressed miRNAs during mouse and human granulopoiesis. [file 1756-8722-7-42-S2.pdf]

Additional file 2- Differentially expressed miRNAs during mouse and human granulopoiesis

	Log2 fold change	
**mirName**	**Mouse Granulocytess/Promyelocytes**	**Human Neutrophils/ Promyelocytes**
miR-340-3p	7.864745056	1.515309558
miR-103	4.513979917	1.664124317
mir-16	6.556382392	1.772799189
miR-328	3.130297986	2.057627912
miR-142-5p	4.517422967	2.191668985
miR-30e*	2.878066645	2.23186548
miR-191	1.610155475	2.234967588
miR-30c	4.815214279	2.312079819
miR-140	5.089062819	2.48983193
miR-24	4.184049733	2.573760883
miR-26a	6.222955804	2.631301474
miR-29a	2.469467531	2.68887123
miR-26b	9.380078523	2.731264635
miR-194	14.71280074	3.120518819
miR-28	11.68106573	3.283802928
miR-192	4.45399519	3.35832866
miR-200c	10.59197708	4.322648347
miR-223	8.046157621	7.709056419
miR-22	26.66915113	13.16081157
miR-365	27.15413497	15.87356324
miR-93	1.95152237	1.027918233
miR-186	2.716595943	1.05823379
miR-142-3p	4.846018794	1.172857653
miR-27a	3.408648887	1.178209217
miR-30b	5.662738857	1.18580465
miR-484	4.412511383	1.264940287
miR-195	5.906485714	1.318294542
miR-331	1.756495645	1.340397394

miR-15a	9.003587743	1.445926236
miR-15b	6.524647677	1.461865306
miR-146a	2.614255233	-6.773903349
miR-532-5p	5.419651181	-1.987091608
miR-30e	2.725838333	-1.694185756
let-29c	5.145459791	-1.539582981
miR-148a	2.121081418	-1.346824433
miR-30a	2.859969973	-1.345211124
miR-25	1.975064817	-1.226034789
let-7g	2.414626421	-1.194142681
miR-30d	2.848297813	-1.169371932
miR-106b	2.812396492	-1.12121046
miR-342	2.131989008	-1.112327048
miR-27a*	1.637051831	NA
mmu-miR-93*	1.732793817	NA
miR-135a*	1.810138391	NA
miR-30a*	1.893558509	NA
miR-15b*	2.528181431	NA
miR-425	2.599979053	NA
miR-149	2.630067817	NA
miR-467a*	2.637370031	NA
miR-106b*	2.7379574	NA
miR-297a*	2.81883689	NA
miR-301b	2.942005615	NA
miR-15a*	3.095344533	NA
miR-467b*	3.347536996	NA
miR-16*	3.387216025	NA
miR-301a	3.905480345	NA
miR-532-3p	4.410370938	NA
miR-744	4.514605731	NA
miR-467a	4.532477945	NA
miR-467c	4.620022892	NA

miR-652	5.072510689	NA
miR-574-3p	5.119844524	NA
miR-503*	5.169769651	NA
miR-322	5.666272559	NA
miR-340-5p	6.738838728	NA
miR-150	7.448758559	NA
miR-503	7.650213443	NA
miR-24-2*	9.126742323	NA
miR-26b*	9.487973629	NA
miR-296-5p	11.4414659	NA
miR-10b	12.47614223	NA
miR-322*	16.32600614	NA
miR-350	17.33240563	NA
mmu-miR-744*	26.98526594	NA
miR-135b	47.51463362	NA
miR-135a	60.18783426	NA
miR-139-5p	73.05443733	NA
miR-139-3p	2138.672706	NA
miR-126-5p	-1.960198831	-22.80236717
miR-20b	-2.249493191	-13.56125758
miR-130a	-5.689098264	-12.93423522
let-7c	-1.702907415	-9.102439167
miR-155	-6.902887633	-7.949832811
miR-20a	-2.315140902	-7.759839992
miR-19a	-1.653588201	-6.596542701
miR-196b	-15.32165249	-3.928087531
miR-18a	-1.987424424	-3.914393014
miR-92a	-1.72931418	-3.705826699
miR-17	-1.644786126	-2.457491124
miR-27b	-1.627322285	-2.128841834
miR-320	-2.157409437	-2.072202536
miR-378	-2.995714215	-1.90503698

let-7a	-1.793030464	-1.87949398
miR-130b	-1.495885758	-1.804123467
le7-7d	-1.752968413	-1.385763516
miR-101a	-2.068521568	-1.195559684
miR-19b	-1.184764239	-5.345866277
miR-221	-1.419516875	-5.295218656
miR-222	-1.406100779	-3.266964674
miR-98	-1.248676708	-2.721152219
miR-374	-1.398519141	-1.110383952
mmu-miR-801	-40.52948673	NA
mmu-miR-805	-37.39827961	NA
miR-101b	-2.56845358	NA
miR-106a	-1.711901863	NA
miR-203	-1.684359393	NA
mmu-miR-706	-1.569059433	NA
miR-181a	-1.568298305	NA
mmu-miR-877*	-1.435844982	NA
let-7i	-1.409223072	NA
miR-130b*	-1.220015942	NA
mmu-miR-699	-1.121865716	NA
let-7e	-1.052485239	NA
miR-425*	-1.435844982	NA

Key: NA- Not assessible

in red- miRNAs upregulated during mouse granulopoiesis

in green- miRNAs downregulated during mouse granulopoiesis
