# Supplementary material for: Identification of nuclear-enriched miRNAs during mouse granulopoiesis
Source: J Hematol Oncol. 2014 May 15;7:42. doi: 10.1186/1756-8722-7-42 (PMC4046156; doi:10.1186/1756-8722-7-42)
Supplement: Additional file 3 — Differentially expressed miRNAs in granulocytes compared to promyelocytes that have predicted mRNA targets showing inversely correlated expression. [file 1756-8722-7-42-S3.pdf]

Additional file 3- Differentially expressed miRNAs in granulocytes compared to promyelocytes that have predicted mRNA targets showing inversely correlated expression

Key: ↑ Upregulated
↓ Downregulated

MicroRNA name (Granulocytes/Promyelocytes expression)	Target mRNAs				
	Symbol	log Fold Change (Granulocytes/Promyelocytes)	tstat	adjPval	Probe
mmu-miR-139-5p (↑)	*Asf1a*	-2.82	-20.5	4.00E-04	10363163
	Canx	-2.83	-12.9	8.00E-04	10385599
	Dpy30	-2.75	-14.1	7.00E-04	10452867
	Elovl5	-2.94	-17.9	5.00E-04	10587284
	Gpr56	-4.29	-10.6	0.001	10574259
	Hdgf	-3.2	-20.2	4.00E-04	10493086
	Hs2st1	-2.48	-7.5	0.004	10502522
	Kbtbd8	-2.03	-10.2	0.002	10540227
	Pdik1l	-2.12	-15.5	6.00E-04	10517243
	Tcf12	-3.29	-18.4	4.00E-04	10594879
	Tgif1	-2.16	-16	5.00E-04	10452633
mmu-miR-135a (↑) mmu-miR-135b (↑)	*Abce1*	-4.57	-19.9	4.00E-04	10579874
	Bace1	-2.14	-15.4	6.00E-04	10584941
	Bzw2	-4.67	-26.8	3.00E-04	10400030
	C80913	-3.34	-16.4	5.00E-04	10562548
	Ccdc50	-2.76	-11.1	0.001	10434869
	Chek1	-4.34	-17.9	5.00E-04	10592201
	Elovl6	-3	-11.1	0.001	10495993
	Entpd4	-2.07	-11.5	0.001	10416199
	Fkbp1a	-2.11	-13.2	8.00E-04	10477012
	Gria3	-6.15	-27.8	3.00E-04	10599348
	Ints2	-3.55	-22.6	3.00E-04	10389421
	Kctd1	-2.19	-10.5	0.001	10457606
	Man1a	-4.55	-27.2	3.00E-04	10369154
	Neto2	-2.84	-12.1	0.001	10580382
	Npat	-2.68	-12.7	9.00E-04	10585358
	Nucks1	-3.98	-15.1	6.00E-04	10349733
	Parn	-3.24	-21.5	4.00E-04	10437778
	Phf17	-2.36	-16.3	5.00E-04	10491860
	Rnf26	-2.45	-11.8	0.001	10545534
	Smad5	-2.46	-9.7	0.002	10405605
	Ssr2	-3.28	-23	3.00E-04	10493259
	Syncrip	-2.82	-16.3	5.00E-04	10595604
	Tsen54	-2.3	-15.1	6.00E-04	10382610
	Tspan31	-3.83	-24.9	3.00E-04	10373027
	Ubfd1	-2.79	-16	5.00E-04	10557139
mmu-miR-365 (↑)	*Bcl2*	-2.88	-20.7	4.00E-04	10357043
	Eefsec	-2.61	-15.1	6.00E-04	10546104
	Meis1	-3.66	-9.1	0.002	10384504
	Ppp5c	-3.15	-19.4	4.00E-04	10560315
mmu-miR-194 (↑)	*Arhgap21*	-2.49	-10	0.002	10480381
	Atp5g1	-2.81	-16.4	5.00E-04	10391100
	Chd9	-2.87	-16.7	5.00E-04	10573823
	Lphn2	-3.25	-14.7	6.00E-04	10502780
	Mef2c	-2.16	-12.3	9.00E-04	10406434
	Ncl	-2.98	-17.2	5.00E-04	10356312
	Nudc	-4.19	-16	5.00E-04	10578019
	Pgm3	-3.16	-19.6	4.00E-04	10595466
	Sdad1	-4.51	-17.8	5.00E-04	10531383
	Sephs1	-2.67	-13.5	8.00E-04	10469035
	Set	-3.72	-26.8	3.00E-04	10470893
	Tfrc	-3.33	-18.9	4.00E-04	10435075
	Ube2v2	-2.02	-10.5	0.001	10437942
mmu-miR-10b (↑)	*Cbx5*	-4.33	-12.9	8.00E-04	10433088
	Cdc6	-3.33	-20.9	4.00E-04	10381072
	Eif3j	-2.69	-15.3	6.00E-04	10475405
	Elovl6	-3	-11.1	0.001	10495993
	Kpnb1	-2.25	-14	7.00E-04	10390352
	Lancl1	-2.69	-15	6.00E-04	10355266
	Mtf2	-2.19	-12.6	9.00E-04	10523905
	Zfp367	-2.71	-11.8	0.001	10410092
	Zfp706	-2.42	-11	0.001	10349049
mmu-miR-28 (↑)	*Srprb*	-3.02	-18.6	4.00E-04	10596137
mmu-miR-200c (↑)	*1110002B05Rik*	-2.8	-15.9	5.00E-04	10400321
	6330409N04Rik	-2.17	-14.3	7.00E-04	10420659
	Abi2	-2.53	-15.4	6.00E-04	10346764
	Acaca	-2.65	-13.1	8.00E-04	10379820
	Aff3	-2.59	-9.4	0.002	10354111
	Ak3l1	-1.73	-9.2	0.002	10506269
	Asf1a	-2.82	-20.5	4.00E-04	10363163
	Atp2a2	-3.07	-14.4	7.00E-04	10533483
	Bcl2	-2.88	-20.7	4.00E-04	10357043
	Calu	-3.7	-18.1	5.00E-04	10536818
	Canx	-2.83	-12.9	8.00E-04	10385599
	Cbx5	-4.33	-12.9	8.00E-04	10433088
	Ccne2	-2.7	-6.7	0.006	10503264
	Cdc25a	-2.08	-8.4	0.003	10589420
	Cdk6	-4.23	-29.9	3.00E-04	10519324
	Clic4	-2.18	-9.1	0.002	10517336
	Cnn3	-3.37	-17.3	5.00E-04	10406852
	Crtap	-3.25	-23.7	3.00E-04	10597413
	Dek	-2.92	-10.2	0.002	10409031
	Dpy19l1	-3.78	-20.8	4.00E-04	10591816
	Eif2s1	-3.46	-19.2	4.00E-04	10396795
	Eif3j	-2.69	-15.3	6.00E-04	10475405
	Epha7	-2.82	-13.3	8.00E-04	10503659
	Fgfr1op	-2.18	-15.8	5.00E-04	10441620
	Fubp1	-2.95	-19.6	4.00E-04	10496892
	Fyn	-2.13	-11.3	0.001	10362596
	Gata2	-2.63	-12.4	9.00E-04	10539873
	Gfi1	-2.03	-14.9	6.00E-04	10532124
	Hmgb3	-2.34	-10.3	0.002	10600017
	Hnrnpab	-2.84	-15	6.00E-04	10385686
	Hs2st1	-2.48	-7.5	0.004	10502522
	Hspa9	-4.9	-26.3	3.00E-04	10458226
	Ikzf2	-2.23	-12	0.001	10355312
	Mgat2	-2.48	-10.4	0.001	10396074
	Mrps25	-2.3	-14.7	6.00E-04	10546396
	Mtf2	-2.19	-12.6	9.00E-04	10523905
	Myb	-3.49	-19	4.00E-04	10368199
	Nedd1	-2.36	-13.6	7.00E-04	10371907
	Nme1	-4.5	-27.5	3.00E-04	10389865
	Nudcd1	-3.79	-14.8	6.00E-04	10428412
	Nup107	-2.71	-19.5	4.00E-04	10372687
	Pdcd4	-2.55	-16.7	5.00E-04	10463997
	Pdik1l	-2.12	-15.5	6.00E-04	10517243
	Pds5b	-2.68	-14.9	6.00E-04	10527832
	Phtf2	-2.46	-13.9	7.00E-04	10528238
	Ppm1f	-2.22	-13.2	8.00E-04	10433929
	Ptbp1	-3.3	-20.9	4.00E-04	10364518
	Qser1	-4.99	-36.7	3.00E-04	10485622
	Rab38	-3.77	-14.3	7.00E-04	10554800
	Ralgps2	-2.08	-8.7	0.003	10359201
	Rpgr	-2.17	-10.2	0.002	10603598
	Scd2	-4.45	-16.7	5.00E-04	10463355
	Sec61a2	-2.12	-9.4	0.002	10479887
	Sephs1	-2.67	-13.5	8.00E-04	10469035
	Sfxn1	-4.18	-26.3	3.00E-04	10405236
	Skp2	-2.97	-21.9	4.00E-04	10427606
	Smarcad1	-3.33	-15	6.00E-04	10538755
	Snx30	-2.4	-11.4	0.001	10505224
	Syncrip	-2.82	-16.3	5.00E-04	10595604
	Tcerg1	-3.7	-18	5.00E-04	10455346
	Tcf12	-3.29	-18.4	4.00E-04	10594879

	Tex2	-2.44	-6.5	0.007	10392207
	Tfrc	-3.33	-18.9	4.00E-04	10435075
	Trim44	-2.98	-16.7	5.00E-04	10485395
	Tubb5	-3.97	-12.5	9.00E-04	10450605
	Usp6nl	-2.57	-18.6	4.00E-04	10469110
	Zmym4	-2.3	-12.7	9.00E-04	10516435
mmu-miR-26a (↑)	*Abi2*	-2.53	-15.4	6.00E-04	10346764
mmu-miR-26b (↑)	*Acsl3*	-2.29	-14	7.00E-04	10347748
mmu-miR-26b* (↑)	*Arhgap21*	-2.49	-10	0.002	10480381
	Atp10a	-2.82	-14.8	6.00E-04	10553788
	Ccnd2	-2.71	-11.6	0.001	10548105
	Depdc6	-2.72	-8	0.003	10424126
	Dnm1l	-2.23	-15.4	6.00E-04	10437992
	Ezh2	-3.13	-20.5	4.00E-04	10544501
	Fkbp3	-4.29	-29.5	3.00E-04	10400581
	Gnpnat1	-3.55	-16.5	5.00E-04	10419216
	Grb10	-2.17	-13.6	7.00E-04	10384398
	Hmga1	-2.84	-14.9	6.00E-04	10383479
	Huwe1	-2.14	-14.8	6.00E-04	10602501
	Ints2	-3.55	-22.6	3.00E-04	10389421
	Itpr1	-3.21	-19.8	4.00E-04	10540408
	Kbtbd8	-2.03	-10.2	0.002	10540227
	Kpna2	-2.21	-9.6	0.002	10497503
	Lman1	-4.75	-31.1	3.00E-04	10459481
	Mat2a	-3.23	-17.4	5.00E-04	10545417
	Mdn1	-2.77	-18.6	4.00E-04	10503723
	Mtx2	-2.79	-14.6	6.00E-04	10472994
	Otud4	-2.03	-14.2	7.00E-04	10573027
	Pdhx	-2.35	-12.7	9.00E-04	10485429
	Pdik1l	-2.12	-15.5	6.00E-04	10517243
	Pgm2l1	-2.73	-12.2	0.001	10555303
	Phf6	-2.85	-13.2	8.00E-04	10599612
	Rcn2	-2	-10.7	0.001	10585545
	Rpgr	-2.17	-10.2	0.002	10603598
	Scoc	-2.67	-16.7	5.00E-04	10450904
	Serbp1	-2.63	-11.5	0.001	10538857
	Sgcb	-2.38	-10.1	0.002	10530633
	Skp2	-2.97	-21.9	4.00E-04	10427606
	Snx30	-2.4	-11.4	0.001	10505224
	Strbp	-2.39	-10.8	0.001	10482181
	Thap2	-2.12	-6.8	0.006	10372497
	Whsc1	-2.55	-18.6	4.00E-04	10521136
	Zc3h7b	-2.12	-14.8	6.00E-04	10425578
mmu-miR-15a (↑)	*1300001I01Rik*	-3.87	-21.5	4.00E-04	10378453
mmu-miR-15b (↑)	*Ak3l1*	-3.01	-13.6	7.00E-04	10472923
mmu-miR-15b* (↑)	*Akt3*	-2.59	-15	6.00E-04	10360506
	Atp5g1	-2.81	-16.4	5.00E-04	10391100
	Bace1	-2.14	-15.4	6.00E-04	10584941
	Bcl2	-2.88	-20.7	4.00E-04	10357043
	Cbx5	-4.33	-12.9	8.00E-04	10433088
	Ccnd2	-2.71	-11.6	0.001	10548105
	Ccne1	-2.12	-12.3	9.00E-04	10562563
	Cdc25a	-2.08	-8.4	0.003	10589420
	Chd9	-2.87	-16.7	5.00E-04	10573823
	Chek1	-4.34	-17.9	5.00E-04	10592201
	Cops7a	-2.31	-12.9	8.00E-04	10547926
	Eif2s1	-3.46	-19.2	4.00E-04	10396795
	Eif4e	-2.32	-12.4	9.00E-04	10496485
	Epha7	-2.82	-13.3	8.00E-04	10503659
	Fbxo21	-2.23	-13.4	8.00E-04	10524941
	Fkbp1a	-2.11	-13.2	8.00E-04	10477012
	Gga2	-2.73	-18.5	4.00E-04	10567626
	Gpn1	-3.05	-15.9	5.00E-04	10520800
	Hdgf	-3.2	-20.2	4.00E-04	10493086
	Hmga1	-2.84	-14.9	6.00E-04	10383479
	Hnrnpa1	-1.87	-8.4	0.003	10427885
	Isoc1	-2.92	-16.4	5.00E-04	10455912
	Kdsr	-2.12	-13	8.00E-04	10357051
	Lphn2	-2.58	-8.9	0.002	10502776
	Myb	-3.49	-19	4.00E-04	10368199
	Nup210	-3.84	-22.5	3.00E-04	10546294
	Ocrl	-3.21	-19.7	4.00E-04	10599435
	Otud4	-2.03	-14.2	7.00E-04	10573027
	Pdcd4	-2.55	-16.7	5.00E-04	10463997
	Pdia6	-3.72	-27.5	3.00E-04	10394735
	Pdik1l	-2.12	-15.5	6.00E-04	10517243
	Ptpn4	-3.07	-22	4.00E-04	10357191
	Rad23b	-2.05	-12.3	9.00E-04	10505092
	Scoc	-2.67	-16.7	5.00E-04	10450904
	Seh1l	-2.29	-15.8	5.00E-04	10456423
	Serbp1	-2.63	-11.5	0.001	10538857
	Shcbp1	-3.79	-9.2	0.002	10576883
	Sil1	-2.54	-12.7	9.00E-04	10458251
	Slc39a10	-3.88	-20.9	4.00E-04	10354389
	Smad5	-2.46	-9.7	0.002	10405605
	Sms	-4.2	-23.4	3.00E-04	10607524
	Smyd5	-3.2	-17.5	5.00E-04	10539592
	Sumo3	-2.82	-18.4	4.00E-04	10364287
	Supt16h	-2.01	-12.4	9.00E-04	10419611
	Tmem206	-2.05	-15.1	6.00E-04	10352725
	Ubfd1	-2.79	-16	5.00E-04	10557139
	Usp14	-3.74	-23.5	3.00E-04	10457409
	Vat1	-2.44	-10.4	0.001	10391454
	Wee1	-5	-16.4	5.00E-04	10556266
	Zbtb41	-2.2	-12	0.001	10350377
	Zfp326	-2.52	-13.5	8.00E-04	10523785
	Zfp367	-2.71	-11.8	0.001	10410092
mmu-miR-223 (↑)	*Acsl3*	-2.29	-14	7.00E-04	10347748
	Cbx5	-4.33	-12.9	8.00E-04	10433088
	Dusp2	-2.1	-11.7	0.001	10475782
	Hsp90b1	-2.95	-14.9	6.00E-04	10371482
	Il6st	-3.97	-9.8	0.002	10407173
	Msi2	-2.13	-13	8.00E-04	10389680
	Nutf2	-3.78	-18.5	4.00E-04	10468531
	Otud4	-2.03	-14.2	7.00E-04	10573027
	Pds5b	-2.68	-14.9	6.00E-04	10527832
	Ralgps2	-2.08	-8.7	0.003	10359201
	Sh3pxd2b	-2.08	-13.8	7.00E-04	10375065
	Syncrip	-2.82	-16.3	5.00E-04	10595604
	Ube2q2	-2.01	-6.3	0.007	10368227
	Uqcc	-2.8	-15.3	6.00E-04	10488944
mmu-miR-503 (↑)	*Ak3l1*	-1.73	-9.2	0.002	10506269
mmu-miR-503* (↑)	*Bcl2*	-2.88	-20.7	4.00E-04	10357043
	Cbx5	-4.33	-12.9	8.00E-04	10433088
	Ccnd2	-2.71	-11.6	0.001	10548105
	Chd9	-2.87	-16.7	5.00E-04	10573823
	Chek1	-4.34	-17.9	5.00E-04	10592201
	Eif4e	-2.32	-12.4	9.00E-04	10496485
	Hnrnpa1	-1.87	-8.4	0.003	10427885
	Kdsr	-2.12	-13	8.00E-04	10357051
	Myb	-3.49	-19	4.00E-04	10368199
	Ocrl	-3.21	-19.7	4.00E-04	10599435
	Tmem93	-2.03	-8.9	0.002	10388238
	Ubfd1	-2.79	-16	5.00E-04	10557139
	Wee1	-5	-16.4	5.00E-04	10556266
	Zbtb41	-2.2	-12	0.001	10350377
	Zfp367	-2.71	-11.8	0.001	10410092
mmu-miR-150 (↑)	*4933439F18Rik*	-3.33	-20.2	4.00E-04	10376596
	Eif4e	-2.32	-12.4	9.00E-04	10496485
	Fbxo21	-2.23	-13.4	8.00E-04	10524941

	Myb	-3.49	-19	4.00E-04	10368199
	Pdia3	-2.15	-11.6	0.001	10475335
	Pdia6	-3.72	-27.5	3.00E-04	10394735
	Pold3	-2.48	-9.1	0.002	10565862
	Sephs1	-2.67	-13.5	8.00E-04	10469035
mmu-miR-340-5p (↑)	*4933403F05Rik*	-2.74	-18.5	4.00E-04	10459604
	Adrbk2	-2.12	-8.5	0.003	10532630
	Amd1	-2.46	-7.3	0.004	10538932
	Angpt1	-5	-11	0.001	10428376
	Ankrd10	-3.19	-23.2	3.00E-04	10577048
	Anp32e	-3.84	-14.7	6.00E-04	10494322
	Asf1a	-2.82	-20.5	4.00E-04	10363163
	Canx	-2.83	-12.9	8.00E-04	10385599
	Ccdc50	-2.76	-11.1	0.001	10434869
	Ccdc88a	-2.57	-14.1	7.00E-04	10374842
	Creld2	-2.34	-11.3	0.001	10426098
	Dio2	-2.78	-20.4	4.00E-04	10401841
	Dnajc11	-2.63	-18.6	4.00E-04	10510604
	Dnajc2	-2.11	-11	0.001	10528340
	Dusp2	-2.1	-11.7	0.001	10475782
	Eif3j	-2.69	-15.3	6.00E-04	10475405
	Enoph1	-2.35	-17.3	5.00E-04	10523518
	Fbxo21	-2.23	-13.4	8.00E-04	10524941
	Gemin5	-2.45	-12.9	8.00E-04	10386125
	Glce	-2.4	-12.7	9.00E-04	10594289
	Golt1b	-3.09	-21.1	4.00E-04	10542650
	Grb10	-2.17	-13.6	7.00E-04	10384398
	Gspt1	-3.41	-13.8	7.00E-04	10437748
	Heatr3	-3.47	-18.7	4.00E-04	10573713
	Hs2st1	-2.48	-7.5	0.004	10502522
	Hspe1	-3.77	-18.1	5.00E-04	10455588
	Ide	-5.16	-19.1	4.00E-04	10467230
	Ikzf2	-2.23	-12	0.001	10355312
	Kdelc1	-4.29	-16	5.00E-04	10354286
	Kit	-2.77	-9.3	0.002	10522530
	Lphn2	-3.25	-14.7	6.00E-04	10502780
	Mat2a	-3.23	-17.4	5.00E-04	10545417
	Memo1	-2.96	-17.3	5.00E-04	10452860
	Neto2	-2.84	-12.1	0.001	10580382
	Npat	-2.68	-12.7	9.00E-04	10585358
	Pdik1l	-2.12	-15.5	6.00E-04	10517243
	Pds5b	-2.68	-14.9	6.00E-04	10527832
	Phf17	-2.36	-16.3	5.00E-04	10491860
	Plk1	-2.86	-8.8	0.002	10557156
	Ptbp1	-3.3	-20.9	4.00E-04	10364518
	Qser1	-4.99	-36.7	3.00E-04	10485622
	Ranbp1	-4.64	-25.3	3.00E-04	10438308
	Rif1	-4.16	-27.1	3.00E-04	10472058
	Scoc	-2.67	-16.7	5.00E-04	10450904
	Sephs1	-2.67	-13.5	8.00E-04	10469035
	Serbp1	-2.63	-11.5	0.001	10538857
	Skp2	-2.97	-21.9	4.00E-04	10427606
	Slc43a1	-2.47	-9.7	0.002	10473367
	Smarcad1	-3.33	-15	6.00E-04	10538755
	Srm	-5.13	-23.5	3.00E-04	10510391
	Ssbp2	-2.2	-8.1	0.003	10406551
	Syncrip	-2.82	-16.3	5.00E-04	10595604
	Tcp1	-4.01	-20.9	4.00E-04	10441797
	Thoc1	-3.3	-16.5	5.00E-04	10453766
	Tomm40	-2.66	-14.6	6.00E-04	10560630
	Ube2v2	-2.02	-10.5	0.001	10437942
	Utp15	-3.05	-18	5.00E-04	10411432
	Xpot	-3.65	-16.1	5.00E-04	10372877
	Zfp664	-2.14	-15	6.00E-04	10525877
mmu-miR-16 (↑)	*1300001I01Rik*	-3.87	-21.5	4.00E-04	10378453
mmu-miR-16* (↑)	*Ak3l1*	-1.73	-9.2	0.002	10506269
	Akt3	-2.59	-15	6.00E-04	10360506
	Atp5g1	-2.81	-16.4	5.00E-04	10391100
	Bace1	-2.14	-15.4	6.00E-04	10584941
	Bcl2	-2.88	-20.7	4.00E-04	10357043
	Cbx5	-4.33	-12.9	8.00E-04	10433088
	Ccnd2	-2.71	-11.6	0.001	10548105
	Ccne1	-2.12	-12.3	9.00E-04	10562563
	Cdc25a	-2.08	-8.4	0.003	10589420
	Chd9	-2.87	-16.7	5.00E-04	10573823
	Chek1	-4.34	-17.9	5.00E-04	10592201
	Cops7a	-2.31	-12.9	8.00E-04	10547926
	Eif2s1	-3.46	-19.2	4.00E-04	10396795
	Eif4e	-2.32	-12.4	9.00E-04	10496485
	Epha7	-2.82	-13.3	8.00E-04	10503659
	Fbxo21	-2.23	-13.4	8.00E-04	10524941
	Fkbp1a	-2.11	-13.2	8.00E-04	10477012
	Gga2	-2.73	-18.5	4.00E-04	10567626
	Gpn1	-3.05	-15.9	5.00E-04	10520800
	Hdgf	-3.2	-20.2	4.00E-04	10493086
	Hmga1	-2.84	-14.9	6.00E-04	10383479
	Hnrnpa1	-1.87	-8.4	0.003	10427885
	Isoc1	-2.92	-16.4	5.00E-04	10455912
	Kdsr	-2.12	-13	8.00E-04	10357051
	Lphn2	-3.25	-14.7	6.00E-04	10502780
	Myb	-3.49	-19	4.00E-04	10368199
	Nup210	-3.84	-22.5	3.00E-04	10546294
	Ocrl	-3.21	-19.7	4.00E-04	10599435
	Otud4	-2.03	-14.2	7.00E-04	10573027
	Pdcd4	-2.55	-16.7	5.00E-04	10463997
	Pdia6	-3.72	-27.5	3.00E-04	10394735
	Pdik1l	-2.12	-15.5	6.00E-04	10517243
	Ptpn4	-3.07	-22	4.00E-04	10357191
	Rad23b	-2.05	-12.3	9.00E-04	10505092
	Scoc	-2.67	-16.7	5.00E-04	10450904
	Seh1l	-2.29	-15.8	5.00E-04	10456423
	Serbp1	-2.63	-11.5	0.001	10538857
	Shcbp1	-3.79	-9.2	0.002	10576883
	Sil1	-2.54	-12.7	9.00E-04	10458251
	Slc39a10	-3.88	-20.9	4.00E-04	10354389
	Smad5	-2.46	-9.7	0.002	10405605
	Sms	-4.2	-23.4	3.00E-04	10607524
	Smyd5	-3.2	-17.5	5.00E-04	10539592
	Sumo3	-2.82	-18.4	4.00E-04	10364287
	Supt16h	-2.01	-12.4	9.00E-04	10419611
	Tmem206	-2.05	-15.1	6.00E-04	10352725
	Ubfd1	-2.79	-16	5.00E-04	10557139
	Usp14	-3.74	-23.5	3.00E-04	10457409
	Vat1	-2.44	-10.4	0.001	10391454
	Wee1	-5	-16.4	5.00E-04	10556266
	Zbtb41	-2.2	-12	0.001	10350377
	Zfp326	-2.52	-13.5	8.00E-04	10523785
	Zfp367	-2.71	-11.8	0.001	10410092
mmu-miR-195 (↑)	*1300001I01Rik*	-3.87	-21.5	4.00E-04	10378453
	Ak3l1	-1.73	-9.2	0.002	10506269
	Akt3	-2.59	-15	6.00E-04	10360506
	Atp5g1	-2.81	-16.4	5.00E-04	10391100
	Bace1	-2.14	-15.4	6.00E-04	10584941
	Bcl2	-2.88	-20.7	4.00E-04	10357043
	Cbx5	-4.33	-12.9	8.00E-04	10433088
	Ccnd2	-2.71	-11.6	0.001	10548105
	Ccne1	-2.12	-12.3	9.00E-04	10562563
	Cdc25a	-2.08	-8.4	0.003	10589420
	Chd9	-2.87	-16.7	5.00E-04	10573823
	Chek1	-4.34	-17.9	5.00E-04	10592201

	Cops7a	-2.31	-12.9	8.00E-04	10547926
	Eif2s1	-3.46	-19.2	4.00E-04	10396795
	Eif4e	-2.32	-12.4	9.00E-04	10496485
	Epha7	-2.82	-13.3	8.00E-04	10503659
	Fbxo21	-2.23	-13.4	8.00E-04	10524941
	Fkbp1a	-2.11	-13.2	8.00E-04	10477012
	Gga2	-2.73	-18.5	4.00E-04	10567626
	Gpn1	-3.05	-15.9	5.00E-04	10520800
	Hdgf	-3.2	-20.2	4.00E-04	10493086
	Hmga1	-2.84	-14.9	6.00E-04	10383479
	Hnrnpa1	-1.87	-8.4	0.003	10427885
	Isoc1	-2.92	-16.4	5.00E-04	10455912
	Kdsr	-2.12	-13	8.00E-04	10357051
	Lphn2	-3.25	-14.7	6.00E-04	10502780
	Myb	-3.49	-19	4.00E-04	10368199
	Nup210	-3.84	-22.5	3.00E-04	10546294
	Ocrl	-3.21	-19.7	4.00E-04	10599435
	Otud4	-2.03	-14.2	7.00E-04	10573027
	Pdcd4	-2.55	-16.7	5.00E-04	10463997
	Pdia6	-3.72	-27.5	3.00E-04	10394735
	Pdik1l	-2.12	-15.5	6.00E-04	10517243
	Ptpn4	-3.07	-22	4.00E-04	10357191
	Rad23b	-2.05	-12.3	9.00E-04	10505092
	Scoc	-2.67	-16.7	5.00E-04	10450904
	Seh1l	-2.29	-15.8	5.00E-04	10456423
	Serbp1	-2.63	-11.5	0.001	10538857
	Shcbp1	-3.79	-9.2	0.002	10576883
	Sil1	-2.54	-12.7	9.00E-04	10458251
	Slc39a10	-3.88	-20.9	4.00E-04	10354389
	Smad5	-2.46	-9.7	0.002	10405605
	Sms	-4.2	-23.4	3.00E-04	10607524
	Smyd5	-3.2	-17.5	5.00E-04	10539592
	Sumo3	-2.82	-18.4	4.00E-04	10364287
	Supt16h	-2.01	-12.4	9.00E-04	10419611
	Tmem206	-2.05	-15.1	6.00E-04	10352725
	Ubfd1	-2.79	-16	5.00E-04	10557139
	Usp14	-3.74	-23.5	3.00E-04	10457409
	Vat1	-2.44	-10.4	0.001	10391454
	Wee1	-5	-16.4	5.00E-04	10556266
	Zbtb41	-2.2	-12	0.001	10350377
	Zfp326	-2.52	-13.5	8.00E-04	10523785
	Zfp367	-2.71	-11.8	0.001	10410092
mmu-miR-30a (↑)	*Abi2*	-2.53	-15.4	6.00E-04	10346764
mmu-miR-30b (↑)	*Atp2a2*	-3.07	-14.4	7.00E-04	10533483
mmu-miR-30c (↑)	*B230208H17Rik*	-2.39	-15.5	6.00E-04	10480813
mmu-miR-30d (↑)	*B3gnt5*	-3.35	-21.5	4.00E-04	10434291
mmu-miR-30e (↑)	*Bcl2*	-2.88	-20.7	4.00E-04	10357043
mmu-miR-30e* (↑)	*Bcor*	-2.1	-12	0.001	10603627
	Calu	-3.7	-18.1	5.00E-04	10536818
	Cand1	-2.37	-16.9	5.00E-04	10372750
	Ccne2	-2.7	-6.7	0.006	10503264
	Ccnf	-2.88	-9.2	0.002	10448506
	Ddx19b	-2.21	-10.5	0.001	10581737
	Dio2	-2.78	-20.4	4.00E-04	10401841
	Dock7	-2.62	-13.6	7.00E-04	10514590
	Dpy19l1	-3.78	-20.8	4.00E-04	10591816
	Dpysl2	-2.47	-16.7	5.00E-04	10420988
	Efr3a	-2.13	-14.3	7.00E-04	10424439
	Elovl5	-2.94	-17.9	5.00E-04	10587284
	Eml4	-2.12	-15	6.00E-04	10447141
	Etaa1	-3.18	-23.1	3.00E-04	10384486
	Fyn	-2.13	-11.3	0.001	10362596
	Gatm	-4.45	-19.5	4.00E-04	10487011
	Gclc	-3.34	-23.8	3.00E-04	10587266
	Glce	-2.4	-12.7	9.00E-04	10594289
	Grb10	-2.17	-13.6	7.00E-04	10384398
	Ide	-5.16	-19.1	4.00E-04	10467230
	Ikzf2	-2.23	-12	0.001	10355312
	Isg20l2	-2.86	-17.2	5.00E-04	10493103
	Ivns1abp	-2.98	-21.6	4.00E-04	10350594
	Larp4	-2.02	-13	8.00E-04	10426827
	Mat2a	-3.23	-17.4	5.00E-04	10545417
	Mboat1	-2.73	-17.2	5.00E-04	10404359
	Msi2	-2.13	-13	8.00E-04	10389680
	Mta1	-3.12	-14.6	6.00E-04	10398972
	Mthfd1l	-3.63	-18.9	4.00E-04	10367641
	Mybl2	-3.02	-11.7	0.001	10478355
	Ogfod1	-2.01	-13.6	7.00E-04	10573998
	Orc2l	-4.15	-17.6	5.00E-04	10354845
	P4ha1	-2.05	-9.8	0.002	10363350
	Pdgfrb	-2.01	-6.2	0.008	10456046
	Pdss1	-2.3	-16.6	5.00E-04	10469712
	Phtf2	-2.46	-13.9	7.00E-04	10528238
	Polr3e	-3.75	-19.6	4.00E-04	10557035
	Polr3g	-2.06	-9	0.002	10410877
	Ppid	-2.81	-11.8	0.001	10492671
	Ppp2r1b	-2.14	-13.2	8.00E-04	10585249
	Psmd7	-3.37	-13.5	8.00E-04	10581560
	Rab38	-3.77	-14.3	7.00E-04	10554800
	Rad23b	-2.05	-12.3	9.00E-04	10505092
	Rnmt	-2.83	-15.8	5.00E-04	10456501
	Rqcd1	-2.57	-15.2	6.00E-04	10347386
	Rwdd4a	-2.46	-16.8	5.00E-04	10571728
	Scyl3	-3.24	-14.4	7.00E-04	10359648
	Sdad1	-4.51	-17.8	5.00E-04	10531383
	Sec61a2	-2.12	-9.4	0.002	10479887
	Sfrs7	-2.54	-11.5	0.001	10453102
	Sgcb	-2.38	-10.1	0.002	10530633
	Skp2	-2.97	-21.9	4.00E-04	10427606
	Slc7a6	-3.96	-19.8	4.00E-04	10574985
	Snx30	-2.4	-11.4	0.001	10505224
	Spcs3	-2.72	-19.9	4.00E-04	10578703
	Ssbp2	-2.2	-8.1	0.003	10406551
	Tusc3	-2.53	-15.4	6.00E-04	10571371
	Ube2v2	-2.02	-10.5	0.001	10437942
	Usp14	-3.74	-23.5	3.00E-04	10457409
	Vat1	-2.44	-10.4	0.001	10391454
	Zbtb41	-2.2	-12	0.001	10350377
	Zdhhc21	-4.06	-24.9	3.00E-04	10514072
	Zfp706	-2.42	-11	0.001	10349049
mmu-miR-29a (↑)	*4933439F18Rik*	-3.33	-20.2	4.00E-04	10376596
mmu-miR-29c (↑)	*Abce1*	-4.57	-19.9	4.00E-04	10579874
	Akt3	-2.59	-15	6.00E-04	10360506
	Ankrd49	-2.66	-16.8	5.00E-04	10590968
	Atp5g1	-2.81	-16.4	5.00E-04	10391100
	Blmh	-2.46	-16.7	5.00E-04	10378802
	Cbx5	-4.33	-12.9	8.00E-04	10433088
	Ccdc88a	-2.57	-14.1	7.00E-04	10374842
	Ccnd2	-2.71	-11.6	0.001	10548105
	Cep97	-3.23	-11.4	0.001	10439960
	Csda	-4.22	-29.9	3.00E-04	10548585
	Ctnnd1	-3.11	-16	5.00E-04	10484402
	Dbt	-2.29	-15.4	6.00E-04	10495549
	Dio2	-2.78	-20.4	4.00E-04	10401841
	Dpysl2	-2.47	-16.7	5.00E-04	10420988
	Dusp2	-2.1	-11.7	0.001	10475782
	Eif3j	-2.69	-15.3	6.00E-04	10475405
	Eml4	-2.12	-15	6.00E-04	10447141
	Fem1b	-2.39	-15.1	6.00E-04	10594315
	Impdh1	-2.36	-9.2	0.002	10543572

	Itgb1	-2.14	-9.4	0.002	10576661
	Kbtbd8	-2.03	-10.2	0.002	10540227
	Kdelc1	-4.29	-16	5.00E-04	10354286
	Larp4	-2.02	-13	8.00E-04	10426827
	Map2k6	-2.39	-14.2	7.00E-04	10382300
	Metap2	-2.38	-16	5.00E-04	10371987
	Mybl2	-3.02	-11.7	0.001	10478355
	Nasp	-3.46	-19.5	4.00E-04	10515337
	Nkrf	-3.22	-18.5	4.00E-04	10604053
	Nup160	-2.78	-19	4.00E-04	10473650
	Otud4	-2.03	-14.2	7.00E-04	10573027
	Oxct1	-3.22	-17.3	5.00E-04	10422608
	Pdgfrb	-2.01	-6.2	0.008	10456046
	Pdhx	-2.35	-12.7	9.00E-04	10485429
	Pdik1l	-2.12	-15.5	6.00E-04	10517243
	Ppic	-2.59	-6.8	0.006	10458906
	Sestd1	-2.95	-18	5.00E-04	10484227
	Slc16a1	-4.21	-25.4	3.00E-04	10495035
	Slc7a1	-2.31	-13.8	7.00E-04	10535852
	Sms	-4.2	-23.4	3.00E-04	10607524
	Syncrip	-2.82	-16.3	5.00E-04	10595604
	Tcfec	-4.64	-22.9	3.00E-04	10543239
	Thoc1	-3.3	-16.5	5.00E-04	10453766
	Trim37	-3.62	-23.1	3.00E-04	10379998
	Tspan4	-3.17	-17.4	5.00E-04	10558961
	Ubfd1	-2.79	-16	5.00E-04	10557139
	Xpnpep1	-2.37	-10	0.002	10468489
	Zbtb41	-2.2	-12	0.001	10350377
	Zdhhc21	-4.06	-24.9	3.00E-04	10514072
	Zfp113	-2.1	-10.5	0.001	10534966
mmu-miR-140 (↑)	*Calu*	-3.7	-18.1	5.00E-04	10536818
	Dpysl2	-2.47	-16.7	5.00E-04	10420988
	Hs2st1	-2.48	-7.5	0.004	10502522
	Rad54l2	-2.21	-7.6	0.004	10596543
	Slc30a5	-2.79	-15.8	5.00E-04	10411751
mmu-miR-142-3p (↑)	*BC016423*	-2.86	-20.1	4.00E-04	10407392
	Eml4	-2.12	-15	6.00E-04	10447141
	Foxm1	-2.02	-7.4	0.004	10542079
	Gas2l3	-3.03	-12	0.001	10371770
	Gfi1	-2.03	-14.9	6.00E-04	10532124
	Il6st	-3.97	-9.8	0.002	10407173
	Lrrc59	-2.68	-17.2	5.00E-04	10380403
	Msi2	-2.13	-13	8.00E-04	10389680
	S1pr3	-2.43	-14.1	7.00E-04	10405179
	Slc39a10	-3.88	-20.9	4.00E-04	10354389
	Usp6nl	-2.57	-18.6	4.00E-04	10469110
	Zbtb41	-2.2	-12	0.001	10350377
mmu-miR-103 (↑)	*Ak3l1*	-1.73	-9.2	0.002	10506269
	Cdc6	-3.33	-20.9	4.00E-04	10381072
	Cdk6	-4.23	-29.9	3.00E-04	10519324
	Ctnnd1	-3.11	-16	5.00E-04	10484402
	Elovl6	-3	-11.1	0.001	10495993
	Fubp1	-2.95	-19.6	4.00E-04	10496892
	Gnpnat1	-3.55	-16.5	5.00E-04	10419216
	Hmga1	-2.84	-14.9	6.00E-04	10383479
	Msi2	-2.13	-13	8.00E-04	10389680
	Otud4	-2.03	-14.2	7.00E-04	10573027
	Poldip2	-2.38	-11.6	0.001	10379204
	Ptpn4	-3.07	-22	4.00E-04	10357191
	Ran	-3.55	-25.7	3.00E-04	10525983
	Rnmt	-2.83	-15.8	5.00E-04	10456501
	Sms	-4.2	-23.4	3.00E-04	10607524
	Tmem206	-2.05	-15.1	6.00E-04	10352725
	Usp14	-3.74	-23.5	3.00E-04	10457409
	Zc3h7b	-2.12	-14.8	6.00E-04	10425578
	Zfp326	-2.52	-13.5	8.00E-04	10523785
mmu-miR-192 (↑)	*Cdc6*	-3.33	-20.9	4.00E-04	10381072
	Dbt	-2.29	-15.4	6.00E-04	10495549
mmu-miR-24 (↑)	*Aars*	-2.48	-12.6	9.00E-04	10575550
	B3gnt5	-3.35	-21.5	4.00E-04	10434291
	Cbx5	-4.33	-12.9	8.00E-04	10433088
	Ccdc58	-2.64	-10.9	0.001	10435489
	Cct3	-4.78	-14	7.00E-04	10493193
	Eml4	-2.12	-15	6.00E-04	10447141
	Gria3	-6.15	-27.8	3.00E-04	10599348
	Gtpbp10	-2.27	-12.3	9.00E-04	10578069
	H2afx	-3.15	-15.9	5.00E-04	10584710
	Immt	-2.88	-20.9	4.00E-04	10539026
	Nfxl1	-2.05	-14.9	6.00E-04	10530467
	Npat	-2.68	-12.7	9.00E-04	10585358
	Sdad1	-4.51	-17.8	5.00E-04	10531383
	Skp2	-2.97	-21.9	4.00E-04	10427606
	Spcs3	-2.72	-19.9	4.00E-04	10578703
	Tcerg1	-3.7	-18	5.00E-04	10455346
	Whsc1	-2.55	-18.6	4.00E-04	10521136
mmu-miR-301a (↑)<br>mmu-miR-301b (↑)	*1300001I01Rik*	-3.87	-21.5	4.00E-04	10378453
	Ak3l1	-3.01	-13.6	7.00E-04	10472923
	Canx	-2.83	-12.9	8.00E-04	10385599
	Cd69	-2.82	-9.6	0.002	10548333
	Cds1	-2.01	-6.1	0.008	10523563
	Cnih	-2.92	-13.6	7.00E-04	10419267
	Dnm1l	-2.23	-15.4	6.00E-04	10437992
	Dpysl2	-2.47	-16.7	5.00E-04	10420988
	Dynll2	-2.07	-8.1	0.003	10389674
	Endod1	-2.34	-15.1	6.00E-04	10590909
	Fubp1	-2.95	-19.6	4.00E-04	10496892
	Gnpnat1	-3.55	-16.5	5.00E-04	10419216
	Grb10	-2.17	-13.6	7.00E-04	10384398
	Il6st	-3.97	-9.8	0.002	10407173
	Impdh1	-2.36	-9.2	0.002	10543572
	Itpr1	-3.21	-19.8	4.00E-04	10540408
	Kit	-2.77	-9.3	0.002	10522530
	Memo1	-2.96	-17.3	5.00E-04	10452860
	Myb	-3.49	-19	4.00E-04	10368199
	Npat	-2.68	-12.7	9.00E-04	10585358
	Nup35	-2.15	-12.8	9.00E-04	10567131
	Pgm2l1	-2.73	-12.2	0.001	10555303
	Phf17	-2.36	-16.3	5.00E-04	10491860
	Qser1	-4.99	-36.7	3.00E-04	10485622
	Rbbp8	-2.37	-12.1	0.001	10453867
	Rpa2	-3.37	-18.2	4.00E-04	10508759
	Rtcd1	-2.28	-14	7.00E-04	10501649
	Sgcb	-2.38	-10.1	0.002	10530633
	Smad5	-2.46	-9.7	0.002	10405605
	Snx5	-2.87	-14.7	6.00E-04	10488237
	Suv39h1	-3.45	-19.6	4.00E-04	10603431
	Syncrip	-2.82	-16.3	5.00E-04	10595604
	Trim37	-3.62	-23.1	3.00E-04	10379998
	Zfp113	-2.1	-10.5	0.001	10534966
	Zfp367	-2.71	-11.8	0.001	10410092
mmu-miR-27a (↑)	*Ak3l1*	-1.73	-9.2	0.002	10506269
	Ccdc50	-2.76	-11.1	0.001	10434869
	Cds1	-2.01	-6.1	0.008	10523563
	Clpp	-3	-16	5.00E-04	10446207
	Dynll2	-2.07	-8.1	0.003	10389674
	E2f6	-3.47	-14.7	6.00E-04	10394690
	Eif2s2	-2.06	-10.9	0.001	10488806
	Fasn	-3.65	-24.7	3.00E-04	10393970
	Fubp1	-2.95	-19.6	4.00E-04	10496892
	Gata2	-2.63	-12.4	9.00E-04	10539873

	Gmps	-3.51	-17.3	5.00E-04	10492381
	Gnpnat1	-3.55	-16.5	5.00E-04	10419216
	Golt1b	-3.09	-21.1	4.00E-04	10542650
	Gpam	-2.11	-13.9	7.00E-04	10468533
	Gria3	-6.15	-27.8	3.00E-04	10599348
	Gspt1	-3.41	-13.8	7.00E-04	10437748
	Gtf2i	-4.11	-23.5	3.00E-04	10534216
	Hsp90aa1	-2.79	-14.9	6.00E-04	10378848
	Hyou1	-3.32	-22.5	3.00E-04	10584712
	Ikzf2	-2.23	-12	0.001	10355312
	Kbtbd8	-2.03	-10.2	0.002	10540227
	Ktelc1	-3.54	-25.6	3.00E-04	10439471
	Larp4	-2.02	-13	8.00E-04	10426827
	Limk1	-2.13	-10.8	0.001	10534324
	Lphn2	-3.25	-14.7	6.00E-04	10502780
	Man1a	-4.55	-27.2	3.00E-04	10369154
	Mdn1	-2.77	-18.6	4.00E-04	10503723
	Met	-4.45	-13.7	7.00E-04	10536505
	Msi2	-2.13	-13	8.00E-04	10389680
	Neto2	-2.84	-12.1	0.001	10580382
	Pank1	-3.63	-21.4	4.00E-04	10467162
	Pdhx	-2.35	-12.7	9.00E-04	10485429
	Pdia5	-2.81	-16.2	5.00E-04	10439218
	Pds5b	-2.68	-14.9	6.00E-04	10527832
	Pgm2l1	-2.73	-12.2	0.001	10555303
	Phb	-2.3	-10.8	0.001	10422776
	Ppif	-2.24	-13.4	8.00E-04	10413222
	Rad54l2	-2.21	-7.6	0.004	10596543
	Rpn1	-2.63	-16.5	5.00E-04	10539861
	Rpn2	-4.13	-29.8	3.00E-04	10477946
	Snx30	-2.4	-11.4	0.001	10505224
	Strbp	-2.39	-10.8	0.001	10482181
	Suclg2	-4.13	-21.8	4.00E-04	10607497
	Tfam	-2.65	-15.8	5.00E-04	10369867
	Uqcc	-2.8	-15.3	6.00E-04	10488944
mmu-miR-328 (↑)	*H2afx*	-3.15	-15.9	5.00E-04	10584710
	Rad23b	-2.05	-12.3	9.00E-04	10505092
	Ubfd1	-2.79	-16	5.00E-04	10557139
	Usp37	-2.69	-18.6	4.00E-04	10355582
mmu-miR-106b (↑)	*4933403F05Rik*	-2.74	-18.5	4.00E-04	10459604
	4933439F18Rik	-3.33	-20.2	4.00E-04	10376596
	6030458C11Rik	-2.78	-18.5	4.00E-04	10427849
	Akt3	-2.59	-15	6.00E-04	10360506
	Asf1a	-2.82	-20.5	4.00E-04	10363163
	Asf1b	-2.99	-9.1	0.002	10573261
	Atad2	-3.16	-17	5.00E-04	10428763
	Brms1l	-2.2	-15	6.00E-04	10395831
	C80913	-3.34	-16.4	5.00E-04	10562548
	Cand1	-2.37	-16.9	5.00E-04	10372750
	Ccdc50	-2.76	-11.1	0.001	10434869
	Ccnd2	-2.71	-11.6	0.001	10548105
	Cd69	-2.82	-9.6	0.002	10548333
	Cdca7	-2.55	-14.4	7.00E-04	10472916
	Cep97	-3.23	-11.4	0.001	10439960
	Chd9	-2.87	-16.7	5.00E-04	10573823
	Clic4	-2.18	-9.1	0.002	10517336
	Dpysl2	-2.47	-16.7	5.00E-04	10420988
	Dusp2	-2.1	-11.7	0.001	10475782
	Ect2	-3.73	-8.9	0.002	10497520
	Elovl6	-3	-11.1	0.001	10495993
	Epha7	-2.82	-13.3	8.00E-04	10503659
	Fbxo21	-2.23	-13.4	8.00E-04	10524941
	Glce	-2.4	-12.7	9.00E-04	10594289
	Hmgb3	-2.34	-10.3	0.002	10600017
	Hs2st1	-2.48	-7.5	0.004	10502522
	Ikzf2	-2.23	-12	0.001	10355312
	Il6st	-3.97	-9.8	0.002	10407173
	Isoc1	-2.92	-16.4	5.00E-04	10455912
	Kpna2	-2.21	-9.6	0.002	10497503
	Limk1	-2.13	-10.8	0.001	10534324
	M6pr	-2.04	-10	0.002	10541484
	Mastl	-2.67	-15.4	6.00E-04	10480432
	Mef2c	-2.16	-12.3	9.00E-04	10406434
	Neto2	-2.84	-12.1	0.001	10580382
	Npat	-2.68	-12.7	9.00E-04	10585358
	Nr2c1	-2.06	-14.1	7.00E-04	10365870
	Nup35	-2.15	-12.8	9.00E-04	10567131
	Ocrl	-3.21	-19.7	4.00E-04	10599435
	Pgm2l1	-2.73	-12.2	0.001	10555303
	Phf6	-2.85	-13.2	8.00E-04	10599612
	Phtf2	-2.46	-13.9	7.00E-04	10528238
	Polq	-2.83	-16.2	5.00E-04	10435581
	Polr3g	-2.06	-9	0.002	10410877
	Ptbp1	-3.3	-20.9	4.00E-04	10364518
	Ptpn4	-3.07	-22	4.00E-04	10357191
	Qser1	-4.99	-36.7	3.00E-04	10485622
	Rad23b	-2.05	-12.3	9.00E-04	10505092
	Rrm2	-3.5	-15.8	5.00E-04	10394978
	Smad5	-2.46	-9.7	0.002	10405605
	Snx30	-2.4	-11.4	0.001	10505224
	Snx5	-2.87	-14.7	6.00E-04	10488237
	Suv39h1	-3.45	-19.6	4.00E-04	10603431
	Tcfap4	-3.2	-17.2	5.00E-04	10437384
	Trim37	-3.62	-23.1	3.00E-04	10379998
	Txlna	-2.01	-14.3	7.00E-04	10516666
	Ubfd1	-2.79	-16	5.00E-04	10557139
	Wee1	-5	-16.4	5.00E-04	10556266
	Zbtb41	-2.2	-12	0.001	10350377
	Zc3h7b	-2.12	-14.8	6.00E-04	10425578
	Zfp367	-2.71	-11.8	0.001	10410092
	Zfp422	-2.56	-17.3	5.00E-04	10443940
mmu-miR-186 (↑)	*2700060E02Rik*	-2.07	-9.7	0.002	10499425
	C80913	-3.34	-16.4	5.00E-04	10562548
	Ccnd2	-2.71	-11.6	0.001	10548105
	Chd9	-2.87	-16.7	5.00E-04	10573823
	Cirh1a	-3.99	-19.1	4.00E-04	10575102
	Dek	-2.92	-10.2	0.002	10409031
	Eif4e	-2.32	-12.4	9.00E-04	10496485
	Ext1	-2.16	-10.9	0.001	10428579
	Fut8	-3.35	-21.1	4.00E-04	10396712
	Gria3	-6.15	-27.8	3.00E-04	10599348
	Hyou1	-3.32	-22.5	3.00E-04	10584712
	Ints2	-3.55	-22.6	3.00E-04	10389421
	Mdn1	-2.77	-18.6	4.00E-04	10503723
	Nasp	-3.46	-19.5	4.00E-04	10515337
	Nek1	-2.32	-13	8.00E-04	10571922
	Nup93	-4.17	-19.9	4.00E-04	10574033
	Otud4	-2.03	-14.2	7.00E-04	10573027
	Pds5b	-2.68	-14.9	6.00E-04	10527832
	Ran	-3.55	-25.7	3.00E-04	10525983
	Snx30	-2.4	-11.4	0.001	10505224
	Xpot	-3.65	-16.1	5.00E-04	10372877
mmu-miR-146a (↑)	*Depdc6*	-2.72	-8	0.003	10424126
	Ivns1abp	-2.98	-21.6	4.00E-04	10350594
	Psmd3	-2.21	-16.2	5.00E-04	10380986
	Strbp	-2.39	-10.8	0.001	10482181
	Taf9b	-2.05	-13.4	8.00E-04	10606315
	Tfdp2	-3.07	-16	5.00E-04	10588007
	Zfp367	-2.71	-11.8	0.001	10410092
mmu-miR-425 (↑)	*Dek*	-2.92	-10.2	0.002	10409031

	Hnrnpab	-2.84	-15	6.00E-04	10385686
	Mef2c	-2.16	-12.3	9.00E-04	10406434
	Rnf219	-2.41	-9.6	0.002	10422185
	Slc16a1	-4.21	-25.4	3.00E-04	10495035
	Syncrip	-2.82	-16.3	5.00E-04	10595604
mmu-miR-7 (↑)	*0610007P14Rik*	-2.57	-15.3	6.00E-04	10401667
	Abi2	-2.53	-15.4	6.00E-04	10346764
	Ankrd49	-2.66	-16.8	5.00E-04	10590968
	Atp2a2	-3.07	-14.4	7.00E-04	10533483
	Bcap29	-4.75	-14.5	6.00E-04	10399874
	Bzw2	-4.67	-26.8	3.00E-04	10400030
	Calu	-3.7	-18.1	5.00E-04	10536818
	Cand1	-2.37	-16.9	5.00E-04	10372750
	Cbx5	-4.33	-12.9	8.00E-04	10433088
	Ccnd2	-2.71	-11.6	0.001	10548105
	Cdc25a	-2.08	-8.4	0.003	10589420
	Clpb	-2.03	-13.9	7.00E-04	10555550
	Cnn3	-3.37	-17.3	5.00E-04	10406852
	Crtap	-3.25	-23.7	3.00E-04	10597413
	Ddost	-4	-24.1	3.00E-04	10509542
	Ddx19a	-2.22	-10.9	0.001	10581729
	Ddx19b	-2.21	-10.5	0.001	10581737
	E2f6	-3.47	-14.7	6.00E-04	10394690
	Eif4e	-2.32	-12.4	9.00E-04	10496485
	Ergic1	-2.43	-12.2	0.001	10443009
	Ezh2	-3.13	-20.5	4.00E-04	10544501
	Gatm	-4.45	-19.5	4.00E-04	10487011
	Gnptab	-2.02	-12.5	9.00E-04	10365601
	Golt1b	-3.09	-21.1	4.00E-04	10542650
	Gspt1	-3.41	-13.8	7.00E-04	10437748
	Gtf2i	-4.11	-23.5	3.00E-04	10534216
	H47	-2.29	-12.1	0.001	10554005
	Hmga1	-2.84	-14.9	6.00E-04	10383479
	Hook1	-3.12	-20.9	4.00E-04	10506004
	Iars	-2.17	-10.7	0.001	10360884
	Ide	-5.16	-19.1	4.00E-04	10467230
	Ikbkap	-2.64	-14.1	7.00E-04	10513020
	Ikzf2	-2.23	-12	0.001	10355312
	Ints2	-3.55	-22.6	3.00E-04	10389421
	Klhl23	-2.08	-13.4	8.00E-04	10472598
	Ktelc1	-3.54	-25.6	3.00E-04	10439471
	Letmd1	-2.05	-14	7.00E-04	10426909
	Lphn2	-3.25	-14.7	6.00E-04	10502780
	Msi2	-2.13	-13	8.00E-04	10389680
	Nap1l1	-3.6	-13.5	8.00E-04	10366337
	Nfxl1	-2.05	-14.9	6.00E-04	10530467
	Nme4	-2.42	-7	0.005	10449236
	Nr2c1	-2.06	-14.1	7.00E-04	10365870
	Parp1	-4.23	-11.3	0.001	10352242
	Pgm2l1	-2.73	-12.2	0.001	10555303
	Phf17	-2.36	-16.3	5.00E-04	10491860
	Pik3ip1	-2.02	-13.1	8.00E-04	10373740
	Pole4	-2.98	-17.1	5.00E-04	10545583
	Ppm1f	-2.22	-13.2	8.00E-04	10433929
	Rrm2	-3.5	-15.8	5.00E-04	10394978
	Scd2	-4.45	-16.7	5.00E-04	10463355
	Scyl3	-3.24	-14.4	7.00E-04	10359648
	Skp2	-2.97	-21.9	4.00E-04	10427606
	Smarcad1	-3.33	-15	6.00E-04	10538755
	Smarcc1	-3.89	-25.2	3.00E-04	10589466
	Smyd5	-3.2	-17.5	5.00E-04	10539592
	Snx30	-2.4	-11.4	0.001	10505224
	Snx5	-2.87	-14.7	6.00E-04	10488237
	Spryd4	-2.62	-18.4	4.00E-04	10373355
	Strbp	-2.39	-10.8	0.001	10482181
	Syncrip	-2.82	-16.3	5.00E-04	10595604
	Taf9b	-2.05	-13.4	8.00E-04	10606315
	Tcf12	-3.29	-18.4	4.00E-04	10594879
	Tfrc	-3.33	-18.9	4.00E-04	10435075
	Thoc1	-3.3	-16.5	5.00E-04	10453766
	Ttll4	-2.36	-10.9	0.001	10347460
	Txlna	-2.01	-14.3	7.00E-04	10516666
mmu-miR-342-3p (↑)	*Ankrd49*	-2.66	-16.8	5.00E-04	10590968
	Cbx5	-4.33	-12.9	8.00E-04	10433088
	Fut8	-3.35	-21.1	4.00E-04	10396712
	Gspt1	-3.41	-13.8	7.00E-04	10437748
	Kdsr	-2.12	-13	8.00E-04	10357051
	Pgm2l1	-2.73	-12.2	0.001	10555303
	Rad23b	-2.05	-12.3	9.00E-04	10505092
	Tfdp2	-3.07	-16	5.00E-04	10588007
mmu-miR-148a (↑)	*1300001I01Rik*	-3.87	-21.5	4.00E-04	10378453
	Arhgap21	-2.49	-10	0.002	10480381
	Atp2a2	-3.07	-14.4	7.00E-04	10533483
	Cand1	-2.37	-16.9	5.00E-04	10372750
	Canx	-2.83	-12.9	8.00E-04	10385599
	Ccnf	-2.88	-9.2	0.002	10448506
	Chchd4	-2.35	-13.3	8.00E-04	10546346
	Chd9	-2.87	-16.7	5.00E-04	10573823
	Cul5	-2.11	-14.6	6.00E-04	10593605
	Dgcr8	-2.31	-14.4	7.00E-04	10438313
	Dnmt1	-2.91	-13.8	7.00E-04	10591369
	Dynll2	-2.07	-8.1	0.003	10389674
	Fubp1	-2.95	-19.6	4.00E-04	10496892
	Gnpnat1	-3.55	-16.5	5.00E-04	10419216
	Il6st	-3.97	-9.8	0.002	10407173
	Met	-4.45	-13.7	7.00E-04	10536505
	Msi2	-2.13	-13	8.00E-04	10389680
	Otud4	-2.03	-14.2	7.00E-04	10573027
	Pdia3	-2.15	-11.6	0.001	10475335
	Pgm2l1	-2.73	-12.2	0.001	10555303
	Sgcb	-2.38	-10.1	0.002	10530633
	Sms	-4.2	-23.4	3.00E-04	10607524
	Syncrip	-2.82	-16.3	5.00E-04	10595604
	Tfrc	-3.33	-18.9	4.00E-04	10435075
	Tomm70a	-2.42	-13.7	7.00E-04	10436348
	Whsc1	-2.55	-18.6	4.00E-04	10521136
mmu-let-7g (↑)	*0610007P14Rik*	-2.57	-15.3	6.00E-04	10401667
	Abi2	-2.53	-15.4	6.00E-04	10346764
	Ankrd49	-2.66	-16.8	5.00E-04	10590968
	Atp2a2	-3.07	-14.4	7.00E-04	10533483
	Bcap29	-4.75	-14.5	6.00E-04	10399874
	Bzw2	-4.67	-26.8	3.00E-04	10400030
	Calu	-3.7	-18.1	5.00E-04	10536818
	Cand1	-2.37	-16.9	5.00E-04	10372750
	Cbx5	-4.33	-12.9	8.00E-04	10433088
	Ccnd2	-2.71	-11.6	0.001	10548105
	Cnn3	-3.37	-17.3	5.00E-04	10406852
	Crtap	-3.25	-23.7	3.00E-04	10597413
	Ddost	-4	-24.1	3.00E-04	10509542
	Ddx19a	-2.22	-10.9	0.001	10581729
	Ddx19b	-2.21	-10.5	0.001	10581737
	E2f6	-3.47	-14.7	6.00E-04	10394690
	Eif4e	-2.32	-12.4	9.00E-04	10496485
	Ergic1	-2.43	-12.2	0.001	10443009
	Ezh2	-3.13	-20.5	4.00E-04	10544501
	Gatm	-4.45	-19.5	4.00E-04	10487011
	Gnptab	-2.02	-12.5	9.00E-04	10365601
	Golt1b	-3.09	-21.1	4.00E-04	10542650
	Gspt1	-3.41	-13.8	7.00E-04	10437748
	Gtf2i	-4.11	-23.5	3.00E-04	10534216

	H47	-2.29	-12.1	0.001	10554005
	Hmga1	-2.84	-14.9	6.00E-04	10383479
	Hook1	-3.12	-20.9	4.00E-04	10506004
	Iars	-2.17	-10.7	0.001	10360884
	Ide	-5.16	-19.1	4.00E-04	10467230
	Ikbkap	-2.64	-14.1	7.00E-04	10513020
	Ikzf2	-2.23	-12	0.001	10355312
	Ints2	-3.55	-22.6	3.00E-04	10389421
	Ktelc1	-3.54	-25.6	3.00E-04	10439471
	Lphn2	-3.25	-14.7	6.00E-04	10502780
	Nap1l1	-3.6	-13.5	8.00E-04	10366337
	Nme4	-2.42	-7	0.005	10449236
	Parp1	-4.23	-11.3	0.001	10352242
	Pgm2l1	-2.73	-12.2	0.001	10555303
	Phf17	-2.36	-16.3	5.00E-04	10491860
	Pik3ip1	-2.02	-13.1	8.00E-04	10373740
	Pole4	-2.98	-17.1	5.00E-04	10545583
	Rrm2	-3.5	-15.8	5.00E-04	10394978
	Scd2	-4.45	-16.7	5.00E-04	10463355
	Scyl3	-3.24	-14.4	7.00E-04	10359648
	Skp2	-2.97	-21.9	4.00E-04	10427606
	Smarcad1	-3.33	-15	6.00E-04	10538755
	Smarcc1	-3.89	-25.2	3.00E-04	10589466
	Smyd5	-3.2	-17.5	5.00E-04	10539592
	Snx30	-2.4	-11.4	0.001	10505224
	Snx5	-2.87	-14.7	6.00E-04	10488237
	Spryd4	-2.62	-18.4	4.00E-04	10373355
	Strbp	-2.39	-10.8	0.001	10482181
	Syncrip	-2.82	-16.3	5.00E-04	10595604
	Tcf12	-3.29	-18.4	4.00E-04	10594879
	Tfrc	-3.33	-18.9	4.00E-04	10435075
	Thoc1	-3.3	-16.5	5.00E-04	10453766
	Tmed9	-1.99	-6.8	0.006	10405488
	Ttll4	-2.36	-10.9	0.001	10347460
	Zfp362	-1.68	-7	0.005	10516520
mmu-miR-149 (↑)	*1300001I01Rik*	-3.87	-21.5	4.00E-04	10378453
	4930572J05Rik	-2.12	-9	0.002	10424667
	Arhgap21	-2.49	-10	0.002	10480381
	Atp2a2	-3.07	-14.4	7.00E-04	10533483
	Cln6	-3.12	-22.6	3.00E-04	10586110
	Ext1	-2.16	-10.9	0.001	10428579
	Gfi1	-2.03	-14.9	6.00E-04	10532124
	Gria3	-6.15	-27.8	3.00E-04	10599348
	Hnrnpa1	-1.87	-8.4	0.003	10427885
	Ilf3	-3.23	-23.8	3.00E-04	10583610
	Ipo9	-2.5	-13.2	8.00E-04	10358064
	Nucks1	-3.98	-15.1	6.00E-04	10349733
	Prps2	-4.18	-25.4	3.00E-04	10607877
	Ubfd1	-2.79	-16	5.00E-04	10557139
	Wrb	-2.69	-16.2	5.00E-04	10437174
	Zfp704	-1.89	-13	8.00E-04	10497222
mmu-miR-22 (↑)	*Akt3*	-2.59	-15	6.00E-04	10360506
	Cand1	-2.37	-16.9	5.00E-04	10372750
	Cdc6	-3.33	-20.9	4.00E-04	10381072
	Chd9	-2.87	-16.7	5.00E-04	10573823
	Elovl6	-3	-11.1	0.001	10495993
	Gatm	-4.45	-19.5	4.00E-04	10487011
	Lgals1	-4.48	-15	6.00E-04	10425161
	Mat2a	-3.23	-17.4	5.00E-04	10545417
	Mtf2	-2.19	-12.6	9.00E-04	10523905
	Ola1	-2.82	-14.5	6.00E-04	10483648
	Pdss1	-2.3	-16.6	5.00E-04	10469712
	Sephs1	-2.67	-13.5	8.00E-04	10469035
	Snx30	-2.4	-11.4	0.001	10505224
	Yars	-2.81	-14	7.00E-04	10508420
	Zfp706	-2.24	-13.1	8.00E-04	10428211
mmu-miR-25 (↑)	*4933439F18Rik*	-3.33	-20.2	4.00E-04	10376596
	Aars	-2.48	-12.6	9.00E-04	10575550
	Atp2a2	-3.07	-14.4	7.00E-04	10533483
	Cand1	-2.37	-16.9	5.00E-04	10372750
	Cd69	-2.82	-9.6	0.002	10548333
	Cdca7l	-4.02	-15.2	6.00E-04	10399178
	Chd9	-2.87	-16.7	5.00E-04	10573823
	Dbt	-2.29	-15.4	6.00E-04	10495549
	Fubp1	-2.95	-19.6	4.00E-04	10496892
	Gata2	-2.63	-12.4	9.00E-04	10539873
	Gria3	-6.15	-27.8	3.00E-04	10599348
	Ikzf2	-2.23	-12	0.001	10355312
	Itpr1	-3.21	-19.8	4.00E-04	10540408
	Nsmaf	-3.59	-14.3	7.00E-04	10511382
	Phf17	-2.36	-16.3	5.00E-04	10491860
	Prc1	-2.86	-13	8.00E-04	10554445
	Qser1	-4.99	-36.7	3.00E-04	10485622
	Rpl15	-3.09	-11.1	0.001	10547638
	Sfxn1	-4.18	-26.3	3.00E-04	10405236
	Snx30	-2.4	-11.4	0.001	10505224
	Spryd4	-2.62	-18.4	4.00E-04	10373355
	Ssbp2	-2.2	-8.1	0.003	10406551
	Tgif1	-2.16	-16	5.00E-04	10452633
mmu-miR-322* (↑)	*4933439F18Rik*	-3.33	-20.2	4.00E-04	10376596
	Aars	-2.48	-12.6	9.00E-04	10575550
	Atp2a2	-3.07	-14.4	7.00E-04	10533483
	Cand1	-2.37	-16.9	5.00E-04	10372750
	Cd69	-2.82	-9.6	0.002	10548333
	Cdca7l	-4.02	-15.2	6.00E-04	10399178
	Chd9	-2.87	-16.7	5.00E-04	10573823
	Dbt	-2.29	-15.4	6.00E-04	10495549
	Fubp1	-2.95	-19.6	4.00E-04	10496892
	Gata2	-2.63	-12.4	9.00E-04	10539873
	Gria3	-6.15	-27.8	3.00E-04	10599348
	Ikzf2	-2.23	-12	0.001	10355312
	Itpr1	-3.21	-19.8	4.00E-04	10540408
	Nsmaf	-3.59	-14.3	7.00E-04	10511382
	Phf17	-2.36	-16.3	5.00E-04	10491860
	Prc1	-2.86	-13	8.00E-04	10554445
	Qser1	-4.99	-36.7	3.00E-04	10485622
	Rpl15	-3.09	-11.1	0.001	10547638
	Sfxn1	-4.18	-26.3	3.00E-04	10405236
	Snx30	-2.4	-11.4	0.001	10505224
	Spryd4	-2.62	-18.4	4.00E-04	10373355
	Ssbp2	-2.2	-8.1	0.003	10406551
	Tgif1	-2.16	-16	5.00E-04	10452633
mmu-miR-33* (↑)	*Cand1*	-2.37	-16.9	5.00E-04	10372750
	Cdk6	-4.23	-29.9	3.00E-04	10519324
	Ctnnd1	-3.11	-16	5.00E-04	10484402
	Eif2s2	-2.06	-10.9	0.001	10488806
	Gria3	-6.15	-27.8	3.00E-04	10599348
	Mrps25	-2.3	-14.7	6.00E-04	10546396
	Npat	-2.68	-12.7	9.00E-04	10585358
	Nutf2	-3.78	-18.5	4.00E-04	10468531
	Strbp	-2.39	-10.8	0.001	10482181
	Tfdp2	-3.07	-16	5.00E-04	10588007
mmu-miR-93 (↑)	*4933439F18Rik*	-3.33	-20.2	4.00E-04	10376596
	Akt3	-2.59	-15	6.00E-04	10360506
	Atad2	-3.16	-17	5.00E-04	10428763
	C80913	-3.34	-16.4	5.00E-04	10562548
	Cand1	-2.37	-16.9	5.00E-04	10372750
	Ccdc50	-2.76	-11.1	0.001	10434869
	Ccnd2	-2.71	-11.6	0.001	10548105
	Cd69	-2.82	-9.6	0.002	10548333

	Cep97	-3.23	-11.4	0.001	10439960
	Chd9	-2.87	-16.7	5.00E-04	10573823
	Dusp2	-2.1	-11.7	0.001	10475782
	Elovl6	-3	-11.1	0.001	10495993
	Epha7	-2.82	-13.3	8.00E-04	10503659
	Fbxo21	-2.23	-13.4	8.00E-04	10524941
	Hmgb3	-2.34	-10.3	0.002	10600017
	Hs2st1	-2.48	-7.5	0.004	10502522
	Il6st	-3.97	-9.8	0.002	10407173
	Isoc1	-2.92	-16.4	5.00E-04	10455912
	Kpna2	-2.21	-9.6	0.002	10497503
	Mastl	-2.67	-15.4	6.00E-04	10480432
	Neto2	-2.84	-12.1	0.001	10580382
	Npat	-2.68	-12.7	9.00E-04	10585358
	Nup35	-2.15	-12.8	9.00E-04	10567131
	Ocrl	-3.21	-19.7	4.00E-04	10599435
	Pgm2l1	-2.73	-12.2	0.001	10555303
	Phf6	-2.85	-13.2	8.00E-04	10599612
	Polq	-2.83	-16.2	5.00E-04	10435581
	Ptbp1	-3.3	-20.9	4.00E-04	10364518
	Ptpn4	-3.07	-22	4.00E-04	10357191
	Rrm2	-3.5	-15.8	5.00E-04	10394978
	Smad5	-2.46	-9.7	0.002	10405605
	Trim37	-3.62	-23.1	3.00E-04	10379998
	Ubfd1	-2.79	-16	5.00E-04	10557139
	Wee1	-5	-16.4	5.00E-04	10556266
	Zc3h7b	-2.12	-14.8	6.00E-04	10425578
	Zfp362	-1.68	-7	0.005	10516520
	Zfp367	-2.71	-11.8	0.001	10410092
	Zfp704	-1.89	-13	8.00E-04	10497222
mmu-miR-101a (↓)<br>mmu-miR-101b (↓)	*A930001N09Rik*	2.99	21	4.00E-04	10443027
	Abtb2	4.12	26.7	3.00E-04	10474181
	Arhgef3	2.39	15.2	6.00E-04	10413419
	Baz2b	2.08	14.3	7.00E-04	10482880
	BC017647	2.12	14.9	6.00E-04	10379030
	Dusp1	3	13.7	7.00E-04	10449284
	Ets1	2.82	7.6	0.004	10584142
	Fos	2.8	14.8	6.00E-04	10397346
	Fosl2	2.97	17.1	5.00E-04	10520862
	Itga8	2.59	16.7	5.00E-04	10480090
	Jhdm1d	4.02	24.9	3.00E-04	10544148
	Klf6	2.95	14.6	6.00E-04	10403352
	Lmtk2	2.76	17.3	5.00E-04	10527306
	Map3k8	2.48	10.4	0.001	10457225
	Mxd1	4.86	35.7	3.00E-04	10545921
	Phf20l1	1.83	10.9	0.001	10424485
	Phlda1	3.07	9.9	0.002	10366346
	Ptgs2	5.38	35	3.00E-04	10350516
	Ptprj	2.4	14.9	6.00E-04	10484894
	Purg	2.96	17.4	5.00E-04	10571241
	Rin2	4.31	12	0.001	10476759
	Sgk1	2.66	13.6	7.00E-04	10362073
	Trib1	3.39	23	3.00E-04	10424370
	Trp53inp1	5	25.8	3.00E-04	10503259
	Trp53inp2	3.69	18.9	4.00E-04	10477644
	Zfand3	2.51	15.4	6.00E-04	10443589
	Zfhx3	2.49	14.1	7.00E-04	10575213
mmu-miR-155 (↓)	*Arrb2*	3.18	13.8	7.00E-04	10377804
	Carhsp1	4.3	20.8	4.00E-04	10437590
	Cyb561d1	1.86	12.2	9.00E-04	10501282
	E2f2	2.51	11.7	0.001	10509168
	Ell2	2.58	17	5.00E-04	10406254
	Ets1	2.82	7.6	0.004	10584142
	Fosl2	2.97	17.1	5.00E-04	10520862
	Jhdm1d	4.02	24.9	3.00E-04	10544148
	Myo1d	4.62	15.7	6.00E-04	10389025
	Nfat5	2	12.3	9.00E-04	10575160
	Plekhm1	2	11.5	0.001	10391918
	Rnf149	2.62	16.5	5.00E-04	10354191
	Trp53inp1	5	25.8	3.00E-04	10503259
mmu-miR-196b (↓)	*Cpd*	2.67	18.2	4.00E-04	10388591
	Lin28	3.22	13.8	7.00E-04	10508907
	Pbx1	3.4	10.8	0.001	10359870
	Rab11fip1	3.49	24.6	3.00E-04	10577954
	Rassf3	2.67	16.9	5.00E-04	10372844
mmu-miR-20a (↓)<br>mmu-miR-20a* (↓)<br>mmu-miR-20b (↓)	*4933426M11Rik*	2.05	12.1	0.001	10396919
	Abhd5	2.27	11.7	0.001	10590452
	Arhgef3	2.39	15.2	6.00E-04	10413419
	Atg7	2.48	15.4	6.00E-04	10540860
	E2f2	2.51	11.7	0.001	10509168
	Fcho2	2.75	15.5	6.00E-04	10411464
	Fgd4	2.47	11.7	0.001	10438017
	Foxj2	2.07	11.4	0.001	10541532
	Furin	2.05	12.3	9.00E-04	10564960
	Gpr137b	3.94	17.3	5.00E-04	10407803
	Has2	2.41	6.3	0.007	10428707
	Litaf	3	12.8	8.00E-04	10437687
	Map3k5	2.06	11.7	0.001	10361926
	Map3k8	2.48	10.4	0.001	10457225
	Mcl1	2.1	15.3	6.00E-04	10494306
	Mgll	4.66	29.7	3.00E-04	10539894
	Myo1d	4.62	15.7	6.00E-04	10389025
	Nfat5	2	12.3	9.00E-04	10575160
	Obfc2a	3.95	27.5	3.00E-04	10354418
	Osm	4.33	23.8	3.00E-04	10373912
	Phf1	3.27	17	5.00E-04	10443063
	Plekhm1	2	11.5	0.001	10391918
	Plekho2	2.43	16.8	5.00E-04	10594540
	Ppp1r3b	2.19	12.9	8.00E-04	10571321
	Pten	1.73	12.4	9.00E-04	10462521
	Ptprj	2.4	14.9	6.00E-04	10484894
	Rab11fip1	3.49	24.6	3.00E-04	10577954
	Rab11fip4	2.53	16.9	5.00E-04	10379321
	Rab5b	1.75	12.3	9.00E-04	10486057
	Rasa2	2.18	13.5	8.00E-04	10595805
	Reep3	2.4	12.2	0.001	10369761
	Rell1	2.39	17.1	5.00E-04	10530130
	Rhoc	3.5	22.9	3.00E-04	10495054
	Rnf13	2.25	16.1	5.00E-04	10492180
	Rragd	2.14	11.2	0.001	10503835
	Sema7a	3.47	14.4	7.00E-04	10585778
	Sh3bp5	3.38	16.4	5.00E-04	10418702
	Skil	2.64	16.5	5.00E-04	10491300
	Slc40a1	4.02	27.2	3.00E-04	10354374
	Snrk	2.81	15.7	6.00E-04	10590445
	Sntb2	2.11	14.7	6.00E-04	10575120
	Sorl1	3.44	18.2	4.00E-04	10592535
	Stk38	2.39	15.9	5.00E-04	10449527
	Timp2	4.87	20.1	4.00E-04	10393559
	Trp53inp1	5	25.8	3.00E-04	10503259
	Txnip	2.34	15	6.00E-04	10494428
	Usp32	3.47	24.3	3.00E-04	10389339
	Usp46	1.92	13	8.00E-04	10530641
	Vasp	2.89	19.2	4.00E-04	10560459
	Zhx2	2.6	15.6	6.00E-04	10424213
mmu-miR-320 (↓)	*4933426M11Rik*	2.05	12.1	0.001	10396919
	Aim1	3.58	12	0.001	10368947
	Arpc5	1.95	13.7	7.00E-04	10350684
	Atp11a	2.4	14.5	6.00E-04	10570201
	Bmpr1a	4.81	23	3.00E-04	10418927

	Btg2	3.3	23.9	3.00E-04	10357875
	Cpd	2.67	18.2	4.00E-04	10388591
	Ell2	2.58	17	5.00E-04	10406254
	Emilin2	2.19	9.2	0.002	10452648
	Ets2	2.95	14	7.00E-04	10437160
	Jhdm1d	4.02	24.9	3.00E-04	10544148
	Pbx1	3.4	10.8	0.001	10359870
	Pdha1	3.57	23.7	3.00E-04	10602865
	Phf1	3.27	17	5.00E-04	10443063
	Plk3	4.95	34.7	3.00E-04	10515399
	Ppp1r3b	2.19	12.9	8.00E-04	10571321
	Pten	1.73	12.4	9.00E-04	10462521
	Slc6a6	2.18	13.4	8.00E-04	10540122
	Smap2	2.23	16.5	5.00E-04	10515994
	Zfp516	2.4	13.1	8.00E-04	10457040
mmu-miR-378 (↓)	*Irg1*	4.9	34.3	3.00E-04	10416837
	Kbtbd7	2.85	13.6	7.00E-04	10416653
mmu-miR-494 (↓)	*Arhgef12*	2.66	10.1	0.002	10582862
	Atp7a	2.99	20	4.00E-04	10601360
	Foxj2	2.07	11.4	0.001	10541532
	Gca	2.61	11.3	0.001	10472350
	Kbtbd7	2.85	13.6	7.00E-04	10416653
	Nadk	2.22	15.3	6.00E-04	10511084
	Nfat5	2	12.3	9.00E-04	10575160
	Pten	1.73	12.4	9.00E-04	10462521
	Rnd1	2.5	16.6	5.00E-04	10432236
	Tbc1d8	2.7	17.7	5.00E-04	10354168
	Zfhx3	2.49	14.1	7.00E-04	10575213
mmu-mir-18 (↓)	*Add3*	2.25	13.9	7.00E-04	10463911
	Fosl2	2.97	17.1	5.00E-04	10520862
	Irf2	2.18	11.9	0.001	10571705
	Lin28	3.22	13.8	7.00E-04	10508907
	Nedd9	3.19	21.5	4.00E-04	10408850
	Nfat5	2	12.3	9.00E-04	10575160
	Notch2	2.26	12.7	9.00E-04	10494595
	Olfml2b	4.24	15.3	6.00E-04	10351491
	Pias3	2.4	16.7	5.00E-04	10494509
	Pten	1.73	12.4	9.00E-04	10462521
	Smap2	2.23	16.5	5.00E-04	10515994
	Ypel5	2.18	13.7	7.00E-04	10446756
