# Supplementary material for: Identification of nuclear-enriched miRNAs during mouse granulopoiesis
Source: J Hematol Oncol. 2014 May 15;7:42. doi: 10.1186/1756-8722-7-42 (PMC4046156; doi:10.1186/1756-8722-7-42)
Supplement: Additional file 4 — Differentially expressed miRNAs in LSK compared to promyelocytes that have predicted targets showing inversely correlated expression. [file 1756-8722-7-42-S4.pdf]

Additional file 4- Differentially expressed miRNAs in LSK compared to promyelocytes that have predicted targets showing inversely correlated expression

MicroRNA name (LSK/Promyelocytes expression)	Target mRNAs (LSK/ Promyelocytes expression)
mmu-let-7a (↓)	*Hlf* (↑)<br>*Klf12* (↑)<br>*Mycn* (↑)
mmu-let-7d (↓)	*Hlf* (↑)<br>*Klf12* (↑)<br>*Mycn* (↑)
mmu-let-7g (↓)	*Hlf* (↑)<br>*Klf12* (↑)<br>*Mycn* (↑)
mmu-let-7i (↓)	*Hlf* (↑)<br>*Klf12* (↑)<br>*Mycn* (↑)
mmu-miR-106a (↓)	*Hlf* (↑)<br>*Klf12* (↑)<br>*Mycn* (↑)<br>*Trp53inp1* (↑)<br>*Vldlr* (↑)
mmu-miR-106b* (↓)	*Hlf* (↑)<br>*Klf12* (↑)<br>*Mycn* (↑)<br>*Trp53inp1* (↑)<br>*Vldlr* (↑)
mmu-miR-130b (↓)	*Arhgef12* (↑)<br>*Hlf* (↑)<br>*Trp53inp1* (↑)
mmu-miR-142-3p (↓)	*Arhgef12* (↑)
mmu-miR-148a (↓)	*Arhgef12* (↑)<br>*Hlf* (↑)

mmu-miR-149 (↓)	*Prkg1*(↑)
mmu-miR-15a* (↓)	*Cobll1*(↑)<br>*Eya1*(↑)<br>*Prkg1*(↑)
mmu-miR-15b* (↓)	*Cobll1*(↑)<br>*Eya1*(↑)<br>*Prkg1*(↑)
mmu-miR-17 (↓)	*Hlf* (↑)<br>*Klf12* (↑)<br>*Mycn* (↑)<br>*Trp53inp1* (↑)<br>*Vldlr* (↑)
mmu-miR-195 (↓)	*Cobll1*(↑)<br>*Eya1*(↑)<br>*Prkg1*(↑)
mmu-miR-19a (↓)	*Arhgef12* (↑)<br>*Hlf* (↑)<br>*Klf12* (↑)<br>*Nfatc2*(↑)<br>*Trp53inp1* (↑)
mmu-miR-19b (↓)	*Arhgef12* (↑)<br>*Hlf* (↑)<br>*Klf12* (↑)<br>*Nfatc2*(↑)<br>*Trp53inp1* (↑)
mmu-miR-20a (↓)	*Hlf* (↑)<br>*Klf12* (↑)<br>*Mycn* (↑)<br>*Trp53inp1* (↑)<br>*Vldlr* (↑)
mmu-miR-21 (↓)	*Arhgef12* (↑)
mmu-miR-223 (↓)	*Hlf* (↑)
mmu-miR-27a (↓)	*Eya1*(↑)

	Prkg1(↑)<br>*Rgs1* (↑)<br>*Zfp36* (↑)
mmu-miR-27a* (↓)	*Eya1*(↑)<br>*Prkg1*(↑)<br>*Rgs1* (↑)<br>*Zfp36* (↑)
mmu-miR-27b (↓)	*Eya1*(↑)<br>*Prkg1*(↑)<br>*Rgs1* (↑)<br>*Zfp36* (↑)
mmu-miR-301a (↓)	*Arhgef12* (↑)<br>*Hlf* (↑)<br>*Trp53inp1* (↑)
mmu-miR-301b (↓)	*Arhgef12* (↑)<br>*Hlf* (↑)<br>*Trp53inp1* (↑)
mmu-miR-340-5p (↓)	*Angpt1*(↑)<br>*Nfatc2* (↑)<br>*Xrcc5* (↑)
mmu-miR-494 (↓)	*Arhgef12* (↑)<br>*Hlf* (↑)
mmu-miR-93 (↓)	*Hlf* (↑)<br>*Klf12* (↑)<br>*Mycn* (↑)<br>*Trp53inp1* (↑)<br>*Vldlr* (↑)
mmu-miR-155 (↑)	*Csf1r* (↓)
mmu-miR-203 (↑)	*Met* (↓)
