# Supplementary material for: Identification of nuclear-enriched miRNAs during mouse granulopoiesis
Source: J Hematol Oncol. 2014 May 15;7:42. doi: 10.1186/1756-8722-7-42 (PMC4046156; doi:10.1186/1756-8722-7-42)
Supplement: Additional file 5 — Nuclear to cytoplasmic ratio of mouse miRNAs during granulopoiesis. [file 1756-8722-7-42-S5.pdf]

Additional file 5- Nuclear to cytoplasmic ratio of mouse miRNAs during granulopoiesis

mirName	CT values											
	Cytoplasmic Fraction				Nuclear Fraction				Nuclear:Cytoplasmic Ratio			
	LSK	Promyelocyte	Myelocyte	Granulocyte	LSK	Promyelocyte	Myelocyte	Granulocyte	LSK	Promyelocyte	Myelocyte	Granulocyte
mmu-miR-709	21.049	21.599	20.703	20.432	21.55	21.256	22.225	20.885	0.23553894	0.422797133	0.116067623	0.243507396
mmu-miR-19b	20.3323	21.1878	21.1154	21.2049	24.6411	24.213	25.5764	24.4784	0.01681902	0.040945182	0.015135053	0.034471252
mmu-miR-17	21.0204	21.463	21.7863	21.887	25.1568	24.4985	25.5787	24.2492	0.01895389	0.040653898	0.024057616	0.064831443
mmu-miR-690	24.118	24.052	23.513	22.999	25.352	24.234	25.106	25.023	0.14171236	0.293826719	0.110493813	0.081958506
mmu-miR-191	21.6175	22.83	22.1722	22.0038	25.4596	25.0738	25.4093	24.5245	0.02324296	0.070376496	0.035352045	0.058086129
mmu-miR-106a	21.9894	22.4414	22.5541	22.811	25.9711	25.7164	26.3459	25.6517	0.02109928	0.03443543	0.024067623	0.046531048
mmu-miR-16	22.2538	23.1621	20.6502	20.0423	25.9768	25.798	24.0697	22.5808	0.02524318	0.053628269	0.031153488	0.057373864
mmu-miR-92a	22.3518	23.6428	24.1653	24.3691	26.0503	26.3162	26.8185	26.4409	0.02567552	0.052252269	0.052989029	0.079287514
mmu-miR-222	21.7698	24.3871	25.0038	24.8264	26.0763	27.5318	28.2841	27.5415	0.01684586	0.037690311	0.034309157	0.050763576
mmu-miR-24	22.9452	24.4297	23.2608	22.6351	26.2455	26.8554	26.3492	25.049	0.03383681	0.062039785	0.03919022	0.062549298
mmu-miR-223	21.7887	20.3498	17.5589	16.8299	26.3779	23.7462	22.2864	20.3458	0.01384815	0.031656323	0.012582283	0.029139855
mmu-miR-20a	22.6429	22.8317	22.9198	23.3082	26.7963	26.0355	27.3615	26.307	0.01873185	0.036177524	0.015338886	0.041701338
mmu-miR-142-3p	23.5656	24.116	21.9693	22.1849	27.1846	26.725	25.2847	23.4185	0.02713009	0.054637584	0.033484506	0.141751656
mmu-miR-29a	22.9813	25.0681	23.8708	23.4191	27.2518	28.4008	27.5157	25.6875	0.0172715	0.033085376	0.02664738	0.06918665
mmu-miR-484	23.9541	25.2559	23.132	22.538	27.3194	28.0021	26.9815	25.0846	0.03234614	0.049680982	0.023124043	0.057052642
mmu-miR-19a	22.9558	23.685	23.3958	23.1772	27.5088	26.9004	27.9336	26.8108	0.01420003	0.035887805	0.014350427	0.026856917
mmu-miR-26a	23.3678	25.978	23.4862	22.8318	27.5329	28.7977	27.6424	25.8214	0.01858056	0.047213312	0.018695534	0.041968116
mmu-let-7e	24.3271	26.5386	25.4938	25.9586	27.659	28.5214	29.043	27.7452	0.03310373	0.08433279	0.028474958	0.096618114
mmu-miR-126-3p	23.0787	27.4536	28.4147	28.38	27.9921	30.9618	32.5924	30.2257	0.01106109	0.029295797	0.018418987	0.092740126
mmu-miR-30b	23.9855	25.7111	23.9361	23.1996	28.0337	28.9623	27.5402	26.0703	0.0201488	0.03500822	0.027411736	0.045573453
mmu-miR-378	23.968	24.584	25.483	26.286	28.041	28.215	30.093	28.926	0.0198054	0.026905361	0.013649931	0.053476079
mmu-miR-30c	23.8195	25.7939	23.9648	23.5957	28.0435	28.4398	27.5392	26.1903	0.01783726	0.053257832	0.027981895	0.05518567
mmu-miR-706	28.86	29.117	28.698	27.188	28.087	26.617	28.88	28.197	0.56960649	1.885618083	0.293826719	0.165630182
mmu-miR-374	23.668	24.734	25.012	25.389	28.339	27.605	30.999	28.545	0.01308482	0.045563977	0.005255477	0.037396252
mmu-miR-155	24.9183	27.9894	29.4906	31.1816	28.3517	30.2095	33.4423	34.7241	0.03085477	0.071542161	0.02154262	0.028607505
mmu-let-7g	24.375	26.1648	24.4222	24.0958	28.5565	28.0894	28.7336	26.6287	0.01837054	0.087804428	0.016788737	0.057597001
mmu-miR-146a	23.7733	28.5178	25.8689	27.2265	28.6363	30.9923	30.8941	29.8792	0.01145433	0.059976348	0.010236295	0.053007397
mmu-miR-140	24.8526	26.474	24.3022	23.6969	28.7586	29.2126	28.1987	26.1423	0.02223594	0.049943388	0.022382849	0.06119839
mmu-miR-26b	24.3165	27.1548	23.99	23.172	28.8272	30.1285	27.9419	26.0292	0.01462254	0.042433206	0.021539633	0.046001908
mmu-miR-196b	25.1373	29.3817	33.6549	32.7207	28.8547	31.4708	40	40	0.02534136	0.07834242	0.004100278	0.002145809
mmu-miR-30e	25.1989	27.1459	25.8025	25.7197	28.9673	30.2463	28.9978	27.9876	0.02446117	0.038865597	0.036391303	0.069210632
mmu-miR-15b	25.5832	26.7096	24.1508	24.1211	29.0852	28.8269	27.6094	26.3729	0.02942197	0.076825949	0.030320503	0.069987326
mmu-miR-20b	25.7618	27.008	28.4028	28.8495	29.3027	30.8014	33.9656	31.928	0.02863925	0.024040946	0.007051948	0.039460075
mmu-miR-135a*	27.664	28.136	27.639	27.649	29.338	29.377	29.013	28.112	0.10446109	0.141026433	0.128606681	0.241825368
mmu-miR-93	24.4857	24.8895	23.2559	23.4798	29.3809	28.2211	27.7962	26.4484	0.01120151	0.033110612	0.014325581	0.042583475
mmu-miR-29c	25.5342	28.1025	26.4836	25.7662	29.5006	31.5616	30.701	27.9091	0.02132423	0.030309997	0.017919044	0.07547473
mmu-let-7i	25.0966	26.366	25.7818	25.7885	29.5601	28.9023	30.0075	28.8162	0.01510885	0.057461422	0.01781625	0.040874291
mmu-miR-467a*	29.306	30.754	30.581	28.271	29.662	28.815	31.858	30.663	0.26044263	1.278132585	0.137550901	0.063506035
mmu-miR-93*	25.429	26.453	25.662	26.19	29.797	30.881	30.366	28.987	0.01614283	0.015485239	0.012788913	0.047962062
mmu-miR-150	24.1423	29.6036	25.3172	26.4567	29.8922	32.3733	29.8152	29.5635	0.00619422	0.048878286	0.014751827	0.038693566
mmu-miR-328	26.9547	28.617	27.4825	26.4567	29.9756	31.4928	30.0211	28.7345	0.0410674	0.045412633	0.057369888	0.068737324
mmu-miR-106b	25.3218	26.3962	25.4874	25.2577	30.0839	29.8886	29.1833	27.8009	0.01228411	0.0296184	0.025721837	0.057187256
mmu-miR-203	26.016	30.3413	30.9769	30.8906	30.1232	40	37.3541	32.5804	0.01934142	0.000412402	0.004010054	0.103323298
mmu-miR-877*	28.945	29.231	28.894	29.596	30.262	31.369	40	33.007	0.13378956	0.07573151	0.000151231	0.031337578
mmu-let-7d	26.7381	27.9427	28.2943	28.9079	30.3298	30.7216	31.146	30.7642	0.02764836	0.048567583	0.046177616	0.092061228
mmu-miR-760	30.73	30.39	31.301	30.609	30.618	29.809	32.058	31.438	0.3602418	0.498628586	0.197241837	0.187639764
mmu-miR-101b	26.201	27.145	27.606	28.638	30.631	31.021	34.754	30.889	0.01546379	0.022703169	0.002350262	0.070026146
mmu-miR-30a	25.8018	28.0745	26.2458	26.5641	30.7485	31.5962	30.7158	28.7654	0.01080871	0.02902294	0.015040929	0.072480539
mmu-miR-126-5p	24.7834	29.0801	30.3579	29.6498	30.9296	32.7208	36.5305	31.6185	0.00470639	0.026725069	0.004621053	0.085161047
mmu-miR-125b-5p	26.9582	30.3955	31.324	33.0094	30.9499	34.9873	33.1714	33.5701	0.02095354	0.01382322	0.09263091	0.225991043
mmu-miR-320	27.4347	29.4176	29.2999	29.612	31.016	31.7286	31.5271	31.4649	0.02784839	0.067173569	0.071190942	0.092278445
mmu-miR-340-5p	26.8334	28.061	25.2153	24.3687	31.0827	30.5887	29.8504	27.4066	0.01752718	0.057804976	0.013414504	0.040586324
mmu-miR-30d	26.4424	28.6008	27.0477	27.4724	31.1008	32.3871	31.2142	30.5693	0.0131996	0.024159552	0.018562534	0.03896
mmu-miR-25	27.1045	28.3196	27.5742	27.3563	31.1639	30.9382	31.113	29.9524	0.01999298	0.054275221	0.028680967	0.055128322
mmu-let-7c	26.9458	28.9004	28.6403	29.1658	31.2279	31.2024	32.5316	31.3515	0.01713319	0.06759393	0.022463671	0.073268531
mmu-miR-195	27.2015	28.7738	26.0695	24.3101	31.3148	30.693	29.5853	27.6452	0.01925981	0.088133695	0.029141875	0.033030383
mmu-miR-744	27.9324	28.983	27.1911	26.6223	31.6225	32.2509	30.0142	28.3172	0.02582545	0.034605316	0.047102175	0.102958689
mmu-miR-7a*	27.252	28.37	27.362	27.976	31.636	31.795	32.447	30.793	0.01596479	0.031034947	0.009820672	0.047301754
mmu-miR-31	29.4424	32.9522	29.326	34.2532	31.7498	35.321	31.6541	40	0.0673414	0.064535532	0.066382073	0.006207541
mmu-miR-130a	27.6072	30.8395	32.6682	33.685	31.7789	35.4874	40	34.2714	0.01849575	0.013296013	0.002069126	0.222000911
mmu-let-7b	28.7835	30.4957	31.0644	30.658	31.7807	33.4042	32.7641	32.0187	0.04174761	0.044394892	0.102616704	0.12979777
mmu-miR-148a	28.3028	28.3433	27.7608	27.4033	31.8118	31.0153	31.3992	29.9565	0.02927956	0.052303	0.026767709	0.056792235
mmu-miR-30e*	26.67	27.93	26.682	26.867	31.827	32.712	31.46	29.814	0.00934258	0.012115834	0.012149473	0.043225831
mmu-miR-301a	29.2922	29.732	27.474	26.9884	31.9682	32.1119	31.1012	29.4397	0.05215819	0.064040905	0.026976323	0.060948626
mmu-miR-322	27.4961	29.9939	27.7326	27.5927	32.0023	33.3806	31.5017	29.7388	0.01466822	0.031869882	0.024449309	0.075307507
mmu-miR-467b*	31.779	32.475	32.809	30.214	32.115	31.693	32.504	31.943	0.26407827	0.573170991	0.411806212	0.10055366
mmu-miR-331-3p	27.6022	30.0062	29.5357	29.2402	32.1171	33.2897	35.113	31.5108	0.01458003	0.034233141	0.006981426	0.069081226
mmu-miR-125a-5p	27.2273	34.9389	33.9711	35.2298	32.119	40	35.4149	40	0.01122872	0.009984719	0.122532597	0.012215337
mmu-miR-139-5p	27.0946	27.9646	22.5182	21.7069	32.1403	31.1046	26.7854	24.5334	0.01009187	0.037813298	0.017311055	0.0469913
mmu-miR-221	26.8785	27.3976	27.8856	27.4712	32.155	31.3629	31.8159	30.0956	0.00859991	0.021340496	0.02186455	0.054057459
mmu-miR-21	26.0911	27.8029	27.4157	27.6244	32.1961	32.2824	35.0805	31.2845	0.00484273	0.014942212	0.001642646	0.026368101
mmu-miR-378*	29.622	30	33.912	31.16	32.22	33.143	37.931	37.214	0.05505577	0.037734749	0.020560761	0.005016989
mmu-miR-532-3p	40	31.6458	29.7828	30.122	32.2846	35.5646	33.28	40	70.0560383	0.022039534	0.02952002	0.000354246
mmu-miR-138	31.0257	34.2785	31.938	31.1189	32.2906	33.9305	40	33.3081	0.1387094	0.424264978	0.001247311	0.073090996
mmu-miR-181a	27.5224	29.5408	29.4902	29.8364	32.2978	32.9902	35.486	32.2974	0.01217139	0.030514473	0.005223518	0.06054021
mmu-miR-130b*	27.077	28.162	29.234	29.406	32.418	32.405	35.543	32.944	0.00822389	0.017603883	0.004204172	0.028696876
mmu-miR-34c*	32.918	32.116	31.596	32.305	32.557	31.983	33.934	34.21	0.42810527	0.365523863	0.065928108	0.089005451
mmu-miR-678	32.285	31.752	31.9	31.718	32.581	31.897	32.857	31.681	0.27150252	0.301459793	0.171708992	0.341992715
mmu-miR-877	31.261	31.309	31.723	31.711	32.66	31.267	33.128	31.917	0.1263973	0.343180028	0.125872715	0.288979188
mmu-miR-128a	30.6468	34.3789	32.6334	40	32.7138	40	40	33.1871	0.07955175	0.006772656	0.002019813	37.47710217
mmu-miR-27a	27.9999	28.5843	26.9952	26.6667	32.7229	29.2659	30.2481	28.8186	0.01262159	0.207824479	0.034966992	0.07500536
mmu-miR-186	27.2715	28.4775	27.6765	27.7582	32.7637	31.4959	32.3202	31.8952	0.00740563	0.041138628	0.013334777	0.018946005
mmu-miR-692	31.583	30.678	30.741	30.017	32.821	32.912	36.481	32.383	0.14131999	0.07085618	0.006236868	0.064660905
mmu-let-7f	29.0859	31.0088	30.2066	31.3047	32.8377	34.3531	40	34.9115	0.02474426	0.03282042	0.00037564	0.027360483
mmu-miR-193b	30.4091	32.3159	40	40	32.8483	40	40	37.956	0.06146196	0.001620818	0.333333333	1.374624425

mmu-miR-425*	29.572	29.7	30.108	30.592	32.851	32.101	32.893	32.724	0.03434009	0.063111096	0.048362663	0.076047124
mmu-miR-130b	27.7916	28.5559	29.9365	29.8783	33.0105	31.7178	32.9666	31.2192	0.00895021	0.03724363	0.04080635	0.131591435
mmu-miR-297a*	31.77	32.616	34.204	30.251	33.155	31.548	32.442	31.612	0.12762983	0.69884165	1.130559953	0.129770782
mmu-miR-494	30.3912	29.9902	28.438	29.6052	33.1616	29.8733	32.3621	31.8497	0.04885458	0.361467414	0.021958716	0.070342358
mmu-miR-24-2*	29.021	30.377	27.918	28.545	33.187	35.173	34.007	31.938	0.01856897	0.01199883	0.004896741	0.031731015
mmu-miR-652	26.4738	27.735	25.0178	24.5715	33.227	33.3724	30.3567	28.2581	0.00309003	0.006696567	0.008235874	0.025888183
mmu-miR-342-3p	27.9647	30.3673	28.2328	29.32	33.2557	32.5568	32.8212	32.2772	0.00851391	0.073075799	0.013855835	0.042921297
mmu-miR-224	29.5089	36.7061	40	40	33.2693	40	40	40	0.02459719	0.033987251	0.333333333	0.333333333
mmu-miR-30a*	27.943	30.167	29.338	30.482	33.301	32.466	32.895	32.375	0.00812756	0.067734633	0.028321422	0.089748865
mmu-miR-674*	29.681	29.667	30.436	31.081	33.327	33.388	33.331	33.13	0.02662707	0.025278199	0.044812266	0.080550508
mmu-miR-188-5p	31.9629	32.9698	31.1746	31.96	33.3434	32.9636	31.6634	33.5023	0.12802855	0.33476892	0.237539197	0.114445885
mmu-miR-301b	28.7046	28.8977	26.9685	26.6517	33.35	30.2625	30.8524	28.6499	0.01331907	0.129429421	0.022579189	0.08343737
mmu-miR-331-5p	35.8779	40	40	40	33.364	35.257	33.3407	40	1.90387334	8.926146589	33.69207784	0.333333333
mmu-miR-879*	34.576	40	40	40	33.481	40	40	40	0.71204361	0.333333333	0.333333333	0.333333333
mmu-miR-667	40	34.8764	32.6891	33.2164	33.5375	32.6348	40	40	29.395787	1.576404201	0.00209932	0.003025601
mmu-miR-101a	29.0553	30.184	31.1185	31.5983	33.575	34.8878	34.3709	37.1481	0.0145316	0.012790686	0.034979113	0.007115779
mmu-miR-674	31.8962	40	33.5729	34.8117	33.5953	40	40	40	0.10265939	0.333333333	0.003873726	0.009142076
mmu-miR-107	31.0987	40	33.3585	40	33.6406	40	33.7373	40	0.05723881	0.333333333	0.256359008	0.333333333
mmu-miR-700	31.714	33.185	30.701	31.831	33.653	35.166	33.987	33.582	0.08693238	0.084438075	0.034173871	0.099031925
mmu-miR-27b	28.9134	28.8566	29.0116	29.6346	33.6569	31.5933	35.098	31.4924	0.01244351	0.050009206	0.004905573	0.09196556
mmu-miR-21*	35.635	33.439	36.817	36.151	33.756	34.692	40	34.995	1.22606673	0.139858273	0.036702891	0.742795767
mmu-miR-18a	29.1798	27.7036	28.3486	28.3147	33.7826	30.5274	32.521	31.0957	0.01371822	0.047079327	0.018486777	0.048496939
mmu-miR-671-3p	31.4071	32.4674	31.5758	32.1273	33.7836	34.4199	33.5366	33.6818	0.06419201	0.086122708	0.085628656	0.113482166
mmu-miR-15b*	27.55	27.872	25.418	25.904	33.785	29.93	30.524	29.523	0.00442544	0.080049572	0.009678757	0.027130087
mmu-miR-503*	30.407	32.682	30.511	30.291	33.884	35.162	34.586	33.866	0.02993625	0.059748135	0.019777961	0.02797026
mmu-miR-532-5p	31.4072	29.6588	27.2933	26.7775	33.9081	33.8524	36.3567	31.8352	0.05888882	0.018217105	0.000623051	0.010008277
mmu-miR-18a*	30.868	29.256	31.778	31.195	33.917	34.58	34.494	40	0.04027525	0.008321374	0.050731917	0.000745263
mmu-miR-103	28.2437	29.8793	28.7277	28.2023	33.9574	40	33.1526	30.3947	0.00635161	0.000299395	0.015518549	0.072929054
mmu-miR-99b	29.9871	36.8118	40	37.1575	34.0424	40	40	40	0.02004988	0.036570838	0.333333333	0.046473029
mmu-miR-339-5p	31.1818	31.8164	30.9697	31.1478	34.1006	40	36.6805	36.2254	0.04407907	0.001146488	0.006364388	0.009871175
mmu-miR-98	30.5037	31.4575	30.2238	30.2134	34.1078	34.9763	35.6482	32.7636	0.02741174	0.029081339	0.007761964	0.056910454
mmu-miR-29b	30.7753	32.0856	30.7789	31.0736	34.2285	35.4744	33.9805	32.5028	0.03043421	0.031823526	0.036232734	0.123778916
mmu-miR-324-3p	30.1461	32.3857	32.7212	31.9017	34.3492	40	40	40	0.01809754	0.001701163	0.002146553	0.001216319
mmu-miR-455*	34.532	40	40	40	34.391	40	40	33.679	0.36755639	0.333333333	0.333333333	26.64951735
mmu-miR-322*	29.387	32.89	28.787	29.09	34.458	35.439	36.34	33.838	0.00991644	0.05695781	0.001775003	0.012404759
mmu-miR-149	28.425	26.323	24.148	25.262	34.5	31.289	28.634	28.397	0.00494449	0.010665072	0.014875041	0.037944576
mmu-miR-340-3p	28.7298	30.2838	27.5554	26.2755	34.5137	35.5376	31.6481	29.6863	0.00604994	0.008736296	0.019536793	0.031341923
mmu-miR-191*	34.515	32.486	35.681	34.604	34.52	34.152	40	40	0.33218009	0.105041949	0.016700528	0.007916275
mmu-miR-15a	30.3671	31.6195	28.5836	29.0231	34.5365	40	33.8913	31.0349	0.01852526	0.001000223	0.008415925	0.082654518
mmu-miR-450a-5p	31.2726	34.1701	31.7909	32.6809	34.6019	40	40	40	0.03316344	0.005860086	0.001126402	0.002087421
mmu-miR-146b*	33.811	33.084	34.269	36.603	34.617	34.918	40	40	0.19065516	0.09349529	0.006275897	0.03164316
mmu-miR-23b	31.2913	31.6358	32.2179	31.7653	34.6535	40	40	40	0.03241572	0.001011588	0.001514374	0.001106591
mmu-miR-764-5p	35.782	33.438	33.961	35.525	34.764	36.359	37.078	35.182	0.67503654	0.044011901	0.038420963	0.422797133
mmu-miR-16*	30.984	30.314	29.48	30.65	34.771	36.464	37.421	33.937	0.02414783	0.004694013	0.001356437	0.034150192
mmu-miR-152	29.5778	32.8787	32.7217	40	34.7757	40	40	40	0.00908144	0.002394164	0.002147297	0.333333333
mmu-miR-574-3p	40	28.7798	27.5965	26.3879	34.8043	31.5311	32.4491	40	12.216317	0.049505667	0.011537204	2.66212E-05
mmu-let-7g*	34.339	35.975	33.922	35.489	34.818	32.744	34.297	40	0.23915826	3.129728475	0.257035138	0.014619497
mmu-miR-365	31.0787	40	30.4013	29.3906	34.8208	38.2348	35.8297	31.5951	0.02491119	1.133070398	0.007740473	0.07231995
mmu-miR-296-5p	31.3642	31.2618	29.5349	29.6729	34.8554	33.9538	33.2661	32.582	0.02964305	0.051582929	0.02510011	0.044376432
mmu-miR-33*	29.392	32.189	30.997	30.053	34.857	36.949	34.421	33.352	0.00754657	0.012302007	0.031056466	0.033867317
mmu-miR-872	29.7369	32.5034	33.5469	31.6725	34.859	33.0208	33.4397	36.6972	0.00957135	0.232876586	0.35904523	0.010239844
mmu-miR-20a*	28.113	30.648	30.363	31.083	34.88	34.511	40	34.285	0.00306062	0.02290867	0.000418652	0.03622269
mmu-miR-142-5p	30.0843	30.9562	28.6391	28.3794	34.9084	40	32.9965	31.5118	0.01176738	0.000631573	0.016261876	0.038013021
mmu-let-7d*	31.621	32.908	34.877	40	34.981	40	38.295	36.379	0.03246519	0.002443284	0.031185896	4.10117555
mmu-miR-193*	33.683	34.987	30.597	31.263	35.058	40	33.557	32.788	0.12851757	0.010323225	0.042838076	0.115826518
mmu-miR-299*	40	40	40	40	35.15	36.715	40	40	9.61333827	3.249094036	0.333333333	0.333333333
mmu-miR-185	31.4475	31.8276	30.947	30.1177	35.1933	35.6075	34.6459	35.6624	0.02484738	0.024266965	0.025668406	0.007140979
mmu-miR-503	30.2249	31.5445	29.0715	28.556	35.1943	36.3348	35.1932	32.6194	0.01063997	0.01204633	0.004787	0.019937627
mmu-miR-324-5p	33.0034	32.6831	34.5174	34.1024	35.2287	40	36.0466	33.7806	0.07128476	0.002090607	0.115489812	0.416629673
mmu-miR-369-5p	40	40	40	40	35.252	40	40	40	8.95713592	0.333333333	0.333333333	0.333333333
mmu-miR-26b*	32.118	32.808	30.25	29.783	35.44	38.535	34.926	33.818	0.03333167	0.006293322	0.013039546	0.020333995
mmu-miR-206	35.937	36.47	34.132	33.98	35.501	35.278	35.067	33.578	0.45094941	0.761564165	0.17434749	0.440446135
mmu-miR-744*	33.535	34.333	30.732	30.412	35.775	33.205	33.734	31.314	0.07056211	0.728518493	0.041608944	0.17838145
mmu-let-7a*	31.997	31.938	32.961	33.176	35.805	36.394	36.156	35.289	0.02379888	0.015187598	0.036398871	0.077055273
mmu-miR-467a	30.2335	31.0111	31.6947	28.0668	35.8923	34.53	34.5662	30.3764	0.00659797	0.029079323	0.045548189	0.067238787
mmu-miR-15a*	29.739	31.173	28.579	29.005	35.9	34.172	32.452	31.493	0.00465836	0.041695558	0.022750428	0.059417738
mmu-miR-326	33.118	35.948	33.362	32.801	35.953	40	35.06	34.576	0.04671525	0.020095796	0.102737694	0.097398104
mmu-miR-351	31.9156	34.4826	32.5977	33.907	35.965	40	35.5339	40	0.02013204	0.007277393	0.043550633	0.004883183
mmu-miR-192	30.1915	31.6425	28.049	27.7311	36.0352	34.0501	33.5543	30.7792	0.0058043	0.062823037	0.007338686	0.040300387
mmu-miR-704	33.416	32.118	31.758	32.761	36.097	34.622	37.833	35.827	0.05197773	0.058762415	0.00494449	0.039803456
mmu-miR-761	40	40	40	40	36.177	40	40	40	4.71754899	0.333333333	0.333333333	0.333333333
mmu-miR-721	33.582	35.933	38.92	36.814	36.256	35.666	34.748	36.419	0.05223054	0.401101009	6.008635885	0.438314253
mmu-miR-199a-3p	32.3587	35.0136	33.1778	33.373	36.3242	35.7616	40	34.0453	0.02133754	0.198476142	0.002945723	0.209168499
mmu-miR-125b*	33.629	38.388	37.026	40	36.327	36.831	40	40	0.05136885	0.980804497	0.042424383	0.333333333
mmu-miR-133a	35.2024	35.4252	36.3197	33.6312	36.4713	35.0217	34.1239	33.1047	0.13832535	0.440904314	1.527145475	0.480143482
mmu-miR-17*	30.282	31.33	33.405	33.042	36.551	40	40	35.482	0.00432237	0.000818368	0.003448148	0.061427884
mmu-miR-708	40	40	35.8635	39.1321	36.6679	40	40	40	3.35691847	0.333333333	0.018952573	0.182647954
mmu-miR-698	40	40	40	35.535	36.689	38.047	40	40	3.30817946	1.290596036	0.333333333	0.015093147
mmu-miR-467b	30.798	32.3867	33.3336	30.7237	36.7799	35.0416	35.341	40	0.00527409	0.052926626	0.082906987	0.000537569
mmu-miR-141	40	38.5941	33.3296	32.8004	36.8999	40	40	34.7563	2.85826068	0.125794216	0.003272565	0.085919982
mmu-miR-542-3p	40	35.6185	32.3562	32.2522	36.9461	40	36.7786	40	2.76817951	0.01599248	0.015545464	0.00155081
mmu-miR-343	40	40	40	40	36.949	36.747	40	40	2.7626207	3.177820199	0.333333333	0.333333333
mmu-miR-148b	32.9817	32.9683	32.2017	30.6418	36.9866	35.8752	40	32.648	0.02076269	0.044444155	0.001497464	0.082975976
mmu-miR-181a-1*	29.491	30.593	32.535	33.431	37.008	35.781	35.134	40	0.00181985	0.009143977	0.055017618	0.003510853
mmu-miR-29a*	31.985	34.293	32.582	32.329	37.093	40	40	40	0.00966535	0.006381174	0.001949118	0.001635602
mmu-miR-872*	28.933	30.189	30.456	31.427	37.097	33.461	40	33.308	0.00116217	0.034507111	0.000446528	0.090498488
mmu-miR-875-5p	35.411	35.521	35.938	34.847	37.198	38.234	40	37.52	0.09659133	0.050837521	0.019956984	0.052266759
mmu-miR-676	30.4695	33.5439	40	40	37.4018	40	40	40	0.00272928	0.003796636	0.333333333	0.333333333
mmu-miR-542-5p	40	35.6373	32.253	32.8137	37.5361	40	35.5591	40	1.83902042	0.016202245	0.033701053	0.00228869
mmu-miR-127	32.3217	40	40	35.6275	37.5476	40	40	40	0.00890689	0.333333333	0.333333333	0.016092558
mmu-miR-181c	31.2105	33.8075	33.181	34.4456	37.6513	40	32.5874	37.247	0.00383711	0.00455775	0.503002508	0.047816008

mmu-miR-28	30.1992	32.5052	31.524	30.2613	37.6871	40	40	31.9544	0.00185693	0.001848073	0.000936157	0.103087228
mmu-miR-186*	31.524	34.035	31.003	32.083	37.86	40	34.722	36.5	0.00412622	0.005336233	0.025313266	0.01560376
mmu-miR-294	40	37.8906	40	37.3332	38.1218	39.3356	40	38.6108	1.22538704	0.122430719	0.333333333	0.137493708
mmu-miR-29c*	33.972	40	35.13	33.19	39.924	40	35.156	40	0.00538454	0.333333333	0.327379865	0.002970739
mmu-miR-590-5p	35.6278	40	40	40	39.9505	40	40	40	0.01665775	0.333333333	0.333333333	0.333333333
mmu-miR-696	40	40	40	40	40	29.801	40	40	0.33333333	391.8173573	0.333333333	0.333333333
mmu-miR-425	27.9643	28.93	28.2622	27.6068	40	32.1531	40	30.4625	7.9391E-05	0.035696774	9.75998E-05	0.046049762
mmu-miR-124	40	40	40	40	40	32.2933	40	32.96	0.33333333	69.63484494	0.333333333	43.86618994
mmu-miR-680	34.6746	36.2639	40	33.8764	40	32.9151	40	37.1171	0.0083133	3.396002449	0.333333333	0.03526394
mmu-miR-339-3p	30.9585	32.3243	31.2382	31.1933	40	32.9275	40	32.8316	0.00063258	0.219430732	0.000767917	0.10707826
mmu-miR-105	40	40	40	40	40	33.3834	40	40	0.33333333	32.70949315	0.333333333	0.333333333
mmu-miR-451	40	40	40	33.3398	40	33.6612	40	40	0.33333333	26.98035637	0.333333333	0.003295784
mmu-miR-24-1*	40	40	40	40	40	33.674	40	40	0.33333333	26.74203777	0.333333333	0.333333333
mmu-miR-190b	33.41	33.828	32.121	31.007	40	34.072	33.35	34.122	0.00346012	0.281466962	0.14220435	0.038474263
mmu-miR-350	32.0779	34.9018	30.7401	30.6189	40	34.2205	36.8427	31.9034	0.00137432	0.534528027	0.004850797	0.136837684
mmu-miR-673-5p	35.687	35.544	35.858	35.823	40	34.277	35.769	36.289	0.01677013	0.802202017	0.354544224	0.241323029
mmu-miR-677	40	40	40	40	40	34.6801	40	40	0.33333333	13.31460293	0.333333333	0.333333333
mmu-miR-470*	37.41	35.398	35.292	34.668	40	34.7	40	39.305	0.05536191	0.540751437	0.012753504	0.013396849
mmu-miR-145	36.4334	36.2997	37.7372	33.3274	40	34.7575	40	40	0.02813359	0.970794271	0.069455729	0.003267578
mmu-miR-763	40	37.309	40	40	40	34.768	40	40	0.33333333	1.939974245	0.333333333	0.333333333
mmu-miR-135a	33.2792	32.5418	28.4207	27.0401	40	34.7714	33.0831	30.0611	0.00316021	0.07107261	0.013163049	0.041064556
mmu-miR-7a	36.1924	40	35.3622	32.9866	40	34.9443	40	40	0.02380548	11.08654169	0.013389422	0.002580091
mmu-miR-467c	31.8375	34.0483	34.4851	30.131	40	35.0874	34.2324	32.8213	0.00116338	0.162210318	0.397144932	0.051643747
mmu-miR-466d-5p	40	40	40	40	40	35.702	40	40	0.33333333	6.557007292	0.333333333	0.333333333
mmu-miR-682	31.8451	31.442	40	34.6033	40	35.7496	35.0959	34.4848	0.00116952	0.016833016	9.980675761	0.361868516
mmu-miR-7b	34.6829	36.0895	34.0555	33.3718	40	35.8007	40	36.7897	0.00836127	0.407207912	0.0054126	0.031188057
mmu-miR-335-3p	40	40	40	40	40	36.0736	40	40	0.33333333	5.068073682	0.333333333	0.333333333
mmu-miR-467d	35.9954	34.7478	40	32.1562	40	36.08	40	36.1932	0.02076701	0.132387379	0.333333333	0.020305826
mmu-miR-881*	40	40	36.526	36.431	40	36.257	40	40	0.33333333	4.463073294	0.029998569	0.028086828
mmu-miR-27a*	30.914	30.298	29.401	29.147	40	36.614	40	34.244	0.00061337	0.004183823	0.000214913	0.009739325
mmu-miR-22	32.232	32.816	28.816	27.758	40	36.815	35.298	30.979	0.00152925	0.020847779	0.003729085	0.035748772
mmu-miR-694	36.861	37.466	35.325	35.761	40	36.98	40	40	0.03783952	0.466852107	0.013048588	0.017652759
mmu-miR-410	34.6558	36.8967	40	37.0341	40	37.0524	40	40	0.00820567	0.299232234	0.333333333	0.042663245
mmu-miR-29b*	32.644	33.307	31.882	31.719	40	37.16	35.197	33.704	0.00203471	0.023068012	0.033493791	0.084204287
mmu-let-7c-1*	36.915	35.749	38.861	40	40	37.281	40	40	0.03928269	0.115265886	0.15135807	0.333333333
mmu-miR-138*	37.007	36.46	40	36.847	40	37.928	38.218	39.046	0.04186933	0.120494358	1.146341982	0.072596183
mmu-miR-218-1*	38.64	34.477	40	36.676	40	38.291	40	40	0.12986076	0.023700109	0.333333333	0.033285497
mmu-miR-196a*	38.852	35.378	37.094	38.326	40	38.505	40	40	0.15041679	0.03815557	0.044471889	0.104461085
mmu-miR-434-3p	33.3492	34.8797	36.3579	35.8083	40	38.6868	39.0199	40	0.00331733	0.023813731	0.052666796	0.018241112
mmu-miR-676*	34.064	36.986	40	40	40	38.947	40	40	0.00544458	0.085616787	0.333333333	0.333333333
mmu-miR-338-3p	34.8309	40	33.7315	32.2118	40	39.0381	35.4574	34.0723	0.00926456	0.649291172	0.100769958	0.091793608
mmu-miR-99b*	34.573	37.842	40	40	40	39.19	38.237	40	0.00774799	0.130945419	1.131343869	0.333333333
mmu-miR-135b	32.4777	34.5669	29.2955	26.4154	40	40	31.6866	29.6921	0.00181318	0.007715297	0.063545664	0.034394877
mmu-miR-200c	32.4302	33.8826	28.8582	28.1033	40	40	32.0913	30.23	0.00175445	0.004801289	0.035450198	0.076327011
mmu-miR-194	31.024	33.166	29.7149	29.4844	40	40	34.3312	31.4749	0.00066196	0.002921728	0.013590454	0.083883886
mmu-miR-218	33.0046	40	40	40	40	40	34.4105	35.917	0.00261248	0.333333333	16.0504017	5.649164452
mmu-miR-361	31.9761	40	32.8962	30.4476	40	40	34.4472	33.5111	0.00128069	0.333333333	0.11375781	0.03987249
mmu-miR-31*	30.158	33.509	30.776	40	40	40	34.738	40	0.0003632	0.003705894	0.021389366	0.333333333
mmu-miR-133b	40	40	40	40	40	40	34.7842	40	0.33333333	0.333333333	12.38770905	0.333333333
mmu-miR-669a	33.2967	34.4529	34.0454	33.2319	40	40	35.2694	40	0.00319878	0.007129109	0.142698047	0.003058283
mmu-let-7i*	34.159	34.155	37.156	40	40	40	35.284	40	0.00581517	0.005799071	1.220132224	0.333333333
mmu-miR-500	33.9803	40	40	33.1843	40	40	35.4285	40	0.0051377	0.333333333	7.925695271	0.002959025
mmu-miR-1	35.1916	37.7304	35.2127	35.3834	40	40	35.8876	33.7928	0.01189614	0.069129126	0.208791878	1.003915261
mmu-miR-30b*	33.974	33.633	33.15	33.666	40	40	36.059	40	0.00511531	0.004038506	0.044379508	0.004131947
mmu-miR-129-3p	40	40	40	32.3283	40	40	40	31.8811	0.33333333	0.333333333	0.333333333	0.454463867
mmu-miR-147	40	40	31.9068	31.7986	40	40	40	32.6321	0.33333333	0.333333333	0.001220626	0.187055397
mmu-miR-491	33.175	32.622	40	32.9545	40	40	40	32.7517	0.00294001	0.002003916	0.333333333	0.383643309
mmu-miR-345-3p	40	40	40	40	40	40	40	33.3757	0.33333333	0.333333333	0.333333333	32.88453806
mmu-miR-106b*	28.433	28.315	27.092	27.341	40	40	40	34.066	0.00010987	0.000101238	4.33694E-05	0.003151026
mmu-miR-423-5p	34.7184	40	32.9472	40	40	40	40	34.0687	0.00856956	0.333333333	0.002510582	20.34126504
mmu-miR-467e	32.9461	40	40	32.6789	40	40	40	34.0688	0.00250867	0.333333333	0.333333333	0.127197084
mmu-miR-183*	40	40	40	40	40	40	40	34.165	0.33333333	0.333333333	0.333333333	19.02780841
mmu-miR-702	35.431	33.568	33.229	35.825	40	40	40	34.216	0.01404341	0.003860591	0.003052141	1.016801102
mmu-miR-672	33.7055	40	40	40	40	40	40	34.5076	0.00424664	0.333333333	0.333333333	15.00568735
mmu-miR-345-5p	32.6073	35.6667	33.275	31.7129	40	40	40	34.5233	0.0019836	0.01653581	0.003151026	0.047518645
mmu-miR-380-5p	40	40	40	40	40	40	40	34.5751	0.33333333	0.333333333	0.333333333	14.31978076
mmu-miR-34a	29.7599	40	32.7557	32.6641	40	40	40	34.7868	0.00027561	0.333333333	0.002198503	0.076538928
mmu-miR-22*	40	34.829	32.241	31.434	40	40	40	34.789	0.33333333	0.009252362	0.001538817	0.032577902
mmu-miR-335-5p	40	40	40	40	40	40	40	34.8834	0.33333333	0.333333333	0.333333333	11.56455221
mmu-miR-383	40	40	40	40	40	40	40	34.8912	0.33333333	0.333333333	0.333333333	11.50219662
mmu-miR-384-5p	36.3723	40	40	40	40	40	40	35.5664	0.02696697	0.333333333	0.333333333	7.20319769
mmu-miR-878-3p	36.303	40	35.626	40	40	40	40	35.62	0.02570223	0.333333333	0.016075835	6.940489896
mmu-let-7b*	40	40	40	40	40	40	40	36.052	0.33333333	0.333333333	0.333333333	5.144523651
mmu-let-7f*	40	40	40	40	40	40	40	36.123	0.33333333	0.333333333	0.333333333	4.897472866
mmu-miR-362-3p	34.293	36.2056	31.9302	33.0535	40	40	40	36.3987	0.00638117	0.024024288	0.001240586	0.032799952
mmu-miR-19a*	40	33.4	40	40	40	40	40	37.648	0.33333333	0.003436219	0.333333333	1.701771692
mmu-miR-136	32.6835	40	36.1219	37.6609	40	40	40	38.5096	0.00209119	0.333333333	0.022670146	0.185094958
mmu-miR-10a*	40	35.189	40	40	40	40	40	40	0.33333333	0.011874722	0.333333333	0.333333333
mmu-miR-139-3p	40	40	29.5632	29.6151	40	40	40	40	0.33333333	0.333333333	0.000240485	0.000249294
mmu-miR-199b*	40	40	36.424	40	40	40	40	40	0.33333333	0.333333333	0.02795088	0.333333333
mmu-miR-200b*	40	40	40	35.951	40	40	40	40	0.33333333	0.333333333	0.333333333	0.020137627
mmu-miR-202-3p	40	40	40	35.2009	40	40	40	40	0.33333333	0.333333333	0.333333333	0.011973075
mmu-miR-208b	40	40	35.4135	40	40	40	40	40	0.33333333	0.333333333	0.013874095	0.333333333
mmu-miR-214	40	40	34.5304	40	40	40	40	40	0.33333333	0.333333333	0.00752255	0.333333333
mmu-miR-298	40	33.6473	40	40	40	40	40	40	0.33333333	0.004078735	0.333333333	0.333333333
mmu-miR-30c-1*	40	40	34.867	40	40	40	40	40	0.33333333	0.333333333	0.009499304	0.333333333
mmu-miR-327	40	35.522	40	40	40	40	40	40	0.33333333	0.014957755	0.333333333	0.333333333
mmu-miR-330	40	36.4419	34.9246	40	40	40	40	40	0.33333333	0.028299836	0.009886239	0.333333333
mmu-miR-34b-3p	40	40	33.8682	40	40	40	40	40	0.33333333	0.333333333	0.004753604	0.333333333
mmu-miR-363	40	34.2941	40	40	40	40	40	40	0.33333333	0.006386041	0.333333333	0.333333333
mmu-miR-375	40	40	34.3779	40	40	40	40	40	0.33333333	0.333333333	0.006767963	0.333333333

mmu-miR-381	40	34.6036	40	40	40	40	40	40	0.33333333	0.007914081	0.333333333	0.333333333
mmu-miR-466b-3-3p	40	32.508	40	32.519	40	40	40	40	0.33333333	0.001851663	0.333333333	0.001865835
mmu-miR-467e*	40	33.953	40	33.521	40	40	40	40	0.33333333	0.005041391	0.333333333	0.003736848
mmu-miR-486	40	40	40	33.6425	40	40	40	40	0.33333333	0.333333333	0.333333333	0.004065187
mmu-miR-495	40	40	40	35.2927	40	40	40	40	0.33333333	0.333333333	0.333333333	0.012759694
mmu-miR-582-5p	40	35.8746	35.2211	40	40	40	40	40	0.33333333	0.019098955	0.012141896	0.333333333
mmu-miR-653	40	40	35.12	40	40	40	40	40	0.33333333	0.333333333	0.011320155	0.333333333
mmu-miR-717	40	40	40	36.383	40	40	40	40	0.33333333	0.333333333	0.333333333	0.027167724
mmu-miR-878-5p	40	40	36.0543	40	40	40	40	40	0.33333333	0.333333333	0.021632399	0.333333333
mmu-miR-497	36.2916	40	40	35.2838	40	40	40	40	0.02549994	0.333333333	0.333333333	0.012681221
mmu-miR-297b-5p	36.2218	40	40	40	40	40	40	40	0.02429558	0.333333333	0.333333333	0.333333333
mmu-miR-879	36.1441	40	40	40	40	40	40	40	0.02302169	0.333333333	0.333333333	0.333333333
mmu-miR-143	36.1293	35.2452	36.2706	40	40	40	40	40	0.02278673	0.012346428	0.025131446	0.333333333
mmu-miR-485*	36.108	40	40	34.672	40	40	40	40	0.02245277	0.333333333	0.333333333	0.008298334
mmu-miR-592	36.03	40	40	36.06	40	40	40	40	0.02127109	0.333333333	0.333333333	0.021718037
mmu-miR-665	35.9634	40	37.3969	40	40	40	40	40	0.02031146	0.333333333	0.054861486	0.333333333
mmu-miR-434-5p	35.8388	40	40	40	40	40	40	40	0.01863085	0.333333333	0.333333333	0.333333333
mmu-miR-101a*	35.596	40	40	40	40	40	40	40	0.015745	0.333333333	0.333333333	0.333333333
mmu-miR-376c	35.275	40	40	35.125	40	40	40	40	0.01260411	0.333333333	0.333333333	0.011359456
mmu-miR-9	35.1553	40	40	40	40	40	40	40	0.01160055	0.333333333	0.333333333	0.333333333
mmu-miR-9*	35.154	40	40	40	40	40	40	40	0.01159011	0.333333333	0.333333333	0.333333333
mmu-miR-182	34.9392	40	33.9858	40	40	40	40	40	0.0099868	0.333333333	0.005157321	0.333333333
mmu-miR-337-5p	34.746	40	38.1127	40	40	40	40	40	0.00873509	0.333333333	0.090104158	0.333333333
mmu-miR-376a	34.6674	40	40	40	40	40	40	40	0.00827192	0.333333333	0.333333333	0.333333333
mmu-miR-501-5p	34.483	36.71	40	40	40	40	40	40	0.00727941	0.034079252	0.333333333	0.333333333
mmu-miR-701	34.244	34.258	35.787	40	40	40	40	40	0.00616808	0.006228228	0.017973778	0.333333333
mmu-miR-132	33.6894	40	40	32.0051	40	40	40	40	0.00419951	0.333333333	0.333333333	0.001306694
mmu-miR-200a*	33.475	29.166	40	40	40	40	40	40	0.00361958	0.000182608	0.333333333	0.333333333
mmu-miR-376b*	33.417	40	40	40	40	40	40	40	0.00347695	0.333333333	0.333333333	0.333333333
mmu-miR-429	33.3828	35.0098	35.696	34.2843	40	40	40	40	0.00339549	0.010487666	0.016875072	0.006342808
mmu-miR-92a*	32.949	40	40	40	40	40	40	40	0.00251372	0.333333333	0.333333333	0.333333333
mmu-miR-411	32.6991	34.1268	40	35.6962	40	40	40	40	0.00211392	0.005686819	0.333333333	0.016877412
mmu-miR-27b*	32.649	31.476	33.178	35.102	40	40	40	40	0.00204177	0.000905522	0.002946131	0.011179795
mmu-miR-32	32.5898	35.0722	33.8831	33.6168	40	40	40	40	0.00195969	0.010951236	0.004802953	0.003993412
mmu-miR-330*	32.563	34.16	32.131	34.594	40	40	40	40	0.00192362	0.005819204	0.00142585	0.007861594
mmu-miR-379	32.4739	33.489	40	35.7872	40	40	40	40	0.00180841	0.003654874	0.333333333	0.01797627
mmu-miR-200b	31.1679	33.4597	32.9635	32.1893	40	40	40	40	0.00073139	0.003581395	0.002539108	0.001484649
mmu-miR-28*	30.794	32.031	33.343	31.602	40	40	40	40	0.00056441	0.001330365	0.003303103	0.000988164
mmu-miR-10b	28.2336	34.1619	29.7746	28.7284	40	40	40	40	9.5684E-05	0.005826873	0.000278437	0.000134831
mmu-miR-466d-3p	26.477	40	40	40	40	40	40	40	2.8317E-05	0.333333333	0.333333333	0.333333333
mmu-miR-699	25.837	26.135	26.166	27.171	24.263	26.342	28.205	29.213	0.99243017	0.288778952	0.081110781	0.080942291
mmu-miR-801	27.168	25.974	25.938	25.398	28.548	26.128	27.183	26.284	0.12807293	0.299585042	0.140635966	0.180370774
mmu-miR-805	26.359	25.239	28.599	30.847	29.538	29.135	32.588	31.124	0.03680479	0.022390608	0.020992787	0.275101803
mmu-miR-720	22.445	22.626	20.657	21.053	27.298	25.48	26.179	25.562	0.01153401	0.046104056	0.007254226	0.014639778
mmu-miR-685	33.1571	32.8285	30.6907	31.6102	34.0142	30.7108	32.0173	30.6332	0.18402039	1.446671638	0.132902256	0.656122681
mmu-miR-197	40	36.4647	34.7061	35.284	35.0739	34.672	40	38.0761	10.1340398	1.154875634	0.008496813	0.048125238
mmu-miR-715	40	40	40	40	40	31.604	34.821	40	0.33333333	112.2862528	12.07572162	0.333333333

Note: in red; miRNAs that have been removed from MiRbase
